# Posicionamento do Departamento de Imagem Cardiovascular da Sociedade Brasileira de Cardiologia sobre o Uso do *Strain* Miocárdico na Rotina do Cardiologista – 2023

**DOI:** 10.36660/abc.20230646

**Published:** 2023-12-12

**Authors:** André Luiz Cerqueira Almeida, Marcelo Dantas Tavares de Melo, David Costa de Souza Le Bihan, Marcelo Luiz Campos Vieira, José Luiz Barros Pena, José Maria Del Castillo, Henry Abensur, Renato de Aguiar Hortegal, Maria Estefania Bosco Otto, Rafael Bonafim Piveta, Maria Rosa Dantas, Jorge Eduardo Assef, Adenalva Lima de Souza Beck, Thais Harada Campos Espirito Santo, Tonnison de Oliveira Silva, Vera Maria Cury Salemi, Camila Rocon, Márcio Silva Miguel Lima, Silvio Henrique Barberato, Ana Clara Rodrigues, Arnaldo Rabschkowisky, Daniela do Carmo Rassi Frota, Eliza de Almeida Gripp, Rodrigo Bellio de Mattos Barretto, Sandra Marques e Silva, Sanderson Antonio Cauduro, Aurélio Carvalho Pinheiro, Salustiano Pereira de Araujo, Cintia Galhardo Tressino, Carlos Eduardo Suaide Silva, Claudia Gianini Monaco, Marcelo Goulart Paiva, Cláudio Henrique Fisher, Marco Stephan Lofrano Alves, Cláudia R. Pinheiro de Castro Grau, Maria Veronica Camara dos Santos, Isabel Cristina Britto Guimarães, Samira Saady Morhy, Gabriela Nunes Leal, Andressa Mussi Soares, Cecilia Beatriz Bittencourt Viana Cruz, Fabio Villaça Guimarães, Bruna Morhy Borges Leal Assunção, Rafael Modesto Fernandes, Roberto Magalhães Saraiva, Jeane Mike Tsutsui, Fábio Luis de Jesus Soares, Sandra Nívea dos Reis Saraiva Falcão, Viviane Tiemi Hotta, Anderson da Costa Armstrong, Daniel de Andrade Hygidio, Marcelo Haertel Miglioranza, Ana Cristina Camarozano, Marly Maria Uellendahl Lopes, Rodrigo Julio Cerci, Maria Eduarda Menezes de Siqueira, Jorge Andion Torreão, Carlos Eduardo Rochitte, Alex Felix

**Affiliations:** 1 Santa Casa de Misericórdia de Feira de Santana Feira de Santana BA Brasil Santa Casa de Misericórdia de Feira de Santana, Feira de Santana, BA – Brasil; 2 Universidade Federal da Paraíba João Pessoa PB Brasil Universidade Federal da Paraíba,João Pessoa, PB – Brasil; 3 Instituto do Coração Faculdade de Medicina Universidade de São Paulo São Paulo SP Brasil Instituto do Coração da Faculdade de Medicina da Universidade de São Paulo (Incor/FMUSP), São Paulo, SP – Brasil; 4 Faculdade Ciências Médicas de Minas Gerais Belo Horizonte MG Brasil Faculdade Ciências Médicas de Minas Gerais, Belo Horizonte, MG – Brasil; 5 Hospital Felicio Rocho Belo Horizonte MG Brasil Hospital Felicio Rocho, Belo Horizonte, MG – Brasil; 6 Escola de Ecografia de Pernambuco Recife PE Brasil Escola de Ecografia de Pernambuco (ECOPE), Recife, PE – Brasil; 7 Beneficência Portuguesa de São Paulo São Paulo SP Brasil Beneficência Portuguesa de São Paulo, São Paulo, SP – Brasil; 8 Instituto Dante Pazzanese de Cardiologia São Paulo SP Brasil Instituto Dante Pazzanese de Cardiologia, São Paulo, SP – Brasil; 9 DF Star, Rede D’Or São Luiz Brasília DF Brasil DF Star, Rede D’Or São Luiz, Brasília, DF – Brasil; 10 Hospital Israelita Albert Einstein São Paulo SP Brasil Hospital Israelita Albert Einstein, São Paulo, SP – Brasil; 11 Santa Casa de Feira de Santana Feira de Santana BA Brasil Santa Casa de Feira de Santana, Feira de Santana, BA – Brasil; 12 Instituto de Cardiologia e Transplantes do DF Brasília DF Brasil Instituto de Cardiologia e Transplantes do DF, Brasília, DF – Brasil; 13 Grupo Fleury Salvador BA Brasil Diagnoson, Grupo Fleury, Salvador, BA – Brasil; 14 Hospital da Bahia Salvador BA Brasil Hospital da Bahia, Salvador, BA – Brasil; 15 Escola Bahiana de Medicina e Saúde Publica Salvador BA Brasil Escola Bahiana de Medicina e Saúde Publica, Salvador, BA – Brasil; 16 Hospital do Coração São Paulo SP Brasil Hospital do Coração (HCor), São Paulo, SP – Brasil; 17 CardioEco Centro de Diagnóstico Cardiovascular e Ecocardiografia Curitiba PR Brasil CardioEco Centro de Diagnóstico Cardiovascular e Ecocardiografia, Curitiba, PR – Brasil; 18 UnitedHealth Group Rio de Janeiro RJ Brasil UnitedHealth Group, Rio de Janeiro, RJ – Brasil; 19 Faculdade de Medicina Universidade Federal de Goiás Goiânia GO Brasil Faculdade de Medicina da Universidade Federal de Goiás, Goiânia, GO – Brasil; 20 Hospital Pró-Cardiaco Rio de Janeiro RJ Brasil Hospital Pró-Cardiaco, Rio de Janeiro, RJ – Brasil; 21 Hospital Universitário Antônio Pedro Universidade Federal Fluminense Rio de Janeiro RJ Brasil Hospital Universitário Antônio Pedro da Universidade Federal Fluminense (UFF), Rio de Janeiro, RJ – Brasil; 22 Secretaria de Estado de Saúde do Distrito Federal Brasília DF Brasil Secretaria de Estado de Saúde do Distrito Federal, Brasília, DF – Brasil; 23 Clínica Cardio & Saúde Curitiba PR Brasil Clínica Cardio & Saúde, Curitiba, PR – Brasil; 24 Heart & Mind Care Serviços Médicos Manaus AM Brasil Heart & Mind Care Serviços Médicos (HMC), Manaus, AM – Brasil; 25 Clínica CNI Uberlândia MG Brasil Cardiologia Não Invasiva, Clínica CNI, Uberlândia, MG – Brasil; 26 Diagnósticos da América SA São Paulo SP Brasil Diagnósticos da América SA (DASA), São Paulo, SP – Brasil; 27 Hospital 9 de Julho São Paulo SP Brasil Hospital 9 de Julho, São Paulo, SP – Brasil; 28 Universidade Federal do Paraná Curitiba PR Brasil Universidade Federal do Paraná (UFPR), Curitiba, PR – Brasil; 29 Departamento de Cardiologia Pediátrica Sociedade Brasileira de Cardiologia São Paulo SP Brasil Departamento de Cardiologia Pediátrica (DCC/CP) da Sociedade Brasileira de Cardiologia (SBC), São Paulo, SP – Brasil; 30 Sociedade Brasileira de Oncologia Pediátrica São Paulo SP Brasil Sociedade Brasileira de Oncologia Pediátrica, São Paulo, SP – Brasil; 31 Universidade Federal da Bahia Salvador BA Brasil Universidade Federal da Bahia (UFBA), Salvador, BA – Brasil; 32 Instituto da Criança e do Adolescente Hospital das Clinicas Faculdade de Medicina Universidade de São Paulo São Paulo SP Brasil Instituto da Criança e do Adolescente do Hospital das Clinicas Faculdade de Medicina da Universidade de São Paulo (FMUSP), São Paulo, SP – Brasil; 33 Hospital Evangélico Cachoeiro de Itapemirim ES Brasil Hospital Evangélico, Cachoeiro de Itapemirim, ES – Brasil; 34 Instituto do Coração de Marília Marília SP Brasil Instituto do Coração de Marília (ICM), Marília, SP – Brasil; 35 Instituto do Câncer do Estado de São Paulo São Paulo SP Brasil Instituto do Câncer do Estado de São Paulo (ICESP), São Paulo, SP – Brasil; 36 Hospital Aliança Rede D’Or São Luiz Salvador BA Brasil Hospital Aliança Rede D’Or São Luiz, Salvador, BA – Brasil; 37 Fundação Oswaldo Cruz Rio de Janeiro RJ Brasil Fundação Oswaldo Cruz (FIOCRUZ), Rio de Janeiro, RJ – Brasil; 38 Grupo Fleury São Paulo SP Brasil Grupo Fleury, São Paulo, SP – Brasil; 39 Hospital Cardio Pulmonar Rede D’or São Luiz Salvador BA Brasil Hospital Cardio Pulmonar Rede D’or São Luiz, Salvador, BA – Brasil; 40 Universidade Federal do Ceará Fortaleza CE Brasil Universidade Federal do Ceará,Fortaleza, CE – Brasil; 41 Universidade Federal do Vale do São Francisco Petrolina PE Brasil Universidade Federal do Vale do São Francisco (UNIVASF), Petrolina, PE – Brasil; 42 Hospital Nossa Senhora da Conceição Tubarão SC Brasil Hospital Nossa Senhora da Conceição, Tubarão, SC – Brasil; 43 Universidade do Sul de Santa Catarina Tubarão SC Brasil Universidade do Sul de Santa Catarina (UNISUL), Tubarão, SC – Brasil; 44 EcoHaertel - Hospital Mae de Deus Porto Alegre RS Brasil EcoHaertel - Hospital Mae de Deus, Porto Alegre, RS – Brasil; 45 Universidade Federal de Ciências da Saúde de Porto Alegre Porto Alegre RS Brasil Universidade Federal de Ciências da Saúde de Porto Alegre, Porto Alegre, RS – Brasil; 46 Universidade Federal de São Paulo São Paulo SP Brasil Universidade Federal de São Paulo (UNIFESP), São Paulo, SP – Brasil; 47 Quanta Diagnóstico por Imagem Curitiba PR Brasil Quanta Diagnóstico por Imagem, Curitiba, PR – Brasil; 48 Hospital Santa Izabel Salvador BA Brasil Hospital Santa Izabel, Salvador, BA – Brasil; 49 Santa Casa da Bahia Salvador BA Brasil Santa Casa da Bahia, Salvador, BA – Brasil; 50 Instituto Nacional de Cardiologia Rio de Janeiro RJ Brasil Instituto Nacional de Cardiologia (INC), Rio de Janeiro, RJ – Brasil

## Abstract

**Figura Central:**
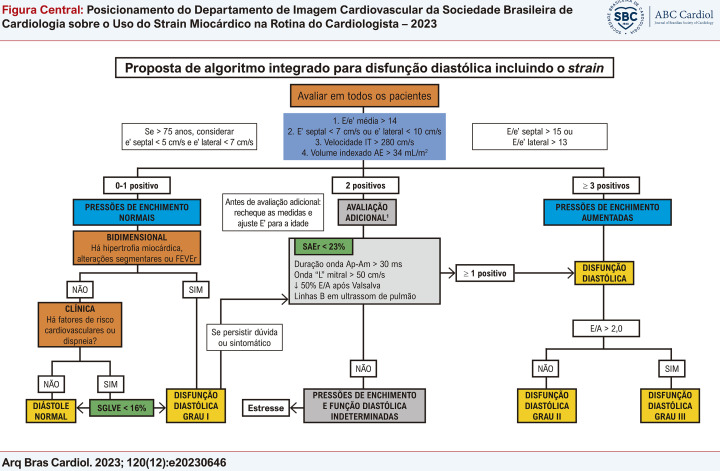
: Posicionamento do Departamento de Imagem Cardiovascular da Sociedade Brasileira de Cardiologia sobre o Uso do Strain Miocárdico na Rotina do Cardiologista – 2023

**Figura Central:** : Posicionamento do Departamento de Imagem Cardiovascular da Sociedade Brasileira de Cardiologia sobre o Uso do Strain Miocárdico na Rotina do Cardiologista – 2023

*Proposta de inclusão do strain no algoritmo integrado de avaliação da função diastólica, adaptado e traduzido de Nagueh et al.*^67^ *AE: átrio esquerdo; Ap: duração da onda A reversa pulmonar; Am: duração da onda A mitral; DD: disfunção diastólica; FEVEr: fração de ejeção do ventrículo esquerdo reduzida; IT: insuficiência tricúspide; SAEr: strain do AE de reservatório; SLGVE: strain longitudinal global do ventrículo esquerdo. Se remodelamento concêntrico, confirmar com SLGVE. Na presença de FEVEr, tempo de desaceleração da onda E mitral (TDE) < 160 ms e onda S < D pulmonar também são parâmetros de pressão de enchimento aumentada. Esse algoritmo não se aplica a pacientes com fibrilação atrial (FA), calcificação do anel mitral ou valvopatia mitral maior que discreta, bloqueio de ramo esquerdo (BRE), ritmo de marca-passo, próteses valvares ou hipertensão pulmonar (HP) primária grave.*

**Table:** 

Posicionamento do Departamento de Imagem Cardiovascular da Sociedade Brasileira de Cardiologia sobre o Uso do Strain Miocárdico na Rotina do Cardiologista – 2023	
O relatório abaixo lista as declarações de interesse conforme relatadas à SBC pelos especialistas durante o período de desenvolvimento deste posicionamento, 2022/2023.	
Especialista	Tipo de relacionamento com a indústria
Adenalva Lima de Souza Beck	Nada a ser declarado
Alex Felix	Nada a ser declarado
Ana Clara Rodrigues	Nada a ser declarado
Ana Cristina Camarozano	Nada a ser declarado
Anderson da Costa Armstrong	Declaração financeira A - Pagamento de qualquer espécie e desde que economicamente apreciáveis, feitos a (i) você, (ii) ao seu cônjuge/ companheiro ou a qualquer outro membro que resida com você, (iii) a qualquer pessoa jurídica em que qualquer destes seja controlador, sócio, acionista ou participante, de forma direta ou indireta, recebimento por palestras, aulas, atuação como proctor de treinamentos, remunerações, honorários pagos por participações em conselhos consultivos, de investigadores, ou outros comitês, etc. Provenientes da indústria farmacêutica, de órteses, próteses, equipamentos e implantes, brasileiras ou estrangeiras: - Amiloidose: Anylam. Outros relacionamentos Financiamento de atividades de educação médica continuada, incluindo viagens, hospedagens e inscrições para congressos e cursos, provenientes da indústria farmacêutica, de órteses, próteses, equipamentos e implantes, brasileiras ou estrangeiras: - Área da Saúde; CARDIOVASF.
André Luiz Cerqueira de Almeida	Declaração financeira A - Pagamento de qualquer espécie e desde que economicamente apreciáveis, feitos a (i) você, (ii) ao seu cônjuge/ companheiro ou a qualquer outro membro que resida com você, (iii) a qualquer pessoa jurídica em que qualquer destes seja controlador, sócio, acionista ou participante, de forma direta ou indireta, recebimento por palestras, aulas, atuação como proctor de treinamentos, remunerações, honorários pagos por participações em conselhos consultivos, de investigadores, ou outros comitês, etc. Provenientes da indústria farmacêutica, de órteses, próteses, equipamentos e implantes, brasileiras ou estrangeiras: - Boston Scientific: palestrante.
Andressa Mussi Soares	Declaração financeira A - Pagamento de qualquer espécie e desde que economicamente apreciáveis, feitos a (i) você, (ii) ao seu cônjuge/ companheiro ou a qualquer outro membro que resida com você, (iii) a qualquer pessoa jurídica em que qualquer destes seja controlador, sócio, acionista ou participante, de forma direta ou indireta, recebimento por palestras, aulas, atuação como proctor de treinamentos, remunerações, honorários pagos por participações em conselhos consultivos, de investigadores, ou outros comitês, etc. Provenientes da indústria farmacêutica, de órteses, próteses, equipamentos e implantes, brasileiras ou estrangeiras: - Bayer: Anticoagulação e insuficiência cardíaca; Pfizer: Anticoagulação e amiloidose; Jannsen: Leucemia. Outros relacionamentos Financiamento de atividades de educação médica continuada, incluindo viagens, hospedagens e inscrições para congressos e cursos, provenientes da indústria farmacêutica, de órteses, próteses, equipamentos e implantes, brasileiras ou estrangeiras: - Bayer: Insuficiência cardíaca.
Arnaldo Rabischoffsky	Nada a ser declarado
Aurélio Carvalho Pinheiro	Nada a ser declarado
Bruna Morhy Borges Leal Assunção	Nada a ser declarado
Camila Rocon	Nada a ser declarado
Carlos Eduardo Suaide Silva	Nada a ser declarado
Carlos Eduardo Rochitte	Nada a ser declarado
Cecilia Beatriz Bittencourt Viana Cruz	Nada a ser declarado
Cintia Galhardo Tressino	Nada a ser declarado
Claudia Gianini Monaco	Nada a ser declarado
Claudia R. Pinheiro de Castro Grau	Nada a ser declarado
Cláudio Henrique Fischer	Nada a ser declarado
Daniel de Andrade Hygidio	Nada a ser declarado
Daniela do Carmo Rassi Frota	Nada a ser declarado
David Costa de Souza Le Bihan	Declaração financeira A - Pagamento de qualquer espécie e desde que economicamente apreciáveis, feitos a (i) você, (ii) ao seu cônjuge/ companheiro ou a qualquer outro membro que resida com você, (iii) a qualquer pessoa jurídica em que qualquer destes seja controlador, sócio, acionista ou participante, de forma direta ou indireta, recebimento por palestras, aulas, atuação como proctor de treinamentos, remunerações, honorários pagos por participações em conselhos consultivos, de investigadores, ou outros comitês, etc. Provenientes da indústria farmacêutica, de órteses, próteses, equipamentos e implantes, brasileiras ou estrangeiras: - Edwards Lifescience; Abbott; GE Healthcare; Philips.
Eliza de Almeida Gripp	Nada a ser declarado
Fábio Luis de Jesus Soares	Nada a ser declarado
Fabio Villaça Guimarães Filho	Nada a ser declarado
Gabriela Nunes Leal	Nada a ser declarado
Henry Abensur	Nada a ser declarado
Isabel Cristina Britto Guimarães	Nada a ser declarado
Jeane Mike Tsutsui	Outros relacionamentos Participação societária de qualquer natureza e qualquer valor economicamente apreciável de empresas na área de saúde, de ensino ou em empresas concorrentes ou fornecedoras da SBC: - Área de saúde: Grupo Fleury.
Jorge Andion Torreão	Outros relacionamentos Participação societária de qualquer natureza e qualquer valor economicamente apreciável de empresas na área de saúde, de ensino ou em empresas concorrentes ou fornecedoras da SBC: - Sócio de empresa de educação na area da saúde.
Jorge Eduardo Assef	Nada a ser declarado
José Luiz Barros Pena	Nada a ser declarado
Jose Maria Del Castillo	Nada a ser declarado
Marcelo Dantas Tavares de Melo	Nada a ser declarado
Marcelo Goulart Paiva	Nada a ser declarado
Marcelo Haertel Miglioranza	Nada a ser declarado
Marcelo Luiz Campos Vieira	Nada a ser declarado
Márcio Silva Miguel Lima	Nada a ser declarado
Marco Stephan Lofrano Alves	Nada a ser declarado
Maria Eduarda Menezes de Siqueira	Nada a ser declarado
Maria Estefânia Bosco Otto	Nada a ser declarado
Maria Rosa Dantas	Nada a ser declarado
Maria Veronica Camara dos Santos	Nada a ser declarado
Marly Maria Uellendahl Lopes	Nada a ser declarado
Rafael Bonafim Piveta	Outros relacionamentos Participação societária de qualquer natureza e qualquer valor economicamente apreciável de empresas na área de saúde, de ensino ou em empresas concorrentes ou fornecedoras da SBC: - Sócio na WavesMed (plataforma digital de ensino continuado/atualizações).
Rafael Modesto Fernandes	Nada a ser declarado
Renato de Aguiar Hortegal	Nada a ser declarado
Roberto Magalhães Saraiva	Nada a ser declarado
Rodrigo Bellio de Mattos Barretto	Declaração financeira A - Pagamento de qualquer espécie e desde que economicamente apreciáveis, feitos a (i) você, (ii) ao seu cônjuge/ companheiro ou a qualquer outro membro que resida com você, (iii) a qualquer pessoa jurídica em que qualquer destes seja controlador, sócio, acionista ou participante, de forma direta ou indireta, recebimento por palestras, aulas, atuação como proctor de treinamentos, remunerações, honorários pagos por participações em conselhos consultivos, de investigadores, ou outros comitês, etc. Provenientes da indústria farmacêutica, de órteses, próteses, equipamentos e implantes, brasileiras ou estrangeiras: - GE; Abbot; Edwards.
Rodrigo Julio Cerci	Nada a ser declarado
Salustiano Pereira de Araujo	Nada a ser declarado
Samira Saady Morhy	Nada a ser declarado
Sanderson Antonio Cauduro	Nada a ser declarado
Sandra Marques e Silva	Outros relacionamentos Financiamento de atividades de educação médica continuada, incluindo viagens, hospedagens e inscrições para congressos e cursos, provenientes da indústria farmacêutica, de órteses, próteses, equipamentos e implantes, brasileiras ou estrangeiras: - Pfizer: Amiloidose; Sanofi, Pint Pharma, Takeda e Chiesi: Fabry.
Sandra Nívea dos Reis Saraiva Falcão	Nada a ser declarado
Silvio Henrique Barberato	Nada a ser declarado
Thais Harada Campos Espirito Santo	Nada a ser declarado
Tonnison de Oliveira Silva	Nada a ser declarado
Vera Maria Cury Salemi	Nada a ser declarado
Viviane Tiemi Hotta	Declaração financeira B - Financiamento de pesquisas sob sua responsabilidade direta/pessoal (direcionado ao departamento ou instituição) provenientes da indústria farmacêutica, de órteses, próteses, equipamentos e implantes, brasileiras ou estrangeiras: - Pfizer: Amiloidose.

## Sumário

1. Conceitos Básicos sobre o Estudo da Deformação do Ventrículo Esquerdo 7

1.1. Breve Introdução aos Princípios Físicos da Formação dos Speckles na Imagem Cardiovascular 7

1.2. Definições 7

**1.2.1.** ***Strain*** **e** ***Strain*** **Rate** 7

**1.2.2. Deformação Longitudinal, Circunferencial e Radial** 8

**1.2.3. Tempo dos Eventos Mecânicos** 8

**1.2.4. Medidas de Pico Extraídas das Curvas de Deformação** 8

1.3. Fatores que Afetam a Estimativa do *Strain* 8

**1.3.1 Qualidade da Imagem** 8

**1.3.2. Modalidade de Imagem Cardiovascular** 9

**1.3.3. Fabricante e Versão do Software** 9

**1.3.4. Condições Hemodinâmicas** 9

1.4. Strain Longitudinal Global 9

2. Recomendações Gerais para o Uso do *Strain*: Aplicabilidade Clínica, Comparação com a Fração de Ejeção e Descrição Adequada no Laudo 11

2.1. Valor Prognóstico, Padrões Paramétricos e Detecção Subclínica de Cardiopatias da Deformação Miocárdica 11

2.2. *Strain* ou Fração de Ejeção: Qual é a Melhor Alternativa? 11

2.3. Recomendações Gerais de como Reportar os Resultados do *Strain* e os Valores de Normalidade 11

2.4. Conclusão 11

3. *Strain* na Cardio-oncologia 14

4. *Strain* na Disfunção Diastólica 16

4.1. Introdução 16

4.2. *Strain* do Ventrículo Esquerdo 16

4.3. *Strain* do Átrio Esquerdo 16

4.4. Conclusão 16

5. *Strain* nas Cardiomiopatias 17

5.1. Introdução 17

5.2. Cardiomiopatia Dilatada 17

5.3. Cardiomiopatia Arritmogênica 17

5.4. Cardiomiopatia Hipertrófica 18

5.5. Endomiocardiofibrose 18

5.6. Miocárdio Não Compactado 18

6. *Strain* nas Valvopatias 19

7. *Strain* nas Cardiopatias Isquêmicas 20

7.1. Introdução 20

7.2. *Strain* na Síndrome Coronariana Aguda 20

7.3. *Strain* na Síndrome Coronariana Crônica 20

7.4. *Strain* do Ventrículo Direito na Cardiopatia Isquêmica 21

8. *Strain* nas Doenças Sistêmicas (Amiloidose e Doença de Fabry) 22

8.1. *Strain* na Amiloidose Cardíaca 22

**8.1.1. Papel da Análise da Deformação Miocárdica no Diagnóstico da Amiloidose Cardíaca** 22

8.2. Doença de Fabry 25

9. *Strain* na Hipertensão Arterial Sistêmica 26

9.1. Introdução 26

9.2. Hipertensão Arterial Sistêmica sem Critérios para Hipertrofia Ventricular Esquerda 26

9.3. Hipertensão Arterial Sistêmica com Critérios para Hipertrofia Ventricular Esquerda 27

9.4. Tratamento Clínico 27

9.5. Conclusão 27

10. *Strain* em Atletas 27

11. *Strain* na Ecocardiografia com Estresse 29

12. *Strain* nas Cardiopatias Congênitas 29

13. *Strain* do Ventrículo Direito 30

13.1. Introdução 30

13.2. Características Anatômicas e Funcionais do Ventrículo Direito 30

13.3. Ventrículo Direito e Parâmetros Ecocardiográficos na Avaliação da Função Sistólica 30

13.4. Aquisição e Limitações 31

13.5. Indicações/Valores de Normalidade 32

14. *Strain* do Átrio Esquerdo e do Átrio Direito 33

14.1. Técnica de Obtenção e Análise do *strain* do Átrio Esquerdo 33

14.2. Valores de Normalidade 33

14.3. Aplicabilidade Clínica do *Strain* do Átrio Esquerdo 33

**14.3.1. Insuficiência Cardíaca e Avaliação de Função Diastólica** 33

**14.3.2. Fibrilação Atrial** 34

**14.3.3. Valvopatias** 34

**14.3.4. Doença Arterial Coronariana** 34

14.4. *Strain* Atrial Direito 34

15. Avaliação da Torção do Ventrículo Esquerdo 34

15.1. Introdução 34

15.2. Definições e Nomenclaturas 35

15.3. Passo a Passo da Avaliação da Torção Ventricular pelo Ecocardiograma com Speckle Tracking 35

15.4. Aplicações Clínicas 35

16. *Strain* na Análise da Dissincronia Ventricular 36

16.1. Introdução 36

16.2. Avaliação da Dissincronia na Seleção dos Pacientes para a Terapia de Ressincronização Cardíaca 37

16.3. Avaliação de Viabilidade Miocárdica 37

16.4. Orientação do Local de Implante dos Eletrodos 38

16.5. Avaliação Prognóstica após a Terapia de Ressincronização Cardíaca 38

16.6. Ajuste nos Parâmetros de Ressincronização 38

17. Myocardial Work (Trabalho Miocárdico) 38

17.1. Introdução 38

17.2. Aquisição do Trabalho Miocárdico 38

17.3. Valores de Normalidade 39

17.4. Potencial Uso Clínico 42

18. *Strain* no 3D: O Que Pode Acrescentar ao Exame 43

18.1. Introdução 43

18.2. *Strain* Ventricular Esquerdo 43

18.3. *Strain* Ventricular Direito 43

**18.3.1. Aquisição e Análise do Full-volume 3D** 44

18.4. *Strain* Atrial Esquerdo 44

19. O papel da Ressonância e Tomografia Cardíacas na Avaliação do *Strain* 44

19.1. Introdução 44

19.2. Métodos de Aquisição do *Strain* pela Ressonância Magnética Cardíaca 44

19.3. *Strain* do Ventrículo Direito pela Ressonância Magnética Cardíaca 44

19.4. *Strain* do Ventrículo Esquerdo pela Ressonância Magnética Cardíaca 44

19.5. *Strain* do Átrio Esquerdo pela Ressonância Magnética Cardíaca 46

19.6. *Strain* pela Tomografia Cardíaca 46

Referências 46

## 1. Conceitos Básicos sobre o Estudo da Deformação do Ventrículo Esquerdo

### 1.1. Breve Introdução aos Princípios Físicos da Formação dos Speckles na Imagem Cardiovascular

A palavra “*speckle*” refere-se à aparência granular da imagem gerada por um sistema de imagem de coerência óptica, tal como o *laser*, tomografia de coerência óptica ou ultrassonografia.^1,2^

Na ecocardiografia, um pulso de ultrassom emitido propaga-se em linha reta, interagindo com as diferentes interfaces acústicas da cavidade torácica até atingir o coração. Entre os diversos fenômenos acústicos que ocorrem nesse percurso, parte do feixe de ultrassom emitido sofre reflexão pelas diferentes estruturas cardíacas, gerando um eco que é parcialmente captado de volta pelo transdutor e utilizado pelo *software* como entrada (*input*) para a elaboração das imagens de ecocardiografia. Nesse caso, o comprimento de onda do feixe ultrassonográfico é habitualmente menor do que o tamanho das estruturas refletoras.

Entretanto, quando o comprimento de onda é maior do que a microestrutura com a qual interage, há uma dispersão do feixe de ultrassom, que se irradia para todas as direções (dispersão difusiva ou “*diffusive scattering”*). Esse fenômeno é o resultado do padrão de interferência de todas as frentes de onda que sofreram dispersão a partir dos diferentes dispersores (diferenças locais de densidade e compressibilidade dos tecidos).

Parte da dispersão difusiva é capturada pelo transdutor, formando a imagem de aspecto granular que denominamos *speckle*.

A presença de *speckles* torna a imagem do modo B menos nítida para o operador humano, porém ela não deve ser vista como um ruído, pois traz consigo informações únicas de forma a atuar como uma “impressão digital” do meio estudado pelo ultrassom.^1^

### 1.2. Definições

#### 1.2.1. *Strain* e *Strain Rate*

*Strain* corresponde à quantidade deformação de um objeto em relação à sua forma original.^3^ Na cardiologia, esse conceito é representado como o percentual (%) de encurtamento/alongamento do coração em relação à sua medida inicial. Esse conceito pode ser aplicado para um segmento miocárdico (*strain* regional) ou para a totalidade de uma das câmaras do coração como o ventrículo esquerdo (VE) (*strain* global).

O *strain rate* indica a taxa de deformação miocárdica (%) a cada segundo(s^-1^) ou, em outras palavras, a velocidade com que a deformação ocorre.^3-4^

#### 1.2.2. Deformação Longitudinal, Circunferencial e Radial

A aplicação do conceito de deformação nos permite pormenorizar o estudo do encurtamento/alongamento do miocárdio do VE a partir de sua orientação em diferentes eixos.

**Figura 1.1:**
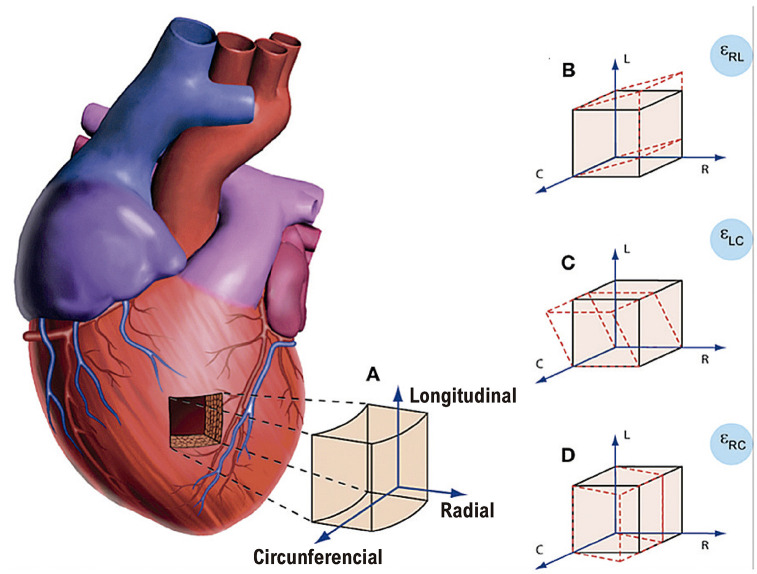
– A deformação miocárdica pormenorizada em diferentes eixos. A) A deformação básica pode ser aferida nos sentidos longitudinal, circunferencial e radial.4 A partir da interação de dois desses vetores-força, há o surgimento de um terceiro vetor resultante. B) Shear-strain radial-circunferencial. C) Shear-strain longitudinal-circunferencial (que equivale a torção ventricular/torsion). D) Shear-strain radial-circunferencial.

De fato, devido à disposição helicoidal das fibras musculares cardíacas, o encurtamento sistólico do VE é determinado pela ação de fibras no sentido longitudinal e de fibras no sentindo circunferencial,^5^ o que determina os dois vetores-força ativos da deformação (Figura 1.1 A).

A aplicação dessas forças no sentido longitudinal e circunferencial sobre um material de baixa compressibilidade (tecido miocárdico) resulta em um espessamento do miocárdio no sentindo radial (componente passivo da deformação).^6^ Em última análise, este responde pela diminuição radial da cavidade ventricular.^4^

É preciso ter em conta que o processo de deformação é bem mais complexo do que podemos aferir, pois, para cada processo de interação entre os vetores-força, surge um novo vetor resultante do cisalhamento entre as diferentes deformações, o *shear-strain* (Figura 1.1 B, C e D).

O encurtamento sistólico da fibra no sentido longitudinal e circunferencial produz valores negativos de *strain*. Já o espessamento sistólico radial atribui um valor positivo ao *strain*. Muitos autores optam por expressar apenas o valor absoluto (valor em módulo), e adotaremos essa abordagem aqui.

#### 1.2.3. Tempo dos Eventos Mecânicos

Descrevemos, a seguir, algumas definições fundamentais para a prática clínica:^1,7^

**• Final da sístole (*end-systole*):** definido como o ponto temporal de fechamento da valva aórtica. Potenciais substitutos: nadir do *strain* global ou da curva de volume. É recomendado que os *softwares* informem qual critério foi adotado para definir o final da sístole.
**• Final da diástole (*end-diastole*)**: definido como o ponto temporal no qual ocorre o pico do complexo QRS. A marcação de eventos (*event timing*) deve ser feita preferencialmente utilizando o Doppler e tendo como referência o eletrocardiograma (ECG).

#### 1.2.4. Medidas de Pico Extraídas das Curvas de Deformação (Figura 1.2)

***• Strain* do final da sístole *(end-systolic strain)*:** o ponto da curva de deformação no final da sístole, conforme previamente definido (fechamento da valva aórtica). Esse é o parâmetro padrão para descrever a deformação miocárdica.
***• Strain* de pico sistólico *(peak systolic strain)*:** o ponto onde ocorre o pico da curva durante toda a sístole.
***• Strain* de pico sistólico positivo *(positive peak systolic strain)*:** valor mais positivo registrado em casos em que a curva de um determinado segmento apresente esse comportamento em algum momento da sístole.
***• Strain de pico (peak strain)*:** o ponto onde ocorre o pico da curva de deformação, considerando todo o ciclo cardíaco. Habitualmente, esse ponto é alcançado até o fechamento da valva aórtica. Quando ocorre após, é descrito como *strain* pós-sistólico (*post systolic strain)*^8^ ou encurtamento pós-sistólico (EPS, *post-systolic shortening*). O *strain* pós-sistólico reflete a deformação de segmentos que se contraem após o fechamento da valva aórtica e não contribuem para a ejeção ventricular.

**Figura 1.2:**
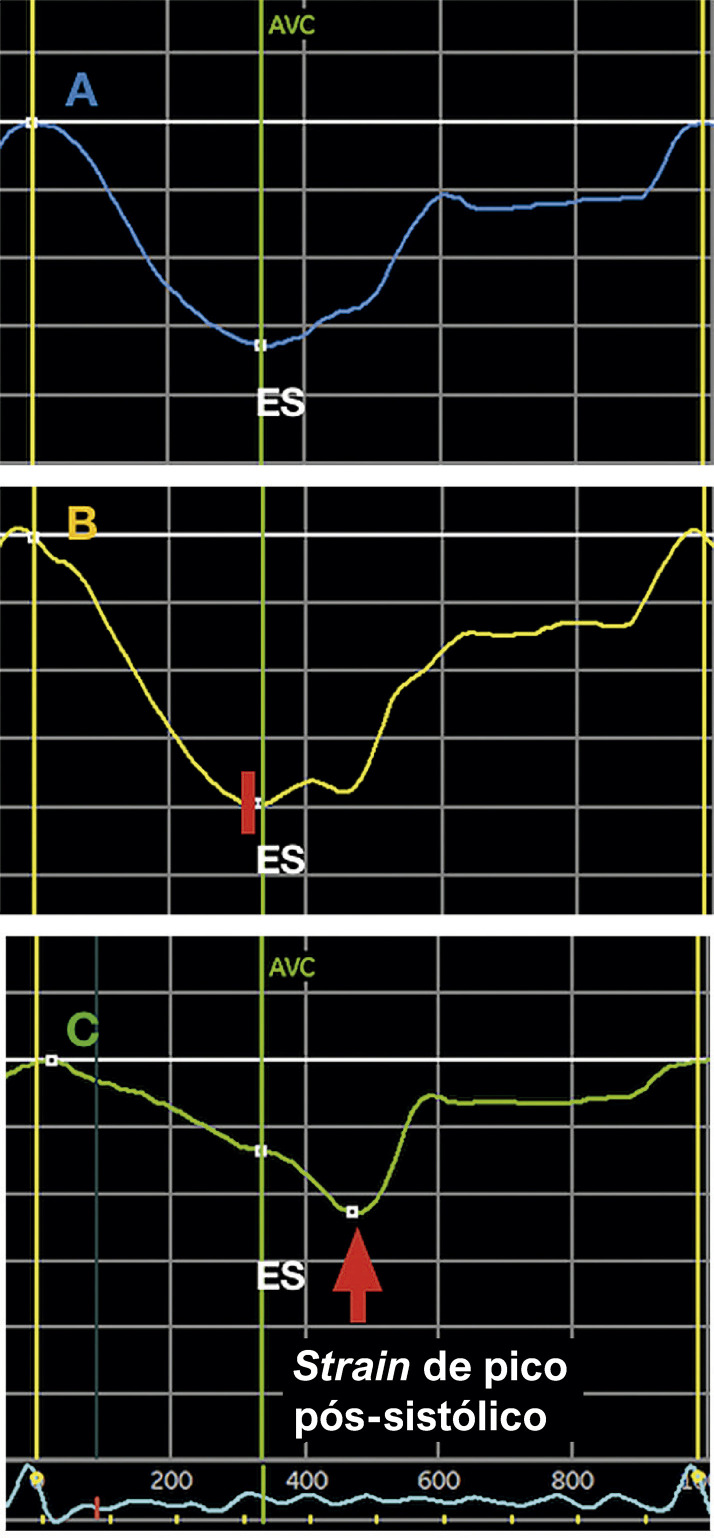
– Medidas de pico extraídas das curvas de deformação. A) O strain pico sistólico, o strain pico e strain do final da sístole (ES) coincidem no momento do fechamento da valva aórtica (AVC). B) O strain de pico sistólico e o strain de pico coincidem, porém ambos acontecem imediatamente antes do fechamento da valva aórtica (pequena barra vermelha), produzindo uma discreta dissociação entre aqueles e o strain do final da sístole (ES). C) O strain de pico sistólico e o strain do final da sístole (ES) coincidem (ambos apresentando valores absolutos reduzidos), porém o strain de pico ocorre após o fechamento da valva aórtica (fenômeno de encurtamento pós-sistólico).

## 1.3. Fatores que Afetam a Estimativa do *Strain*

### 1.3.1 Qualidade da Imagem

A qualidade da imagem é um fator crítico que afeta a *performance* de qualquer *software* que estime a deformação miocárdica. Vários autores reportaram a sensibilidade da estimativa do *strain* e *strain rate* proporcionais à qualidade da imagem e do algoritmo de *tracking.*^9-11^

### 1.3.2. Modalidade de Imagem Cardiovascular

Diferentes modalidades de imagem cardiovascular fornecem valores diferentes de *strain*. Tee et al.^12^ reportaram tais diferenças aferidas entre a ecocardiografia transtorácica, a tomografia computadorizada e a ressonância magnética cardíaca (RMC).

### 1.3.3. Fabricante e Versão do *Software*

Estudos organizados pela European Association of Cardiovascular Imaging (EACVI) e American Society of Echocardiography (ASE) testaram a variabilidade de medidas do *strain* longitudinal global (SLG) obtidas entre diferentes fabricantes de aparelhos e *softwares,* com evidência de divergências significativas.^13-14^ Contudo, há de se considerar que tais diferenças ainda são menores que a variabilidade da fração de ejeção (FE) reportadas na literatura.^9-10,15^

Além da variabilidade interfabricante, deve-se estar atento à variabilidade *intersoftwares* do mesmo fabricante. Mudanças significativas no SLG foram previamente reportadas.^11,15^

Dessa forma, estudos ecocardiográficos seriados deveriam, idealmente, ser realizados com o mesmo aparelho/*software* e sob condições hemodinâmicas semelhantes, sobretudo em situações cuja variação do SLG pode levar a implicações terapêuticas profundas, como no contexto de avaliação de cardiotoxicidade induzida por quimioterápicos, por exemplo.^4^

### 1.3.4. Condições Hemodinâmicas

A deformação do VE varia consideravelmente de acordo com as condições de pré-carga e pós-carga às quais o ventrículo está submetido.

## 1.4. *Strain* Longitudinal Global

É o parâmetro de deformação cardíaca com evidências científicas mais robustas e o único com uso de maior relevância na prática clínica.^9^ Ele reflete a deformação longitudinal relativa (%) do miocárdio do VE, que ocorre desde o período de contração isovolumétrica até o final do período de ejeção.^1,5,15^

Matematicamente, a contração em cada instante é computada pelo algoritmo como: SLG(t)=100[L(t)− L(ED)/L(ED)]  , em que L(t) é o comprimento longitudinal no tempo t, e L(ED) é o comprimento no fim da diástole.^1^

Há divergências significativas entre *softwares* quanto ao comprimento L(ED) utilizado: linha inteira da região de interesse (ROI) vs. média de determinado número de pontos do ROI x média dos valores em cada segmento do mesmo quadro.

O valor de normalidade do SLG é de, aproximadamente, 20%.^9^ Há evidências de variações dos valores de normalidade de acordo com sexo e idade.^7^

Para a análise do SLG do VE (SLGVE) por *speckle tracking*, é necessária uma série de cuidados relacionados à aquisição das imagens:

1) O paciente deve estar sob monitorização eletrocardiográfica.
2) Se possível, deve-se tentar apneia expiratória, evitando os movimentos de translação do coração com as incursões respiratórias.
3) Deve-se buscar um ponto de equilíbrio entre aspectos de resolução espacial e temporal do método ecocardiográfico, ponderando os ajustes do aparelho em relação ao foco, à profundidade e à largura, de modo que otimizem a câmara cardíaca de interesse, *versus* o *frame rate* (FR). Este último deve ser mantido entre 40 e 80 quadros por segundo (em pacientes com frequência cardíaca normal). É importante ratificar que, quanto maior a frequência cardíaca, valores mais altos de FR serão necessários.
4) Evitar “imagens truncadas” do VE (*foreshortening*).
5) Clipes das janelas acústicas apicais de 3, 4 e 2 câmaras devem ser adquiridos, preferencialmente com o mínimo de três batimentos, excluindo-se extrassístoles.

**Figura 1.3:**
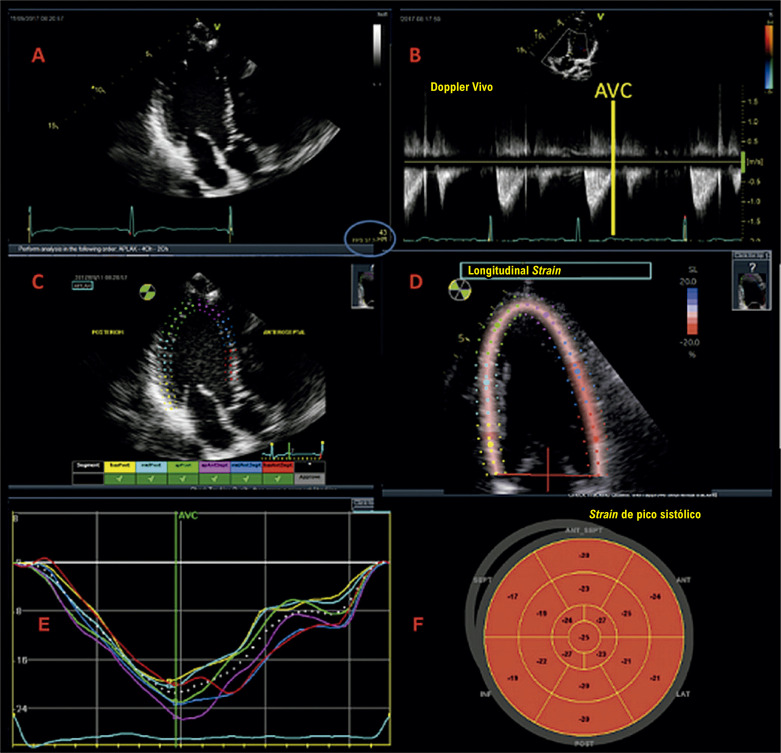
– Passo a passo para obtenção do strain longitudinal global. Inicialmente, adquirem-se imagens em 3, 4 e 2 câmaras, com eletrocardiograma de boa qualidade e com frame rate adequado (entre 40 e 80 quadros por segundo) (Imagem A, ovoide azul). Marca-se o fechamento da valva aórtica (AVC) a partir do traçado de Doppler pulsátil ou contínuo (Imagem B). Em seguida, faz-se a marcação de três pontos (dois na base e um no ápice), nas três imagens adquiridas, observando se o software faz um de rastreio adequado das imagens 2D (Imagens C e D). Finalmente, obtemos as curvas (Imagem E), o bull’s eye (Imagem F) e o valor obtido do strain longitudinal global. Adaptado de Tressino et al.4

A Tabela 1.1 e a Figura 1.3 trazem um resumo dos passos a serem seguidos para realizar a medida do SLG.

**Tabela 1.1:** – Passo a passo para medida do strain longitudinal global para a maioria dos fabricantes**4**

•Realizar monitorização eletrocardiográfica cardíaca com boa qualidade.
•Obter janelas acústicas de 3, 4 e 2 câmaras com frame rate entre 40 e 80 quadros por segundo.
•Marcar fechamento da valva aórtica (AVC).
•Marcar definições topográficas para delimitar a ROI nas janelas de 3, 4 e 2 câmaras.
•Aceitar ou descartar os segmentos miocárdicos rastreados em cada janela e fazer ajustes, se necessário.
•Avaliar as curvas e interpretar resultados obtidos no mapa polar.
•Registrar adequadamente as condições hemodinâmicas sob as quais o SLG foi aferido.

## 2. Recomendações Gerais para o Uso do *Strain*: Aplicabilidade Clínica, Comparação com a Fração de Ejeção e Descrição Adequada no Laudo

### 2.1. Valor Prognóstico, Padrões Paramétricos e Detecção Subclínica de Cardiopatias da Deformação Miocárdica

A análise de deformação miocárdica (*strain*) é uma ferramenta robusta e versátil que oferece informações adicionais e com menor variabilidade em relação aos parâmetros habituais sobre prognóstico, padrões paramétricos peculiares das cardiomiopatias (CMPs) e detecção de lesão subclínica.

Estudos recentes demostraram o valor incremental do SLGVE sobre a fração de ejeção do ventrículo esquerdo (FEVE).^10^ É importante realçar que a análise do *strain* apresenta uma variabilidade inter e intraobservador de 4,9 a 8,6%, bem menor que a FEVE, provavelmente por sofrer menos influência da pré- e pós-carga ventricular.^13,16^ Além disso, o SLGVE vem se tornando uma ferramenta superior à FEVE naqueles pacientes com insuficiência cardíaca com fração de ejeção reduzida (ICFEr) e preservada (ICFEp).^17,18^ Além da análise do VE, a piora do *strain* do ventrículo direito (VD) fornece valor aditivo prognóstico naqueles pacientes com ICFEp.^19^

**Figura 2.1:**
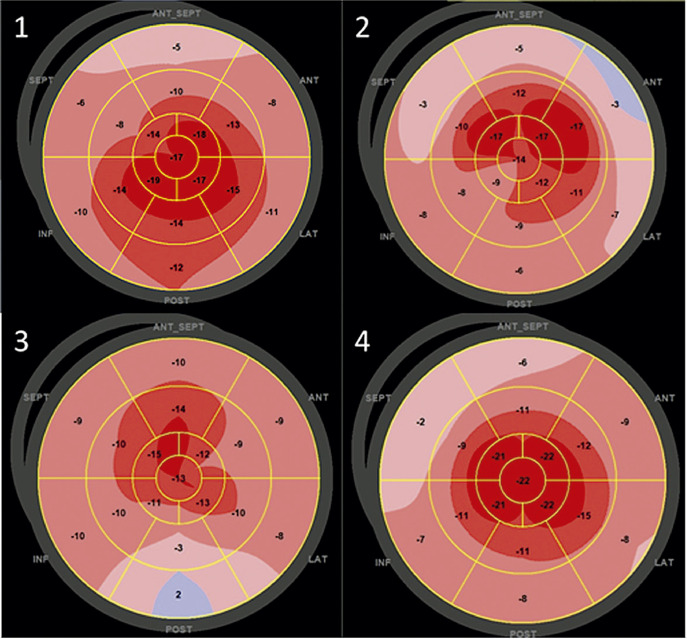
– Padrões de SLG com aspecto de poupar a ponta em diferentes cardiopatias. 1: Cardiotoxicidade por antracíclico; 2: Miocárdio não compactado; 3: Hipotireoidismo; 4: Amiloidose por transtirretina.

As CMPs compartilham achados morfológicos semelhantes na maioria das vezes, sendo um grande desafio diagnóstico na prática clínica diária. É comum haver a presença de aumento da massa e da espessura ventricular, associada à disfunção diastólica (DD) e com FEVE preservada nos estágios mais iniciais. A análise paramétrica do SLGVE pelo mapa polar possibilita que o exame de ecocardiograma desmascare alguns diagnósticos que não eram percebidos pelos parâmetros habituais, sendo descrito como uma “impressão digital” de algumas delas. O exemplo clássico é o padrão de poupar a ponta *(apical sparing)* da amiloidose, que será descrito com mais detalhes em capítulo específico.^20^ Essa caracterização fenotípica vem despertando muito entusiasmo por favorecer uma facilidade diagnóstica nas patologias raras. Por outro lado, é importante ressaltar que, se não combinarmos o *strain* com dados da história clínica, aspectos morfológicos e hemodinâmicos, favoreceremos o excesso e o erro diagnóstico. Veja esses exemplos de *“apical sparing”* (Figura 2.1).

A utilização do *strain* como ferramenta diagnóstica e prognóstica se consolidou através de sua aplicação na cardio-oncologia, quando há a oportunidade de ajustar a conduta terapêutica, baseada na variação se seu valor, em relação ao valor do exame basal durante a quimioterapia. Quando há uma redução relativa maior que 15%, considera-se cardiotoxicidade com lesão miocárdica subclínica.^21^ Em 2019, um posicionamento das diversas sociedades elaborou critérios para o uso adequado das diversas modalidades de imagens para avaliação das estruturas cardíacas nas doenças não valvares. Nesse documento, das 81 indicações descritas, apenas quatro consideraram o uso do *strain* adequado, sendo três na cardio-oncologia e uma para avaliação da CMP hipertrófica.^22^ Apesar da falta de estudos bem desenhados que validem essa ferramenta nas demais situações descritas por esse posicionamento, o *strain* é largamente utilizado nos grandes centros de cardio-oncologia. Recentemente, a atualização da Diretriz Brasileira de Cardio-Oncologia reforçou a sua utilização.^23^

### 2.2. *Strain* ou Fração de Ejeção: Qual é a Melhor Alternativa?

A FEVE é um dos principais parâmetros ecocardiográficos utilizados para a avaliação da função ventricular na prática diária, sendo um dado de fácil interpretação pelos clínicos, além de ser amplamente disponível e obtida em equipamentos básicos de ultrassom. Há extensa validação do uso desse parâmetro para o manejo de pacientes portadores de cardiopatias, sendo utilizado em grandes estudos de intervenção terapêutica como critério de inclusão de pacientes e, muitas vezes, servindo como parâmetro para a avaliação e o acompanhamento de resultados.^24^ O valor prognóstico da FEVE é bastante estabelecido na insuficiência cardíaca (IC) crônica^25^ e, por isso, na atual recomendação da Sociedade Europeia de Cardiologia, as ICs são classificadas com base no valor da FEVE em: **1**) IC com FE preservada (ICFEp: FE ≥ 50%); **2**) IC com FE em meio termo (ICFEmr: FE=40−49% ); e **3**) ICs com FE de ejeção reduzida (ICFEr: FE < 40%).^26^

A FEVE tem importante papel como parâmetro quantitativo para a definição de estratégias específicas em IC, por exemplo, servindo de critério para a indicação de terapia de ressincronização cardíaca em pacientes com IC refratária (FEVE ≤ 35%) ou mesmo na detecção de cardiotoxicidade em pacientes com câncer em uso de antracíclicos (queda evolutiva de FEVE ≥ 10% em relação ao basal com valor menor que o limite inferior da normalidade).^27^ No entanto, deve-se ressaltar que há limitação da acurácia da FEVE estimada pelo Simpson biplanar pela grande variabilidade interobservador dessa medida, que pode chegar até 13%.^28^ A ecocardiografia tridimensional (ECO3D), diferente do método Simpson biplanar, não se baseia em assunções geométricas e, por isso, mede diretamente os volumes das cavidades e a FEVE, com resultados bastante comparáveis aos obtidos pela RMC. Pelo uso de algoritmos automáticos e pela menor suscetibilidade a variações nas janelas de aquisição (orientação dos cortes apicais), a ECO3D possui menor variabilidade intra e interobservador que o método biplanar (0.4 ± 4.5%),^29^ sendo uma boa alternativa para o acompanhamento e a vigilância de pacientes com disfunção ventricular ou sob risco de dano miocárdico.

As técnicas de avaliação da deformação miocárdica, como o *strain*, permitem a avaliação dos três componentes de contração das fibras miocárdicas: longitudinal, radial e circunferencial. A FEVE é determinada sobretudo pelos componentes radial e circunferencial da contração miocárdica, que resultam no espessamento das paredes do miocárdio e na redução da cavidade ventricular na sístole. É importante notar, porém, que a FEVE não é um determinante único da *performance* ventricular (função “ejetiva”), sendo esta também dependente de um volume diastólico final (VDF) do VE adequado para gerar um volume sistólico normal. Isso explica por que, em pacientes com CMPs com expressão fenotípica de hipertrofia parietal concêntrica, como nas infiltrativas ou hipertróficas, podemos ter FEVE normal e baixo débito cardíaco. Esses pacientes se apresentam clinicamente como ICFEp e, à despeito de FEVE normal, têm geralmente pior prognóstico que pacientes com FEVE normal e débito cardíaco preservado, com alterações da função contrátil detectáveis apenas pelo SLG.^30^

De fato, a deformação longitudinal é o componente da contratilidade miocárdica que se altera mais precocemente em grande parte das CMPs, podendo sinalizar um processo em estágio inicial e subclínico (ainda sem redução da FEVE), fase de doença em que a instituição de medidas terapêuticas ou cardioprotetoras pode apresentar melhores resultados. O SLG pode se encontrar alterado até mesmo em doenças genéticas não fenotipicamente expressas, tal como em portadores de Ataxia de Friedreich com massa e FEVE normais, podendo, inclusive, predizer a queda da FEVE e o prognóstico nesses pacientes.^31^

Estudos demonstram o valor prognóstico adicional do SLG em pacientes com IC, com valor incremental ao efeito prognóstico da FEVE, sobretudo em pacientes com FE > 35%.^32^ Dessa forma, Potter et al. sugeriram uma nova classificação de função ventricular, incorporando à prática clínica o uso valores de SLGVE de forma complementar à quantificação da FEVE, auxiliando na decisão clínica e na avaliação prognóstica dos pacientes, sobretudo nos com FEVE > 53% (ICFEp).^10^

### 2.3. Recomendações Gerais de como Reportar os Resultados do *Strain* e os Valores de Normalidade

Com o objetivo de simplificar a descrição do *strain* no laudo de ecocardiograma, é recomendada a utilização do tipo de *strain* analisado (o qual define os movimentos de contração ou alongamento) e sua avaliação numérica em valores absolutos, principalmente em estudos comparativos sequenciais, com o objetivo de não levar a interpretações equivocadas de piora do *strain*. Outras informações cruciais que devem ser descritas são os sinais vitais do paciente (pressão arterial e frequência cardíaca), devido a alterações de pré- e pós-carga que influenciam o valor global do *strain*, a marca do equipamento de ultrassom utilizado, bem como a versão do *software* de análise, em decorrência da variabilidade da normalidade entre os fabricantes.^33,34^ A Tabela 2.1 abaixo descreve as informações essenciais que devem constar no laudo para descrição completa do *strain.*^9^

**Tabela 2.1:** – Elementos essenciais para a descrição do *strain* no laudo de ecocardiograma

Informações relevantes do *strain* no laudo	Descrição
Sinais Vitais	Pressão arterial e frequência cardíaca^16^
Tipo de *strain*	Longitudinal, circunferencial e radial
Valor absoluto do *strain*	Na descrição da função da câmara analisada
Câmara cardíaca analisada	VE, VD ou AE
Padrão de mapa polar (Figura 2.2)	Se há algum padrão típico, como em amiloidose ou cardiomiopatia hipertrófica^40^
Variação percentual em exames sequenciais	Utilização comprovada em cardio-oncologia. ∆%= *strain* exame basal, atual/basal^21^
Equipamento e versão do *software* utilizado	Há variabilidade do valor normal do *strain* segundo a marca e a versão do equipamento^37^

*∆%: variação (delta) percentual; AE: átrio esquerdo; VD: ventrículo direito; VE: ventrículo esquerdo.*

**Figura 2.2:**
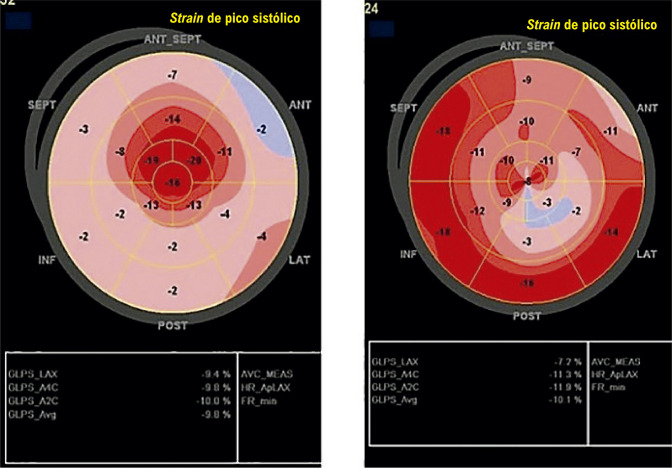
– Padrão de strain avaliado no mapa polar: A: Padrão típico de amiloidose (poupa o ápex); B: Padrão típico de cardiomiopatia hipertrófica com predomínio apical (strain reduzido predominante no ápex, em que a hipertrofia foi evidenciada como mais acentuada no estudo bidimensional).

Os valores normais de referência para o *strain* analisado^9,35-39^ devem ser incluídos no laudo. A Tabela 2.2 descreve valores médios da normalidade de forma simplificada dos diversos tipos de *strain*, bem como o grau de evidência para sua utilização na prática clínica. Diferentemente da FEVE, o valor de normalidade do *strain* ainda não foi assimilado de maneira consistente pelo cardiologista clínico e, portanto, devem constar no laudo como referência.

**Tabela 2.2:** – Valores de normalidade gerais para as diferentes modalidades de strain e câmaras cardíacas. Grau de comprovação da aplicação clínica

Câmara/Tipo de *strain*	Valor de normalidade (valor absoluto)	Aplicação na prática clínica
**VE** Longitudinal (negativo) Radial (positivo) Circunferencial (negativo)	18%^35,39^20%^35^ 40%^35^ 20%	++++ + +
**VD** Longitudinal parede livre (negativo)	20%^35,36^	+++
**AE** (varia com a idade, é positivo) Reservatório (mais utilizada) Conduto Bomba	39%^38,39^ 23%^38^ 17%^38,41^	+++ + +

*AE: átrio esquerdo; VD: ventrículo direito; VE: ventrículo esquerdo. ++++ Muito utilizado; +++ Utilizado; ++ Utilização limitada na prática clínica; + Utilização limitada na prática clínica, e não acessado em softwares embutidos nos equipamentos de ecocardio.*

### 2.4. Conclusão

A evidência atual é robusta para a incorporação do *strain* na prática clínica diária. Porém, ainda temos desafios para nossa realidade nacional, como a falta de democratização de acesso nos serviços de ecocardiograma com aparelhos com *softwares* para sua análise e falta de dados sobre a população brasileira. Utilizamos valores extrapolados de população com perfil sociodemográfico bem distinto de nossa realidade, com aplicação adaptada para a população brasileira. O Departamento de Imagem Cardiovascular está promovendo um trabalho multicêntrico (já em andamento), quando estão sendo analisados dados ecocardiográficos de brasileiros hígidos, para que possamos ter um retrospecto dos valores de normalidade em nossa população. O *strain* vem para se somar aos valores habituais do ecocardiograma, trazendo mais robustez prognóstica, possibilitando diagnóstico de CMPs, particularmente nas que se apresentam com aumento da espessura miocárdica, e, por último, diagnóstico de lesão miocárdica subclínica.

## 3. *Strain* na Cardio-oncologia

A disfunção cardíaca relacionada ao tratamento contra o câncer representa uma importante causa de morbidade e mortalidade nos pacientes oncológicos.^42,43^ Essa complicação pode interromper o tratamento e comprometer a cura ou o adequado controle do câncer.^44,45^Além disso, a IC relacionada à cardiotoxicidade por quimioterápicos frequentemente tem pior prognóstico que muitas neoplasias, com mortalidade de até 60% em 2 anos.^42^

A identificação precoce da cardiotoxicidade com a instituição de medidas cardioprotetoras tem potencial impacto prognóstico nesse cenário.^46,47^ Contudo, os métodos usualmente utilizados para esse diagnóstico, como a FEVE pela técnica bidimensional, têm baixa sensibilidade.^48,49^ Assim, a utilização de marcadores mais precoces para a identificação dessa complicação, como a análise do *strain*, tem grande destaque nesse contexto.

Os métodos de diagnóstico por imagem têm papel fundamental nesse cenário, e o ecocardiograma tem sido a ferramenta mais utilizada em função de sua correspondência anatômica, caráter não invasivo, fácil acesso, baixo custo e isenção de radiação ionizante.^27^ A FEVE é o parâmetro mais utilizado para o diagnóstico de cardiotoxicidade. Utilizando a técnica bidimensional, ela deve ser calculada pelo método de Simpson biplanar.^21^ A ECO3D, quando disponível, é a técnica de escolha para monitorar a FEVE em pacientes com câncer. Suas principais vantagens incluem maior acurácia no reconhecimento de FEVE abaixo do limite inferior da normalidade e maior reprodutibilidade que a técnica bidimensional, com acurácia semelhante à ressonância cardíaca. Entretanto, sua baixa disponibilidade, seu alto custo e a experiência do operador representam barreiras da técnica tridimensional.^27,50^

A disfunção ventricular relacionada ao tratamento contra o câncer é definida por queda absoluta da FEVE em mais de 10 pontos percentuais, para um valor inferior a 50%, na presença ou não de sintomas de IC. Recomenda-se que esse estudo ecocardiográfico seja repetido dentro de 2 a 3 semanas para se avaliar os efeitos da pré e pós-carga sobre a FEVE.

Apesar de ser um importante e já estabelecido fator prognóstico, a FEVE tem baixa sensibilidade para o diagnóstico de cardiotoxicidade, sendo dependente de alguns fatores como pré-carga cardíaca, qualidade da imagem e experiência do examinador. Além disso, ela pode subestimar o real dano cardíaco, uma vez que mecanismos hemodinâmicos compensatórios permitem o adequado desempenho sistólico do VE, mesmo na presença de disfunção dos miócitos.^48^ Assim, a redução da FEVE ocorre frequentemente em um momento muito tardio, quando, mesmo com a intervenção terapêutica, a maioria dos pacientes não tem recuperação funcional.^46,48,49^

Quando a detecção da cardiotoxicidade é precoce, com instituição de tratamento cardioprotetor, os pacientes têm maior potencial para a recuperação da função ventricular.^46,51^ Nesse cenário, o estudo da deformação miocárdica ou *strain* tem grande destaque. O *strain* calculado pela técnica de *speckle tracking* bidimensional (ST2D) tem surgido como um marcador sensível e reprodutível de análise da função sistólica e da contratilidade do VE, validado em modelos *in vitro* e *in vivo.*^52,53^ Tem sido crescente o número de publicações que demonstram a utilidade do estudo da deformação miocárdica pelo ST2D na detecção precoce e subclínica da cardiotoxicidade induzida por quimioterápicos, especialmente através da queda relativa do SLG.^23,54-57^

**Figura 3.1:**
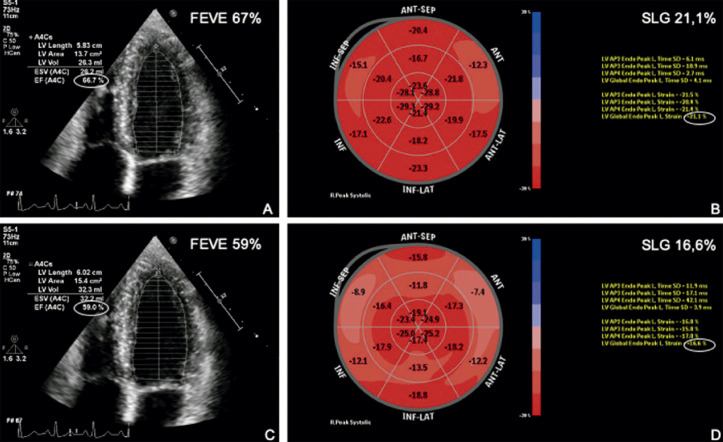
– Exemplo de cardiotoxicidade subclínica em paciente com câncer de mama. Análise da FEVE pelo método de Simpson e do SLG com imagem do bull’s eye. Em A e B, avaliação pré-quimioterapia; em B e C, avaliação após dose cumulativa de 240 mg/m2 de doxorrubicina. Não houve queda expressiva da FEVE, entretanto, foi observada queda relativa de 22% no SLG. FEVE: fração de ejeção do ventrículo esquerdo; SLG: strain longitudinal global.

É recomendada a análise do SLG nos pacientes que irão se submeter a tratamento quimioterápico potencialmente cardiotóxico. O diagnóstico subclínico de cardiotoxicidade é sugerido quando há uma queda do SLG maior ou igual a 12% em relação ao seu valor basal.^21,27^Na ausência de um estudo ecocardiográfico basal (pré-quimioterapia) para comparação, é sugerido, com base na opinião de especialistas, um valor absoluto de *strain* inferior a 17% como marcador de cardiotoxicidade subclínica, desde que não haja outros dados clínicos de sobreposição de outra doença miocárdica de base. Uma queda do SLG inferior a 8% do valor basal é considerada não significativa. A Figura 3.1 apresenta um exemplo de cardiotoxicidade subclínica sugerida pela queda relativa do SLG.

**Figura 3.2:**
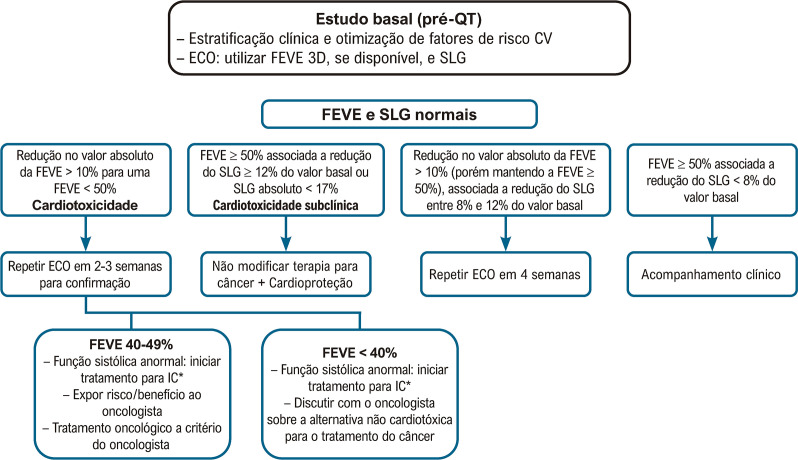
– Algoritmo de avaliação do paciente oncológico durante o tratamento quimioterápico, baseado na FEVE e SLG. CV: cardiovascular; ECA: enzima conversora da angiotensina; FEVE: fração de ejeção do ventrículo esquerdo; QT: quimioterapia; SLG: strain longitudinal global.

**Figura 3.3:**
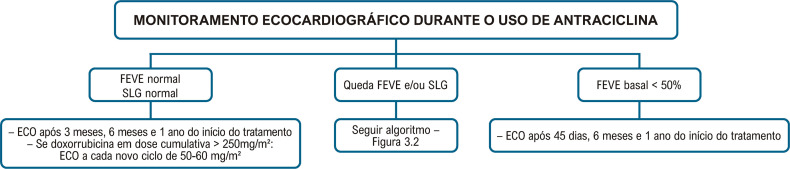
– Monitoramento ecocardiográfico durante o tratamento com antraciclinas. FEVE: fração de ejeção do ventrículo esquerdo; SLG: strain global longitudinal.

A Figura 3.2 apresenta o algoritmo do seguimento ecocardiográfico no paciente oncológico, baseado na FEVE e no SLG. As Figuras 3.3 e 3.4 apresentam o monitoramento ecocardiográfico em pacientes sob tratamento com antracíclicos e trastuzumabe, respectivamente.

**Figura 3.4:**
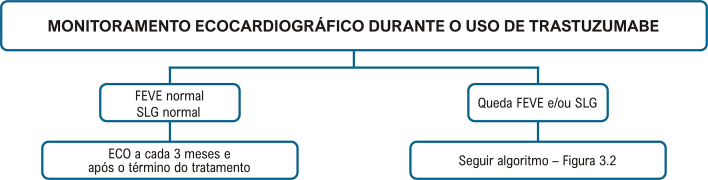
– Monitoramento ecocardiográfico durante o tratamento com trastuzumabe. FEVE: fração de ejeção do ventrículo esquerdo; SLG: strain global longitudinal.

O ensaio clínico randomizado SUCCOUR foi o primeiro estudo prospectivo e multicêntrico com maior poder científico que demonstrou o impacto prognóstico da cardioproteção guiada pelo SLG em comparação à cardioproteção guiada pela queda da FEVE pela ECO3D. Esse estudo revelou que, em pacientes recebendo quimioterapia com antracíclicos e com risco elevado de cardiotoxicidade, a cardioproteção (incluindo inibidor da enzima conversora de angiotensina [ECA] e betabloqueador) guiada por uma queda relativa do SLG maior ou igual a 12% do valor basal resultou em menor grau de queda da FEVE e menor incidência de disfunção cardíaca relacionada ao tratamento contra o câncer em 1 ano de seguimento.^58^

O *strain* calculado pelo *speckle tracking* tridimensional (ST3D) tem demonstrado vantagens técnicas em relação ao ST2D com acurácia, reprodutibilidade e aplicabilidade já demonstradas em diferentes cenários.^59-62^ Recentemente, pequenos estudos demonstraram o impacto do ST3D no reconhecimento precoce de alterações mecânicas relacionadas à quimioterapia.^63-66^ Entretanto, são necessários estudos maiores e principalmente com maior tempo de seguimento para avaliar o valor prognóstico dessa técnica.

Entre as limitações da análise do SLG, destacamos a variabilidade das medidas em equipamentos de diferentes fabricantes, de modo que as medições devem ser sempre feitas nos mesmos aparelhos. Assim como a FEVE, o SLG sofre influência pelos efeitos da pré- e pós-carga, geometria ventricular, alterações teciduais (infarto, miocardite, por exemplo) e distúrbios de condução. Por último, determinadas informações clínicas e oncológicas são fundamentais e devem ser reportadas no laudo para uma acurada interpretação ecocardiográfica, conforme apresentado na Tabela 3.1.

**Tabela 3.1:** – Informações clínicas e oncológicas relevantes no laudo ecocardiográfico

Tempo entre a infusão do quimioterápico e a realização do eco (pré ou pós)
Frequência cardíaca Pressão arterial Estado volêmico (descrição do diâmetro e variação da veia cava inferior)
Comparação com estudo basal, em especial FEVE e SLG – em caso de queda relativa, descrever o percentual desta redução
Aparelho/*software* utilizado para a análise do SLG

FEVE: fração de ejeção do ventrículo esquerdo; SLG: strain longitudinal global.

## 4. *Strain* na Disfunção Diastólica

### 4.1. Introdução

A DD é considerada um marcador precoce de dano miocárdico e, mesmo quando assintomática, pode determinar maiores taxas de mortalidade. Com a progressão da DD, ocorre aumento das pressões de enchimento do VE e ICFEp,^67,68^ sendo que esta última responde por mais de 50% das internações por IC e apresenta taxas de mortalidade equiparáveis às da ICFEr.^69^ Ao contrário da ICFEp, a DD pré-clínica é potencialmente reversível. Entretanto, a sua fisiopatologia é complexa e, apesar do uso integrado de vários parâmetros, o algoritmo atualmente recomendado é pouco sensível para detectar estágios subclínicos de DD.^70,71^

Casos indeterminados ainda são frequentes porque esses parâmetros nem sempre se alteram simultaneamente ou de forma linear.^70^ Fatores não diastólicos também podem contribuir para a ICFEp levando a expressões fenotípicas variadas, a depender do mecanismo fisiopatológico predominante.^72^ Ferramentas que avaliam as mecânicas ventricular e atrial esquerdas pela medida do *strain* podem suplantar esses desafios diagnósticos.^73,74^ O papel da mecânica ventricular direita nesse contexto ainda está sob investigação.^75^

### 4.2. *Strain* do Ventrículo Esquerdo

Diversos estudos têm demonstrado que parâmetros de deformação miocárdica avaliados por meio do *speckle tracking*, especialmente o SLGVE, têm melhor correlação com o relaxamento do VE e maior acurácia em predizer pressões de enchimento e intolerância ao exercício quando comparados a índices derivados do Doppler tecidual.^76-78^ A queda do SLGVE auxilia na detecção da DD em estágios mais iniciais e também prediz eventos cardiovasculares (CV) na ICFEp.^17,79-82^ Diante dessas evidências, o SLGVE reduzido (< 16%) já foi incluído como critério diagnóstico no novo algoritmo proposto pelas recentes diretrizes de ICFEp.^83^

### 4.3. *Strain* do Átrio Esquerdo

O *strain* atrial esquerdo (SAE) permite uma análise mais detalhada da função do átrio esquerdo (AE) e dos seus diversos componentes (reservatório, conduto e de bomba).^70^ Alterações no SAE expressam o acoplamento ventrículo-atrial e são resultantes da exposição crônica à elevação das pressões do VE, reduções da complacência e do relaxamento do AE,^41,84,85^ podendo preceder o seu remodelamento morfológico.^86-88^ Embora tenha sido descrita redução de todos componentes do SAE,^86,89,90^ o SAE de reservatório (SAEr) tem se mostrado o parâmetro mais robusto e se altera de forma linear com a progressão da DD.^91-93^ Morris et al., entre outros, demonstraram que o SAEr reduzido (< 23%) aumentou a detecção de DD, além de se correlacionar com pressões de enchimento e desfechos clínicos.^93-99^

Diante do número crescente de evidências, o SLGVE e o SAEr poderiam ser integrados ao algoritmo de DD vigente, conforme proposto na Figura Central. Essa estratégia pode ajudar a reclassificar os casos indeterminados e aumentar a acurácia para identificar estágios mais precoces de DD, especialmente em indivíduos com fatores de risco cardiovascular ou dispneia inexplicada.^97^

Consensos com padronização da metodologia do *strain* foram publicados para minimizar a variabilidade entre os fabricantes, o que ainda é uma limitação.^7,92,100,101^ Esperam-se novos estudos prospectivos e multicêntricos para avaliar se a modificação desses índices, com o tratamento, muda o prognóstico da DD e ICFEp.

### 4.4. Conclusão

O SLGVE e o SAEr são marcadores de doença subclínica que podem ser incorporados às recomendações vigentes para refinar o diagnóstico, o estadiamento e prognóstico da DD. Considerando-se a natureza complexa dessa avaliação, seria salutar a implementação e validação de algoritmos desenvolvidos por meio de inteligência artificial.

## 5. *Strain* nas Cardiomiopatias

### 5.1. Introdução

Seguindo um termo geral, as CMPs são afecções do músculo cardíaco. Em uma conotação mais pura e primária, elas não guardam uma associação com importantes causas sabidamente agressivas ao miocárdio, como a doença arterial coronariana (DAC), hipertensão arterial, valvopatias e cardiopatias congênitas. Podem ser divididas nos principais grandes grupos: dilatada, hipertrófica, restritiva, CMP arritmogênica e “miscelâneas não classificadas”.^102^

### 5.2. Cardiomiopatia Dilatada

Por definição, a CMP dilatada se caracteriza como uma doença que acomete o tecido miocárdico e que leva à progressiva redução da função sistólica e dilatação da cavidade do VE. Clinicamente, os indivíduos podem apresentar sinais e sintomas de IC, com necessidade de tratamento, hospitalizações, e por fim, transplante cardíaco.^102-107^

O ecocardiograma faz parte do arsenal diagnóstico de primeira-linha, com um papel extremamente importante no diagnóstico e prognóstico. Seus principais objetivos são a avaliação da dimensão volumétrica das câmaras cardíacas e a avaliação do desempenho sistólico do VE, classicamente realizada pela estimativa da FE, e que deve ser executada preferencialmente através do método de Simpson.

O *strain* é uma ferramenta ecocardiográfica adicional para agregar informação a essa avaliação e também possibilita detectar anormalidades sutis, subclínicas e em estágios iniciais de doenças.

**Figura 5.1:**
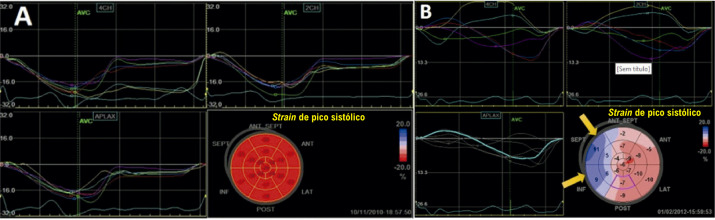
– A) exemplo de strain longitudinal global (SLG) normal do ventrículo esquerdo. Observam-se as deflexões negativas das curvas, que se apresentam relativamente homogêneas nas três incidências. Abaixo e à direita, a representação paramétrica em bull’s eye, com todos os campos em vermelho mais intenso, significando uma boa deformação global. B) exemplo de strain em um indivíduo portador de cardiomiopatia dilatada de etiologia chagásica. É possível observar a redução de amplitude das curvas, que aqui se apresentam também muito heterogêneas. As curvas de strain septal e na parede inferior têm deflexão positiva, ou seja, indicando distensão ou discinesia, o que está bem demonstrado mapa de bull´s eye em azul (setas amarelas), além do vermelho menos intenso, representando baixos valores de strain (SLG = -5,6%).

Abduch et al*.* demonstraram excelente correlação entre parâmetros volumétricos obtidos pela ECO3D e o *strain* em pacientes com CMP dilatada.^108^ Com a evolução da CMP dilatada para fases mais acentuadas de comprometimento sistólico do VE, haverá redução mais importante do *strain* e do *strain rate* nas três principais orientações (longitudinal, radial e circunferencial) (Figura 5.1).^109^ A torção do VE também acompanha essa tendência de diminuição com a progressão da doença. Adicionalmente, em fases muito avançadas, também pode haver inversão das rotações: segmentos basais com rotação anti-horária e os apicais em sentido horário.^110-112^

O SLG é um preditor independente de mortalidade por todas as causas em pacientes com ICFEr, especialmente em pacientes do sexo masculino sem FA.^113^ Já nos pacientes com FEVE recuperada, um SLG anormal prevê a probabilidade de diminuição da FEVE durante o acompanhamento, enquanto um SLG normal prevê a probabilidade de FEVE estável durante a recuperação.^114^

### 5.3. Cardiomiopatia Arritmogênica

A CMP arritmogênica é uma entidade histologicamente caracterizada por infiltração fibrogordurosa no tecido miocárdico. Essa infiltração ocorre preferencialmente nas vias de entrada, saída e ápice do VD, o chamado “triângulo da displasia”, entretanto o VE também pode ser acometido de forma concomitante ou até mesmo exclusiva.^115-117^

Macroscopicamente, a parede ventricular tende a se afilar, com formação de microaneurismas, e a tendência é progredir para comprometimento sistólico e dilatação da cavidade. O método diagnóstico padrão-ouro é a RMC, contudo o ecocardiograma é o exame inicial, e o *strain* da parede livre do VD pode auxiliar na determinação do comprometimento sistólico dessa cavidade.

Prakasa et al. foram os primeiros a analisar o *strain* na CMP arritmogênica com acometimento do VD, em 2007. Eles mostraram uma diferença consistente entre os valores do *strain* nos indivíduos doentes (10 ± 6%) em comparação com os normais (28 ± 11%, P = 0,001).^118^

O *strain* longitudinal da parede livre do VD (SL-PLVD) está associado à taxa de progressão estrutural em pacientes com CMP arritmogênica. Ele pode ser um marcador útil para determinar quais pacientes requerem acompanhamento e tratamento mais próximos. Pacientes com *strain* de VD menor que 20% tiveram um risco maior de progressão estrutural (*odds ratio*: 18,4; IC95% 2,7–125,8; P = 0,003).^119^

Pacientes com CMP arritmogênica apresentam redução do *strain* atrial direito (AD) em todas as fases da diástole, mesmo quando o volume do AD é normal. O *strain* do AD, obtido nas fases reservatório e bomba, está associado a um risco aumentado de eventos CV.^120^

### 5.4. Cardiomiopatia Hipertrófica

A CMP hipertrófica (CMPH) é uma doença autossômica dominante, sendo a doença cardíaca de etiologia genética mais comum. Caracteriza-se pelo aumento da espessura miocárdica ventricular de diferentes morfologias (podendo ser concêntrica, apical, septal, hipertrofia da parede livre do VE e do VD) e está relacionada ao aumento da morbimortalidade dos pacientes acometidos.^121-123^

O ecocardiograma é o método de imagem mais utilizado para o diagnóstico morfológico e hemodinâmico na CMPH. Cerca de 25% desses pacientes apresentam gradiente em via de saída do VE maior de 30 mmHg no repouso, que pode ser quantificado pelo Doppler contínuo.^124^ O gradiente dinâmico também pode estar presente nesses pacientes e pode ser mais bem avaliado pela realização das manobras de Valsalva durante o ecocardiograma, pelo ecocardiograma com estresse físico ou farmacológico com dobutamina.^125^ Níveis elevados de gradiente intraventricular na CMPH podem ser um dos determinantes para a queda da deformidade miocárdica e dos mecanismos de torção, assim como do *strain* no AE.^126^

**Figura 5.2:**
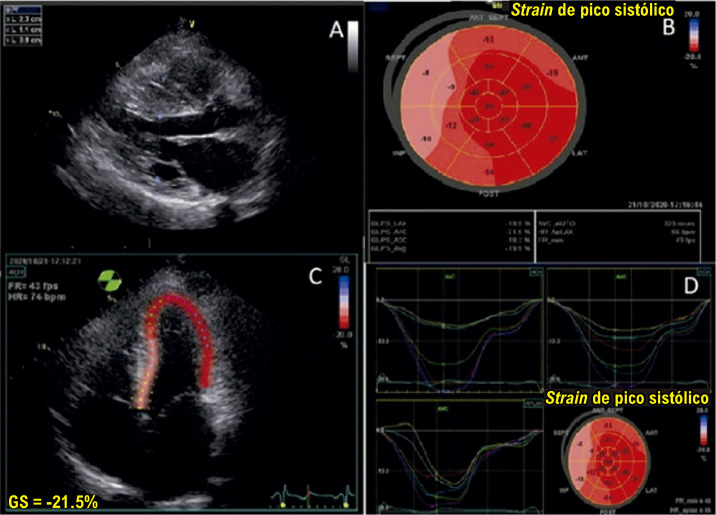
– A. Imagem bidimensional demonstrando hipertrofia septal assimétrica não obstrutiva, com septo interventricular medindo 23 mm e parede posterior de 11 mm. B. Strain longitudinal global normal do ventrículo esquerdo (20%), com representação paramétrica em bull’s eye, demonstrando segmentos com boa deformação miocárdica em vermelho mais intenso e menores valores de deformação nos segmentos septais, representados na cor rosa (8%). C. Incidência de 4 câmaras, com pior deformação miocárdica nos segmentos médio e basal ínfero-septal. D. Observa-se a redução de amplitude das curvas que representam os segmentos septais, também demonstrado mapa de bull´s eye em rosa, representando valores menores de strain (8%).

A técnica do *strain* miocárdico auxilia na análise da mecânica cardíaca regional e global na CMPH, sendo medida através do *speckle tracking*, e é capaz de detectar precocemente alterações da função sistólica, fibrose e até maior risco de o paciente desenvolver arritmia, mesmo nos casos de pacientes com função sistólica normal.^126-130^ O padrão do mapa polar auxilia na diferenciação das fenocópias que cursam com aumento da espessura, visto que a deformação miocárdica longitudinal apresenta-se reduzida no local da hipertrofia^20,131,132^ (Figura 5.2).

Hiemstra et al. identificaram que o volume atrial esquerdo indexado e o SLGVE são fatores prognósticos independentes de desfechos adversos como morte súbita e transplante cardíaco em pacientes com CMPH.^133^ Embora o SLG do VD (SLGVD) possa estar alterado no paciente com CMPH por ser uma doença estrutural cardíaca, seu significado prognóstico é desconhecido.^133,134^

### 5.5. Endomiocardiofibrose

A endomiocardiofibrose (EMF) é a CMP restritiva mais comum em nosso meio, na África equatorial e na Índia, afetando cerca de 10 milhões de pessoas no mundo. Ela é caracterizada pela deposição de tecido fibroso no endomiocárdio do ápice e da via de entrada de um ou ambos os ventrículos. A etiologia permanece desconhecida até hoje, podendo estar relacionada a hipereosinofilia, infestações parasitárias e desnutrição proteica, especialmente em populações de padrão socioeconômico comprometido.

O ecocardiograma mostra ventrículos de tamanho normal ou reduzidos, com morfologia ventricular em “cogumelo” ou em “V” pela deposição da fibrose, podendo estar associado à trombose endocárdica apical, hipermotilidade da região basal ventricular (sinal de Merlon), átrios de volume geralmente muito aumentados, função ventricular sistólica comumente preservada e DD.^135-137^

Poucos artigos avaliaram a EMF utilizando a ECO3D com *speckle tracking.* Esses estudos mostram redução do SLG, especialmente com comprometimento mais importante da região apical.^137,138^

### 5.6. Miocárdio Não Compactado

O miocárdio não compactado (MNC) é caracterizado pela presença de trabéculas proeminentes e espaços intertrabeculares profundos na cavidade do VE devido à compactação incompleta do miocárdio na vida embrionária. Isso pode levar a quadro clínico de IC, arritmias e eventos tromboembólicos. Existem duas formas, a esporádica e a familiar, sendo que a última está relacionada a mutações de proteínas do sarcômero. Os índices de deformação miocárdica permitem uma análise regional adequada da função ventricular em pacientes com MNC e auxiliam na diferenciação de outras CMPs.

Um estudo indiano comparou a deformação miocárdica de 12 pacientes com MNC, 18 pacientes com CMPH e 18 indivíduos saudáveis. Ambos os grupos de pacientes apresentaram redução do *strain* longitudinal, entretanto, os pacientes com MNC apresentaram maior redução do *strain* longitudinal na região apical quando comparados com o grupo de CMPH (12,18 ± 6,25 vs. 18,37 ± 3,67; p < 0,05), sugerindo um comprometimento maior dessa região na não compactação miocárdica. Além disso, um gradiente ápico-basal no *strain* longitudinal foi observado nos pacientes com MNC, mas não nos com CMPH.^139^ Ambos os grupos apresentam DD quando comparados com o grupo-controle. Outro estudo mostrou que o *strain* longitudinal é maior no grupo de MNC em relação à CMP dilatada idiopática e que o gradiente base-ápice do *strain* é um índice útil para diferenciar essas doenças com sensibilidade de 88,4% e especificidade de 66,7%.^140^

No coração normal, a base do VE gira no sentido horário durante a sístole, enquanto o ápice gira no sentido anti-horário, sendo que a torção é a diferença entre a rotação apical menos a basal. Um estudo prévio mostra que 50% dos pacientes com MNC apresentam rotação em corpo rígido (RCR), com rotação horária do ápice e da base; entretanto, estudos anteriores mostraram prevalência de 53,3% e 83%.^141,142^ Um estudo com 28 crianças com MNC mostrou que 39% dos pacientes apresentavam RCR, além de apresentarem menor *strain* longitudinal, mas não FEVE, em relação ao grupo sem RCR, podendo ter valor prognóstico.^143^ Além disso, os autores sugerem que o RCR está possivelmente relacionado principalmente à disfunção da camada apical subepicárdica compactada, sem relação com a distribuição de trabéculas. Outro estudo em 101 crianças com MNC mostrou que o grupo que apresentou desfecho adverso tinha redução do *strain* longitudinal, radial e circunferencial, sugerindo ser uma doença que afeta globalmente o coração e não apenas a região não compactada.^144^

## 6. *Strain* nas Valvopatias

O ecodopplercardiograma transtorácico é o método de primeira linha para o diagnóstico e a classificação da gravidade das valvopatias, por meio de uma análise combinada das alterações da anatomia e da função valvar.^145^ Esse método diagnóstico participa ativamente na definição do momento correto e do tipo de intervenção que deve ser realizada para o tratamento das valvopatias.

Classicamente, a indicação de tratamento é baseada na presença de sintomas ou de fatores complicadores.^145^ Entre os fatores complicadores, a disfunção ventricular esquerda é considerada o fator mais importante.^145^

A avaliação da função ventricular esquerda é habitualmente realizada pela ecocardiografia, por meio da medida da FEVE.^146^ Entretanto, várias evidências científicas têm demonstrado que a medida do *strain* ventricular esquerdo pode identificar a presença de disfunção ventricular antes da queda da FE.

A insuficiência mitral (IM), talvez seja a valvopatia que melhor representa esse paradoxo, uma vez que, nessa doença, o estado de alta pré-carga e baixa pós-carga faz com que a FE não represente adequadamente a função sistólica do VE. Por essa razão, as diretrizes clínicas são bastante conservadoras na definição de disfunção ventricular esquerda nessa condição.^145,147-149^ Entretanto, alguns estudos indicam que, mesmo utilizando esses parâmetros, o desfecho clínico após correção cirúrgica da IM pode não ser satisfatório, especialmente no que se refere à queda da FE e à presença de IC.^150-151^ Dessa forma, estudos têm demonstrado que mesmo em pacientes com FE acima de 60% e com diâmetro sistólico final do VE menor que 40 mm, a presença de SLG reduzido (≤ 19%) se associou à queda da FE abaixo de 50% no pós-operatório.^152-154^ Um SLG reduzido, abaixo de 18,1%, também se associou à maior mortalidade e a mais eventos CV em pacientes com IM seguidos prospectivamente e submetidos à cirurgia corretiva.^155^

Na insuficiência aórtica (IAo), demonstrou-se que a gravidade da valvopatia se correlaciona com a queda do *strain* ventricular esquerdo.^156^ Além disso, em pacientes com IAo importante e crônica, assintomáticos e com FE preservada, um SLG abaixo de 19% se associou à maior mortalidade ao longo do tempo, que era corrigida com a realização de troca valvar.^157^ O mesmo grupo mostrou que uma medida menor que 19% do SLG após a cirurgia, bem como uma queda de mais de 5 pontos percentuais no SLG, implicava em maior mortalidade.^158^

**Figura 6.1:**
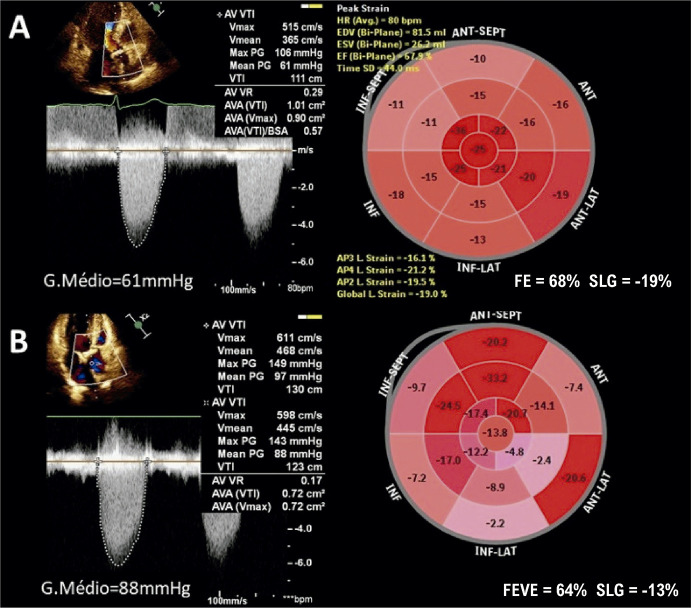
– Dois pacientes com estenose aórtica grave clássica, de alto fluxo, com fração de ejeção (FE) do ventrículo esquerdo normal, mas valores bastante distintos de strain longitudinal global (SLG). (A) Gradiente transvalvar aórtico médio = 61 mmHg, com FE e SLG normais. (B) Gradiente transvalvar aórtico médio = 88 mmHg, com FE normal e SLG diminuído.

Na estenose aórtica (EA) importante, a presença de FE menor que 50% e/ou de sintomas têm sido os pilares para indicação de tratamento.^145,147-149^ Entretanto, uma estratégia baseada em aguardar que a FEVE caia para < 50% para indicar a intervenção cirúrgica aórtica pode levar a desfechos clínicos insatisfatórios.^159^ Assim, o emprego de um parâmetro robusto de detecção de disfunção miocárdica subclínica, como o SLG, parece ser uma ferramenta de grande valor na estratificação do risco (Figura 6.1). Enquanto a FEVE não difere entre os graus de EA, o SLG diminui linearmente conforme a doença progride,^160^ acarretando maior risco de desfechos clínicos adversos, mesmo em assintomáticos.^161^

Diversos estudos examinaram o valor prognóstico do SLG para predizer a mortalidade e eventos CV em indivíduos assintomáticos com EA e FEVE preservada, visando selecionar quais devem ser encaminhados precocemente para a intervenção valvar.^162-164^ Os resultados desses estudos foram condensados em uma metanálise que definiu SLG < 14,7% como o valor de corte associado com maior risco de morte (sensibilidade de 60% e especificidade de 70%; área sob a curva [ASC] = 0,68).^165^ Foi encontrado SLG < 14,7% em aproximadamente um terço da população de indivíduos com EA moderada a grave e FEVE preservada, acarretando risco de morte 2,6 vezes maior. É importante ressaltar que a relação entre SLG e mortalidade foi significativa, tanto naqueles com FEVE entre 50-59% quanto naqueles com FEVE ≥ 60%. Em contraste, o achado de SLG > 18% se associou com excelente evolução clínica (97±1% de sobrevida em 2 anos). Portanto, o SLG diminuído, a despeito da FEVE preservada, configura-se como um poderoso preditor prognóstico a ser considerado na tomada de decisão clínica para indicar intervenção na EA grave assintomática, em conjunto com outros dados clínicos e ecocardiográficos.

## 7. *Strain* nas Cardiopatias Isquêmicas

### 7.1. Introdução

A ecocardiografia é uma excelente ferramenta a ser utilizada nas unidades de emergência para o diagnóstico de síndrome coronariana aguda e suas complicações. Ela oferece informações sobre o prognóstico desses pacientes a curto e a longo prazo, e o seu papel está bem definido na estratificação de risco da DAC estável e na avaliação de viabilidade miocárdica.

Entre as técnicas existentes, a ecocardiografia bidimensional com *strain* pelo *speckle tracking* (2DST) ratifica e acrescenta informações, sem estender em demasiado o tempo de exame. Ela avalia com boa acurácia a isquemia subendocárdica através do *strain* longitudinal em eventos agudos e crônicos.

Ao longo do texto, serão revisadas as indicações sobre o uso do *strain* longitudinal, circunferencial e radial nas cardiopatias isquêmicas, assim como outros dados fornecidos ao se calcular o *strain*, como a dispersão mecânica. Na Tabela 7.1 estão as principais indicações do *strain* na cardiopatia isquêmica

**Tabela 7.1:** – Principais aplicações do *strain* na cardiopatia isquêmica

Indicações do *strain* na cardiopatia isquêmica
Avaliação da alteração segmentar e global
Diferenciação de infartos subendocárdicos dos transmurais (acometimento longitudinal, radial e circunferencial)
Discriminar a artéria culpada de acordo com o acometimento pelo *bull’s eye*
Avaliação da melhora do *strain* global e segmentar após a revascularização miocárdica
Melhora a detecção de doença arterial coronariana
Preditor desfechos e de remodelamento após infarto agudo do miocárdio
Preditor de desfechos como hospitalizações por insuficiência cardíaca e mortalidade por todas as causas
Identifica pacientes com risco de arritmias

### 7.2. *Strain* na Síndrome Coronariana Aguda

O *strain* bidimensional é um marcador com boa sensibilidade para detectar isquemia miocárdica, considerado mais reprodutível que a FEVE e com acurácia confirmada pela RMC.^52,166^ A fibra subendocárdica é mais sensível à isquemia no seu estágio inicial, e o componente longitudinal predomina nesse tipo de isquemia.^167^ O SLG apresenta-se reduzido no infarto agudo do miocárdio (IAM) e correlaciona-se com a extensão do infarto, FE, eventos adversos e resposta às estratégias de reperfusão.^168-172^

Pacientes com infartos de pequena extensão, apresentam o SLG e radial reduzidos, enquanto o circunferencial e o *twist* se mantêm preservados. No entanto, o *strain* circunferencial estará comprometido também no infarto transmural.^173^

**Figura 7.1:**
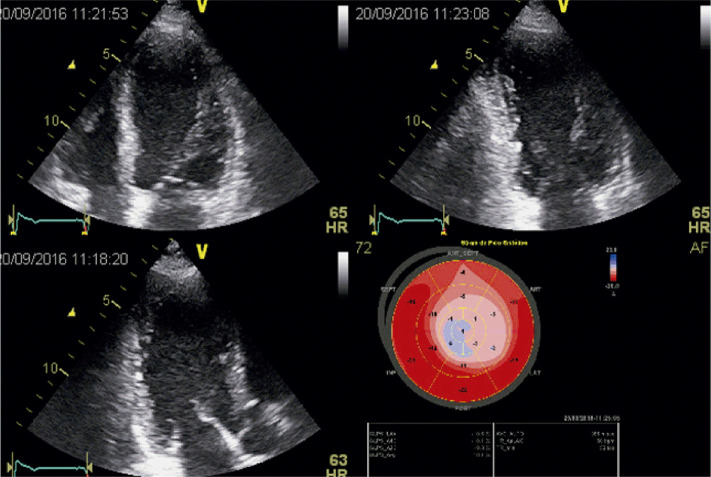
– Strain 2D visualizado através do bull’s eye demonstra alteração de deformidade na região apical do ventrículo esquerdo, com valores dos segmentos envolvidos reduzidos, compatível com lesão de artéria descendente anterior.

A identificação da extensão do infarto transmural apresenta uma implicação prognóstica importante, pois está associada a prognóstico reservado e a um maior número de eventos adversos. Os infartos subendocárdicos e não transmurais são associados com recuperação após revascularização (Figura 7.1).^174^

Um valor do *strain* longitudinal de 15% correlaciona-se com alterações segmentares (sensibilidade de 76% e especificidade de 95%).^168^O *strain* radial com o ponto de corte de 16,5% diferencia infartos transmurais dos não transmurais (sensibilidade de 70% e especificidade de 71,2%). O valor do *strain* circunferencial < 11% diferencia infarto transmural de não transmural (sensibilidade de 70% e especificidade de 71,2%).^175^Além disso, o *strain* longitudinal regional de 4,5% distingue infarto transmural de não transmural (sensibilidade de 81,2% e especificidade de 81,6%).^176,177^

Um outro dado importante em relação ao SLG é o seu valor diagnóstico na doença coronariana aguda (DCA) sem supra de ST para discriminar a artéria culpada. Uma coorte com 58 pacientes, dos quais 33 tinham DAC significativa (lesão acima de 50%) definida pela cineangiocoronariografia e submetidos à análise do *strain* antes do procedimento, demonstrou um ponto de corte de 19,7% (sensibilidade de 81% e especificidade de 88%, com ASC = 0,92) para detecção de DAC. O emprego de um ponto de corte de 21% foi capaz de excluir estenose coronariana significativa em 100% dos pacientes. O *strain* longitudinal territorial foi calculado como a média dos *strain* de pico sistólico dos segmentos que pertencem ao território daquele vaso estudado. Nesse trabalho, se o ponto de corte de 21% fosse aplicado, 16 pacientes seriam poupados da cineangiocoronariografia.^178,179^

O *strain* pode ser uma ferramenta para auxiliar na detecção de oclusão coronariana aguda em pacientes sem supra de ST, que podem se beneficiar de terapia de reperfusão precoce. Um estudo avaliou 150 pacientes, que realizaram o exame ecocardiográfico antes de serem encaminhados à cineangiocoronariografia. Desse total, 33 apresentaram oclusão coronariana aguda. Observou-se que um *strain* menor que 14% identificou a oclusão coronariana aguda (sensibilidade de 85% e especificidade de 70%), mas estudos mais robustos necessitam ser realizados para validação da técnica.^180^

O *strain* emergiu como uma nova técnica para detectar alterações subclínicas segmentares e globais, com *performance* acima de testes enzimáticos, ECG e escores de risco, além do seu papel na avaliação prognóstica desses pacientes com DCA. Trata-se de um exame rápido disponível à beira do leito e que pode ser realizado antes da cineangiocoronariografia, especialmente por ecocardiografistas treinados. Essa técnica está indicada, conforme os estudos citados, na DCA sem supra de ST para a avaliação das alterações segmentares e da função ventricular global, para diferenciar infartos pequenos dos infartos transmurais, discriminar a provável artéria culpada e para a avaliação pós-revascularização percutânea. Também é possível utilizá-la para a avaliação da viabilidade miocárdica após episódios de IAM.^181,182^

### 7.3. *Strain* na Síndrome Coronariana Crônica

A área mais suscetível a isquemia está localizada na região subendocárdica e, nessa localidade, as fibras estão orientadas no sentido longitudinal, portanto a avaliação da deformação longitudinal utilizando o 2DST seria um excelente marcador para a presença isquemia em comparação à avaliação somente pela ecocardiografia convencional.^183^

A interação do miocárdio normal e anormal gera padrões regionais típicos de deformação miocárdica, indicando que a contração miocárdica e a deformação miocárdica não são parâmetros intercambiáveis.^16,184^

O SLG pode ser muito mais sensível do que a FEVE na capacidade de detectar alterações precoces na isquemia miocárdica, pois avalia a função longitudinal do VE, porém ele não tem especificidade superior quando comparado às alterações da mobilidade de parede.^185,186^

A variabilidade das medidas regionais do *strain* pelo *speckle tracking* é relativamente alta, o que torna essas avaliações menos adequadas para o uso de rotina. No entanto, as medidas do SLG mostraram-se reprodutíveis e robustas, provavelmente devido à avaliação amplamente automatizada desse método.^187^ A outra alteração é a heterogeneidade regional de ativação miocárdica, que altera a sequência temporal de encurtamento e alongamento do miocárdio.

Na isquemia, não somente a amplitude do encurtamento é reduzida, como também o início e a duração da contração das fibras são alterados, o que gera um encurtamento ou espessamento característico do miocárdio após o fechamento da válvula aórtica.^187^ Essa alteração, chamada de encurtamento pós-sistólico (EPS), é característica do desenvolvimento de isquemia, apesar de poder ocorrer também na disfunção regional de qualquer causa (cicatriz, dissincronia etc.).^187,188^ Esse EPS pode ser entendido como um sinal de atraso do relaxamento para que a região isquêmica possa encurtar, enquanto a pressão do VE reduz e o tecido circundante relaxa.^16^ Um menor EPS com função sistólica normal é um achado frequente em 30% a 40% dos segmentos miocárdicos de corações saudáveis e pode ser encontrado principalmente no ápice e na base das paredes inferior, septal e antero-septal.^16,189^ Um dado importante no contexto da CMP isquêmica é a avaliação temporal do padrão das curvas do SLG, pois, muitas vezes, os segmentos isquêmicos podem ter valores do pico sistólico preservados, porém apresentam atraso temporal em relação aos outros segmentos não isquêmicos.

É importante ressaltar que as medidas do *strain* longitudinal regional não necessariamente refletem a impressão visual das alterações da contração, que é determinada pelo espessamento radial e pelo movimento endocárdico para o interior da cavidade.^16^

O SLG contribui para a detecção de DAC em pacientes com angina estável (estenose maior ou igual a 70%), com valores reduzidos na presença de DAC (17,1 ± 2,5% vs. 18,8 ± 2.6%; p < 0,001), especialmente quando associado ao teste ergométrico, e, além disso, identifica a provável artéria culpada.^183^ O emprego do *strain* longitudinal e, especialmente, do *strain rate* melhorou a sensibilidade e a acurácia na detecção das alterações segmentares no período tardio do pós-infarto do miocárdio.^189^

Na estratificação dos pacientes pós-IAM, um valor SLG menor que 15% antes da alta hospitalar foi um preditor independente de dilatação do VE em um seguimento de 3 a 6 meses, além de servir como um marcador do tamanho da área do infarto.^190^Nesse mesmo contexto de pós-IAM, um valor do SLG menor do que 14% foi um preditor independente de morte cardiovascular e internações por IC.^191^ Nos pacientes com doença crônica estável, um valor do SLG menor que 11,5% também demonstrou ser um preditor de morte por todas as causas e de desfechos combinados (morte por todas as causas e internação por IC).^192^

A heterogeneidade regional da contração miocárdica também pode ser avaliada pela dispersão mecânica, que é definida como o desvio padrão de tempo para atingir o pico tensão negativa entre todos os segmentos do VE. Esse índice tem um valor preditivo para taquiarritmia ventricular em pacientes pós-infarto. Foi demostrado que valores maiores de dispersão foram encontrados em pacientes que apresentaram arritmias no pós-IAM (85 +/- 29 ms vs. 56 +/-13 ms, p < 0,001).^193^

### 7.4. *Strain* do Ventrículo Direito na Cardiopatia Isquêmica

A função do VD é comprometida em aproximadamente um terço dos infartos de parede inferior e seu envolvimento tem sido descrito como um preditor importante de mortalidade hospitalar e de complicações maiores. A avaliação da função do VD é desafiadora devido à sua complexidade estrutural. O valor do *strain* da parede livre do VD demostrou ser um preditor de oclusão proximal da artéria coronária direita em pacientes com IAM de parede inferior (*strain* da parede livre do VD < 14,5%, ASC = 0,81; p < 0,001).^194^

Na doença isquêmica crônica estável, o *strain* da parede livre do VD apresenta-se alterado em pacientes com estenose da coronária direita (lesão maior que 50%) e pode ser usado para detectar disfunção subclínica nesse contexto.^195^

## 8. *Strain* nas Doenças Sistêmicas (Amiloidose e Doença de Fabry)

### 8.1. *Strain* na Amiloidose Cardíaca

A amiloidose é uma doença sistêmica causada pela deposição extracelular de fibrilas amiloides insolúveis nos tecidos. O acometimento cardíaco é um importante fator prognóstico e causa grande impacto na qualidade de vida dos pacientes, ocorrendo mais comumente nas formas causadas por cadeias leves (AL) e na amiloidose por transtirretina (ATTR).^196^

A ecocardiografia é um método de primeira linha para o diagnóstico e a avaliação prognóstica da amiloidose cardíaca (AC) e de outras doenças cardíacas infiltrativas. A maioria dos achados clássicos e sinais mais específicos da AC ao ecocardiograma ocorre somente em estágios muito avançados da doença.^30^ Situações clínicas como a IC com FE preservada e a presença de hipertrofia ventricular podem servir como sinais de alerta para a suspeição diagnóstica de AC.^197^

#### 8.1.1. Papel da Análise da Deformação Miocárdica no Diagnóstico da Amiloidose Cardíaca

**Figura 8.1:**
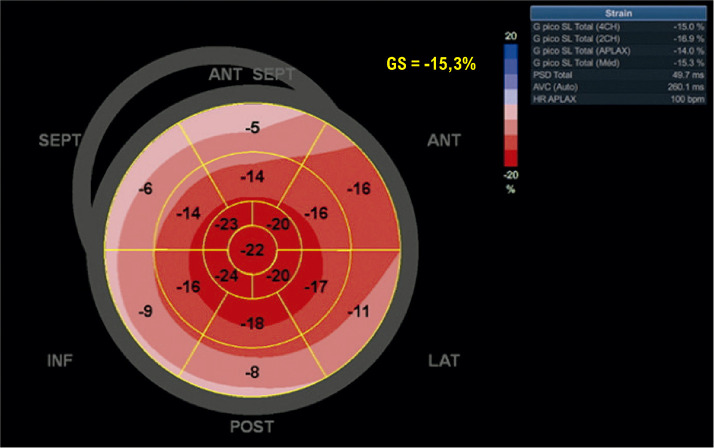
– Strain 2D longitudinal do ventrículo esquerdo em paciente com amiloidose cardíaca ATTR, demonstrando padrão de preservação relativa apical ou apical sparing, com maior acometimento da deformação segmentar em segmentos médios e basais, com maior deformação nos segmentos apicais.

O SLGVE encontra-se consistentemente alterado em pacientes com AC e está diretamente relacionado ao grau de infiltração amiloide, quantificado na ressonância magnética (RM) pelo grau de realce tardio por gadolíneo (LGE) e pelo volume extracelular (VEC) calculado em imagens de sequência T1.^30^ Um padrão de alteração regional dos valores de *strain* longitudinal (SL), com preservação relativa dos valores de deformação longitudinal dos segmentos apicais (RELAPS) foi descrita na literatura, definindo um gradiente basal-apical característico, conhecido como *apical sparing* ou *cherry on top* (Figura 8.1).

Na publicação original de Phelan et al., o RELAPS foi calculado a partir da seguinte equação: média do SL apical/(média do SL dos segmentos médios + média do SL dos segmentos basais), com valores > 1,0 apresentando boa acurácia para o diagnóstico de AC, com boa diferenciação de hipertrofias ventriculares causadas por EA e pela CMPH (ASC: 0,94).^20^

Esse padrão regional do SL, com gradiente basal-apical, é encontrado indistintamente nos tipos de amiloidose AL e ATTR. É importante enfatizar que o padrão clássico de *apical sparing*, embora descrito como característico para AC, pode estar ausente, conforme exemplificado no estudo de Ternacle et al., em que 52% dos pacientes com diagnóstico de AC tinham RELAPS “não diagnóstico” (< 1,0).^198^ Isso pode ser explicado, em alguns casos, pelo baixo grau de infiltração amiloide do miocárdio, em estágios bastante precoces da doença. Valores de SL regional septal apical/septal basal > 2,1 (SAB), quanto associados a tempo de desaceleração do influxo mitral < 200 ms, também demonstraram boa acurácia para a diferenciação de AC de outras doenças com fenótipo de hipertrofia parietal do VE, como doença de Fabry, ataxia de Friedreich, e hipertrofia do VE relacionada à hipertensão arterial sistêmica (HAS).^199^

A relação da FE do VE/SLG > 4,1 também foi demonstrada como um bom parâmetro para diferenciar AC de CMPH, com *performance* superior ao RELAPS ou SAB, independentemente do tipo de AC.^200^

**Figura 8.2:**
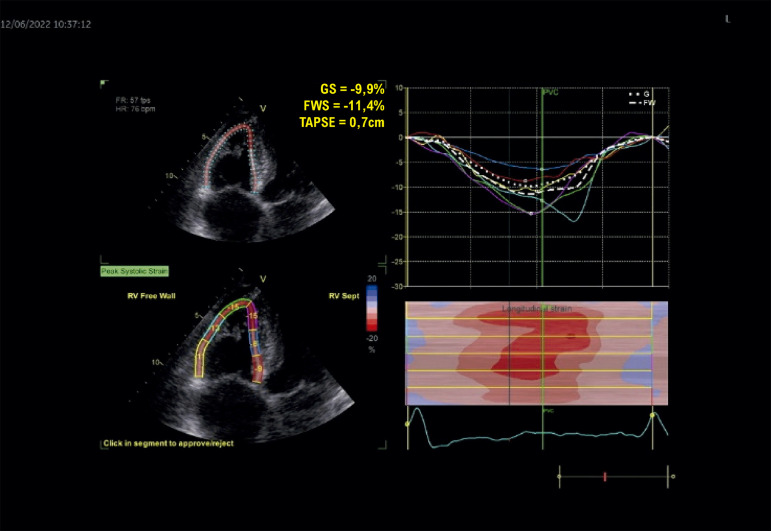
– Strain 2D longitudinal do ventrículo direito em paciente com amiloidose cardíaca ATTR, demonstrando redução do valor absoluto global (SLGVD = 9,9%) e redução absoluta do strain médio de parede livre (11,4%), com maior acometimento da deformação segmentar em segmentos médios e basais, com maior deformação nos segmentos apicais.

A deformação miocárdica do VD está geralmente reduzida em pacientes com AC, e seu achado pode ajudar a diferenciar de outras causas de hipertrofias parietais (Figura 8.2), tendo sido descrito também um padrão de preservação relativa apical, similar ao que é descrito no VE.^201^ Bellavia et al. demonstraram que alterações do VD podem ocorrer precocemente em pacientes com AC do tipo AL, mesmo em casos em que as espessuras parietais do VE ainda são normais.^202^

**Figura 8.3:**
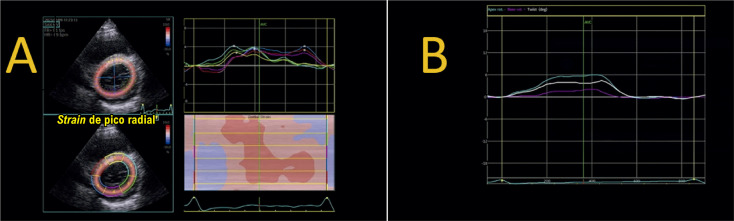
– Amiloidose ATTR. Strain 2D radial do ventrículo esquerdo (VE) na porção basal, demonstrando importante redução dos valores absolutos em todos os segmentos (A). Há também alteração do strain circunferencial, resultando em significativa redução do twist (4o) e torção do VE (B).

Na AC, assim como observado em outras CMPs infiltrativas, pode haver acometimento significativo de outros componentes da deformação miocárdica, como o *strain* circunferencial,^203^ *strain* radial,^204^ *twist* e torção (Figura 8.3). Em pacientes com amiloidose sistêmica em estágios iniciais de doença, sem evidência de AC, o *twist* e *untwist* podem estar aumentados de forma compensatória,^205^ sendo a deterioração desses parâmetros progressiva com o evoluir da doença,^206^ podendo levar, em casos avançados, à rotação da base e ápice cardíacos na mesma direção, criando um padrão chamado de *rigid body rotation*, com perda completa de importante contribuição da torção cardíaca à mecânica ventricular.

**Figura 8.4:**
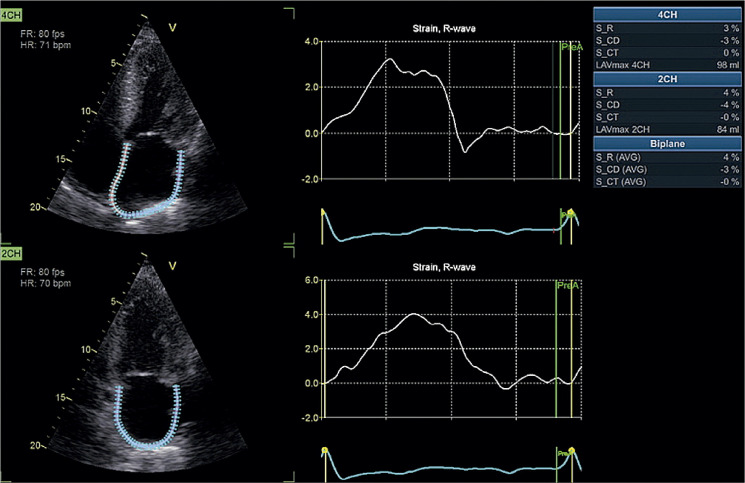
– Amiloidose AL. Strain bidimensional do átrio esquerdo (AE), com análise das janelas apical 4 câmaras e apical 2 câmaras demonstrando importante redução do valor do strain de AE biplanar (strain reservatório = 4%).

**Figura 8.5:**
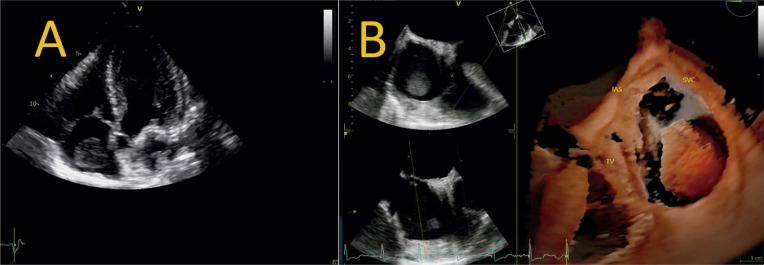
– Amiloidose ATTR. Na janela apical ao bidimensional (A), podemos observar a presença de grande massa móvel no interior do átrio direito em paciente com ritmo sinusal. Ao ecocardiograma transesofágico tridimensional (B), em imagem renderizada, observa-se a grande massa aderida ao apêndice atrial direito, correspondendo a trombo. IAS: septo interatrial; SVC: veia cava superior; TV: valva tricúspide.

O *strain* do AE também se encontra frequentemente alterado de forma significativa nos pacientes com AC, em parte como resultado da própria DD do VE, mas também de forma importante pelo efeito da infiltração direta da parede atrial por fibrilas amiloides (Figura 8.4). Em um estudo recente de Aimo et al., apenas o SL atrial de pico (LA-PALS) apresentou uma associação independente com o diagnóstico de AC, além das variáveis ecocardiográficas clássicas e dos biomarcadores cardíacos.^207^ Foi também reconhecido que a miopatia infiltrativa atrial avançada poderia causar grave disfunção e perda da eficiência mecânica da cavidade, levando a uma situação de “dissociação eletromecânica” atrial (DEMA).^208^ Em uma grande coorte de pacientes, Bandera et al. demonstraram a presença de DEMA (determinados pela análise do SL) em 22,1% dos pacientes em ritmo sinusal, que era fator determinante de mau prognóstico, quando comparado com a evolução de pacientes em ritmo sinusal e função mecânica atrial preservada.^209^ Em uma série de 156 pacientes com AC da Mayo Clinic, trombos intracardíacos foram detectados pelo ecocardiograma transesofágico em 27%,^210^ dados reproduzidos por outros estudos, com ocorrência de trombos inclusive em pacientes em ritmo sinusal^211,212^(Figura 8.5).

O *strain* 3D pode ser útil em demonstrar alteração de todos os componentes da deformação miocárdica em pacientes com AC. Vitarelli et al. demonstraram que a rotação basal de pico do VE, SL basal do VD e SL basal do VE foram capazes de distinguir com grande acurácia pacientes com AC de pacientes portadores de outras hipertrofias ventriculares.^213^ Em estudo de Baccouche et al.^214^ usando SL derivado da ECO3D, foi possível demonstrar o mesmo padrão de *apical sparing*, com gradiente basal-apical característico.

O trabalho miocárdico (TM), do inglês *myocardial work* (MW), também foi avaliado em pacientes com AC. Clemmensen et al. demonstraram que pacientes com AC tiveram menor índice de TM do VE (*left ventricular myocardial work index*, LVMWI) que o grupo-controle, com alterações mais pronunciadas nos segmentos basais e, quando submetidos a ecocardiograma de estresse, o aumento de LVMWI do repouso ao pico do exercício foi de 1.974 mmHg% em pacientes do grupo-controle (IC95% 1.699–2.250 mmHg%; p < 0,0001) comparado com apenas 496 mmHg% em pacientes com AC (IC95% 156–835 mmHg%; p < 0,01).^215^

O uso de *strain* para a avaliação evolutiva e para monitorar a resposta de pacientes em uso de tratamentos específicos para AC é bastante promissor. Giblin et al. avaliaram retrospectivamente 45 pacientes com AC ATTR em seguimento de 1 ano, comparando os valores de SL e MW entre os grupos de pacientes tratados e não tratados com Tafamidis.^216^ No grupo de pacientes não tratados, foi encontrada uma maior deterioração de SLG (p = 0,02), LVMWI e eficiência de TM (p = 0,04), sem diferenças significativas entre os grupos considerando os valores de *strain* circunferencial, *strain* radial, *twist* ou torção.

Os parâmetros de deformação miocárdica também foram bastante estudados como índices prognósticos na AC pela sua capacidade em fornecer dados quantitativos e pela sua alta sensibilidade e reprodutibilidade. Em estudo de Ternacle et al., foram preditores independentes de eventos cardiovascular maiores em um seguimento médio de 11 meses: SL apical médio (ponto de corte: -14,5%), NT-pro-BNP elevado e classe funcional NYHA III ou IV.^198^ Em outro estudo, o índice RELAPS se associou de forma independente com o desfecho composto de morte ou transplante cardíaco em 5 anos (*hazard ratio* 2,45; p = 0,003), mantendo o valor preditivo desse desfecho primário mesmo na análise multivariada (p = 0,018).^217^

Em um grande estudo publicado por Buss et al. incluindo 206 pacientes com AC AL, foi demonstrado que o SL baseado no Doppler e o SLG tiveram forte associação com os níveis de NT-proBNP e com a sobrevida (melhor ponto de corte: -11,78%), sendo que, na análise multivariada, apenas a DD e o SLG permaneceram como preditores independentes de sobrevida.^218^

Em trabalho recente, Liu et al. incluíram 40 pacientes portadores de mieloma múltiplo com FE preservada antes do início de tratamento com bortezomibe, medindo SLG e parâmetros de TM na linha de base.^219^ Os autores observaram que eficiência de TM global (*global MW efficiency*, GMWE) tinha associação significativa com eventos adversos cardíacos após 6 meses de quimioterapia, com ASC = 0,896 (IC95% 0,758–0,970; p < 0,05). O SL do VD também foi associado a prognóstico em pacientes com AC. Huntjens et al., estudando 136 pacientes com AC, demonstraram que valores de *strain* de todas as cavidades tinham associação significativa com sobrevida em um seguimento médio de 5 anos.^220^ O SL de pico do AE e o *strain* médio de parede livre do VD mantiveram associação independente com prognóstico na análise multivariada. Como variável independente, o *strain* de pico do AE teve a associação mais robusta com a sobrevida (p < 0,001), e combinando *strain* do AE com SLG e *strain* médio de parede livre do VD, foi obtida a maior capacidade de predição prognóstica (p < 0,001).

## 8.2. Doença de Fabry

A doença de Anderson-Fabry é a doença de depósito glicogênico mais comum, acometendo 1 a cada 50.000 indivíduos.^221^ É uma doença recessiva ligada ao X, portanto, afeta comumente mais indivíduos masculinos, sendo as mulheres carreadoras da mutação, e caracteriza-se pela ausência da atividade da alfa-galactosidade. Com isso, ocorre um acúmulo progressivo de globotriaosilceramida nos rins, coração e nervos. Clinicamente, os pacientes manifestam-se com alterações cutâneas (angioqueratomas), neuropatia periférica, insuficiência renal e IC decorrente de uma CMP restritiva com aumento da espessura miocárdica. Essas manifestações clínicas podem ocorrer na infância, porém, é mais comum que surjam após a terceira década de vida.^222^

A análise morfológica do acometimento ventricular apresenta a característica de aumento da espessura do VE, podendo evoluir para uma redução de sua complacência e IC com FE preservada por uma CMP restritiva. Outros achados interessantes que podem somar como *red flags* são a presença de hipertrofia do músculo papilar, sinal do duplo contorno do endocárdio e obstrução dinâmica na via de saída do VE.^223^ Esse fenótipo semelhante da CMPH é descrito em 6% dos homens^224^ e em 12% das mulheres, com o diagnóstico na faixa etária mais tardia.^225^ Por outro lado, a análise paramétrica disposta pelo *bull’s eye* da deformação longitudinal do VE tem um papel relevante na diferenciação das CMPs que cursam com aumento da espessura miocárdica, principalmente quando há um fenótipo assimétrico da hipertrofia ventricular esquerda (HVE), em que há a possibilidade etiológica de uma CMPH, amiloidose (principalmente por transtirretina), doença de Fabry e cardiopatia hipertensiva no idoso.

**Figura 8.6:**
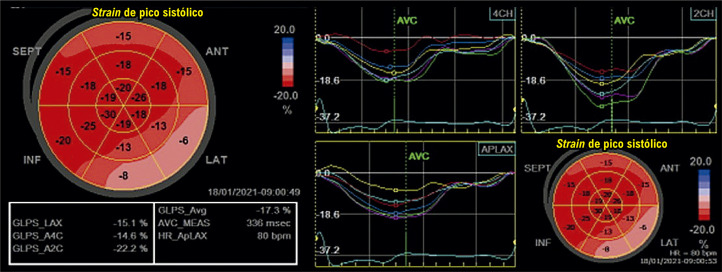
– Strain global longitudinal, em paciente com doença de Fabry, mostrando, no bull’s eye, os valores mais reduzidos nas porções basais da parede ínfero-lateral do ventrículo esquerdo.

Caracteristicamente, a CMPH apresenta os valores segmentares mais reduzidos nas porções em que ocorre maior espessura; a CMP amiloidótica ocorre um padrão de poupar o ápice e acometer mais as regiões médio-basais do VE, a cardiopatia hipertensiva pode se apresentar com discreta redução SLG. Porém, curiosamente, a doença de Fabry, apresenta um padrão singular em que, apesar do aumento assimétrico da espessura ventricular, a região mais acometida pela deformação longitudinal é a porção basal da parede ínfero-lateral (Figura 8.6), e ocorre um padrão decremental progressivo à medida que a doença evolui sem tratamento. Existe uma boa correlação entre o acometimento descrito pela análise de deformação longitudinal do VE e o realce tardio pela RM nas diferentes fases da doença.^222^ Além desse papel discriminatório, um estudo avaliou doentes com Fabry sem alterações morfológicas com um grupo-controle saudável. Nesse estudo, foi analisado o *strain* longitudinal do VE, VD e do AE com diferença entre os grupos (18,1 ± 4,0, 21,4 ± 4,9 e 29,7 ± 9,9 vs. 21,6 ± 2,2, 25,2 ± 4,0 e 44,8 ± 11,1%, p < 0,001). Interessantemente, além dessa diferença entre os grupos, as alterações da deformação apresentaram boa correlação com a gravidade dos sintomas.^226^

O tratamento já é disponibilizado, e é possível apresentar mudanças morfológicas do coração a partir de um ano de tratamento, como a redução de sua espessura.^227,228^ Sendo assim, é esperado que ocorra uma melhora da deformação longitudinal do VE mais precocemente, mesmo antes da redução da massa ventricular. Porém, a literatura ainda carece de publicações sobre a resposta terapêutica e o padrão do SLG ao longo do tratamento.

A análise da deformação miocárdica é uma importante ferramenta no diagnóstico das hipertrofias ventriculares sem etiologia, principalmente dentro de um contexto clínico coerente e com boa janela ecocardiográfica, acompanhamento dos familiares que não tiveram acesso ao estudo genético e avaliação de resposta terapêutica (Figura 8.6).

## 9. *Strain* na Hipertensão Arterial Sistêmica

### 9.1. Introdução

Nesta sessão, serão discutidas as principais vantagens e desvantagens do uso do *strain* em casos de HAS, com e sem critérios para CMP hipertensiva (presença ou não de HVE), e o seu valor clínico atual.

### 9.2. Hipertensão Arterial Sistêmica sem Critérios para Hipertrofia Ventricular Esquerda

**Figura 9.1:**
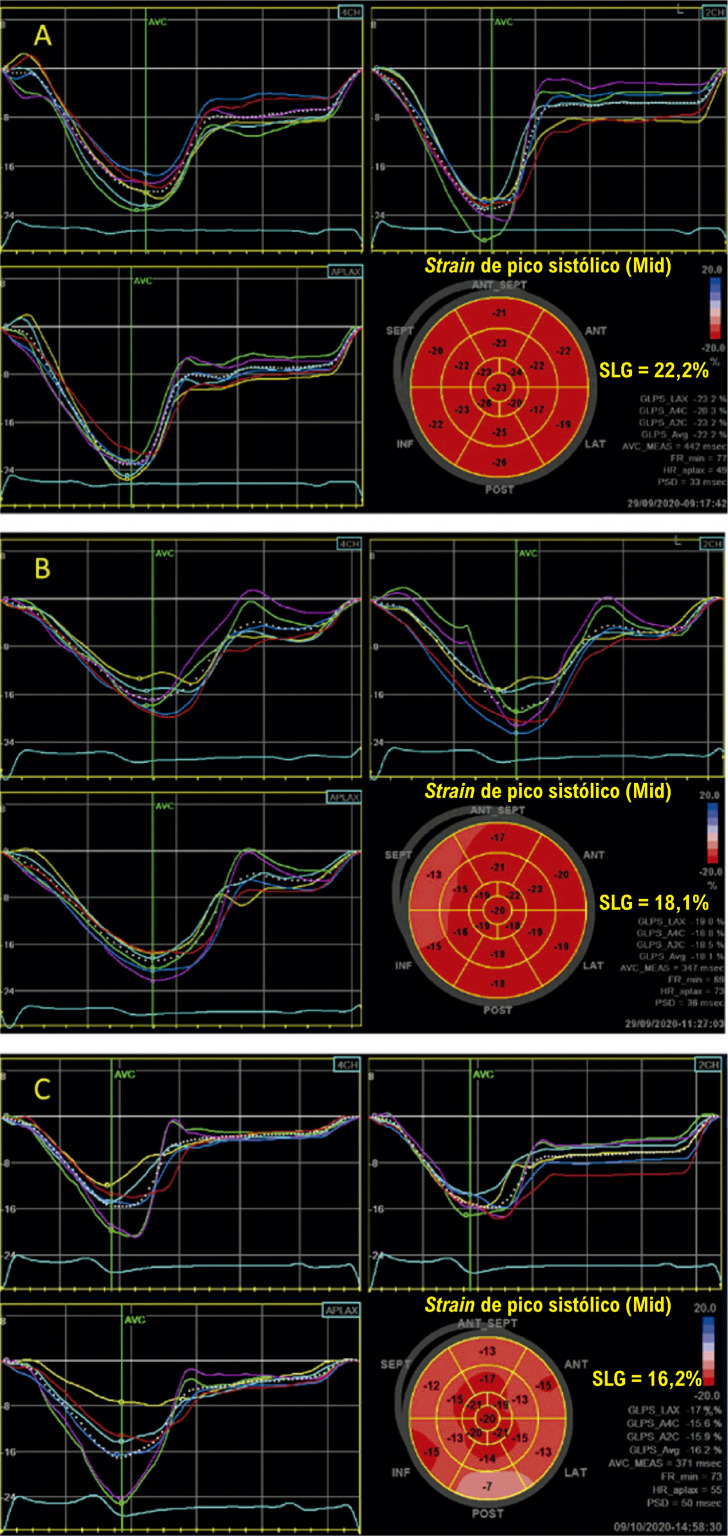
– Exemplos de mapa polar do strain longitudinal global (SLG) com as curvas do strain de pico sistólico obtidas em 4C, 2C e 3C. (A) Paciente saudável não hipertenso com SLG preservado; (B) Paciente hipertenso sem hipertrofia ventricular esquerda (HVE) com SLG no limite inferior da normalidade com alteração regional da deformação miocárdica em septo basal; (C) Paciente hipertenso com HVE com SLG reduzido e com maior alteração da deformação miocárdica nos segmentos basais e médios do que nos apicais.

A HAS provoca, ao longo de sua evolução clínica, alterações da contratilidade miocárdica comprovada com a redução do *strain* longitudinal em resposta à pós-carga e ao estresse sistólico de parede elevados, com significado prognóstico comprovado. A queda do SLG traduz a disfunção miocárdica subclínica antes mesmo do surgimento da HVE e queda de FE detectada pela medida tradicional, sendo essa medida do *strain* a única a se alterar em HAS estágio A do desenvolvimento de IC.^229-239^A redução do SLG acontece, inicialmente, na região basal do septo interventricular (SIV), estendendo-se para as regiões basal e média de outras paredes, e isso se deve à provável maior sobrecarga do SIV ao estresse sistólico de parede nos estágios iniciais da síndrome hipertensiva.^240,241^ (Figura 9.1) As fibras longitudinais da camada subendocárdica estão precocemente acometidas nessa fase inicial juntamente com o mesocárdio, ao contrário do epicárdio, como demonstrado em alguns estudos.^242^

Entretanto, a alteração do *strain* longitudinal da camada epicárdica foi a única variável preditora de eventos CVs em outra publicação, indicando que seu acometimento pode corresponder à lesão mais severa e crônica.^243^ Todavia, na grande maioria dos equipamentos disponíveis atualmente a análise por camadas do miocárdio não é possível. Por outro lado, os *strains* radial e circunferencial, que usam toda a espessura miocárdica em sua análise, têm uma tendência a permanecerem inalterados ou até mesmo aumentados como uma provável tentativa de compensação mecânica à redução do SLG^60,236^ e, quando o componente circunferencial se altera, pode traduzir disfunção miocárdica mais severa.^244^

As principais explicações para a redução do SLG estão associadas a um aumento da síntese de colágeno, culminando com a fibrose, marcador contundente de disfunção miocárdica. O SLG reduzido se correlaciona não somente com marcadores plasmáticos de fibrose, como a elevação do inibidor tecidual de metaloproteinase, mas também com a fibrose detectada com realce tardio pelo gadolíneo em estudos de RM em pacientes hipertensos.^231,234,238,239^ Reduções do SLG foram também observadas em pacientes com hipertensão dos tipos mascarada e “jaleco branco”^245,246^, com correlação dessas quedas com marcadores ecocardiográficos convencionais de DD^240^, além de maior deterioração a longo prazo em indivíduos que interromperam tratamento anti-hipertensivo.^247^

### 9.3. Hipertensão Arterial Sistêmica com Critérios para Hipertrofia Ventricular Esquerda

As consequências miocárdicas da doença hipertensiva crônica incluem a hipertrofia de miócitos, além de fibrose miocárdica e espessamento medial das artérias coronárias intramiocárdicas.^248^ Consequentemente, a HAS e as modificações do remodelamento miocárdico são fatores de risco para o desenvolvimento de eventos cardíacos maiores, tais como o risco de desenvolvimento de IC e morte prematura. Assim o uso do *strain* nesses casos tem o objetivo principal de detectar alterações sutis da função sistólica, antes mesmo do comprometimento da FE obtida de forma convencional, selecionando casos de ICFEP para a adoção de tratamento adequado.

Os tipos de remodelamento do VE podem apresentar alterações dos vários tipos de *strain*. Assim, na hipertrofia concêntrica, é possível encontrar valores reduzidos do SLG com queda progressiva de acordo com a evolução dos tipos geométricos, desde o remodelamento concêntrico até a hipertrofia excêntrica com dilatação do VE.^249-251^ O *strain* global circunferencial e o *strain* global radial encontram-se com valores preservados na maioria dos estudos^252^ ou até mesmo reduzidos em algumas séries^249^ e tendem a permanecer normais nas camadas epicárdicas em indivíduos com HAS e HVE.^250^ O comportamento da torção e do *twisting* também pode ser variável, com valores normais ou reduzidos de acordo com o tipo de geometria ventricular.^249,253^ Quando se considera o uso da técnica tridimensional do *strain*, o SLG 3D tende a se deteriorar de acordo com o grau de hipertrofia e diâmetro da cavidade do VE na HAS.^62,250^

Além da correlação do SLG reduzido com os diferentes padrões de HVE, o *strain* pode ser usado para auxiliar no esclarecimento da causa da hipertrofia e é frequentemente mais reduzido em casos de CMPH quando comparado com HVE por HAS.^254^

### 9.4. Tratamento Clínico

O SLG apresenta queda paralela com a piora da classe funcional^241^ e melhora com o tratamento a longo prazo, como demonstrado em uma análise de seguimento de 3 anos após tratamento anti-hipertensivo^255^ e em casos de tratamento anti-hipertensivo no ambiente de atendimento em emergência.^256^ O SLG reduzido também se correlacionou com a MAPA anormal em pacientes em tratamento, mesmo após ajuste de outras variáveis clínicas como idade, presença de diabetes melito e índice de massa do VE.^257^

### 9.5. Conclusão

Existem evidências suficientes para recomendar o uso do *strain* em pacientes com HAS, independentemente da presença de HVE, tanto para a identificação precoce das alterações estruturais subclínicas, como para os quadros de ICFEP visando a adoção de tratamento otimizado. Por outro lado, há a necessidade de estudos mais robustos para nortear o uso sistemático do *strain* nessa população.

## 10. *Strain* em Atletas

A atividade física regular e intensa é responsável por uma série de profundas alterações elétricas, estruturais e funcionais adaptativas, usualmente referidas como “coração de atleta”.^258^ A análise dessa condição é importante para uma melhor compreensão dos mecanismos de adequação cardíaca e melhoria da *performance* e do rendimento, orientando o treinamento otimizado. Além disso, permite a diferenciação de patologias que podem ter características morfológicas semelhantes àquelas induzidas pelo treinamento.

Atletas de alto rendimento e que apresentam grandes volumes do VE parecem pertencer ao espectro da fisiologia saudável típica do “coração de atleta”. Alguns trabalhos demonstram que o SLG se encontra discretamente reduzido nos atletas em repouso, quando comparado com sedentários; em outros, essa medida mostra-se superior aos controles.^259,260^ Entretanto, na maioria dos estudos, não foi identificada diferença significativa.^261^ Essa variação pode estar relacionada ao impacto de diferentes fatores como pré- e pós-carga, massa miocárdica e bradicardia sinusal. Por esse motivo, a presença de valores reduzidos de SLG em atletas com função diastólica do VE normal ou supranormal pode ser determinante para a distinção entre as adaptações secundárias aos exercícios e às patologias cardíacas. Valores absolutos iguais ou superiores a 18% são considerados ainda dentro da normalidade. A redução desses índices é muito mais acentuada em portadores de CMPH e HAS.^262^ No entanto, o *strain* circunferencial global e o *strain* radial não demonstraram alterações significativas em relação ao grupo-controle.^261^ A representação paramétrica em *bull’s-eye* pode fornecer subsídios para diferenciar o coração do atleta de outras doenças que cursam com hipertrofias.^263^

Quando os atletas são categorizados de acordo com o tipo e intensidade dos exercícios praticados em estático e dinâmico, surgem diferenças predominantemente em aspectos mecânicos do VE. Um estudo recente mostrou que a torção cardíaca foi maior em atletas com dinâmica baixa, estática alta (levantamento de peso, artes marciais), estática baixa e dinâmica alta (maratona, futebol) em relação aos controles. Contrariamente, a torção foi menor em atletas com dinâmica alta, estática moderada (natação, polo aquático), o que pode ser explicado por alterações na rotação apical, mas não na basal. O pico de *untwisting* foi maior em atletas com predominância de exercícios com componentes dinâmico baixo e estático alto, enquanto picos menores foram encontrados em atletas praticantes de esportes com componentes dinâmico alto e estático alto.^261^ Estudos utilizando o *speckle tacking* para quantificar a deformação miocárdica têm mostrado que atletas competitivos de resistência (*endurance*) apresentam valores normais ou aumentados do *strain*.^264-268^

Em relação ao VD, os índices de deformação miocárdica, obtidos tanto pelo Doppler tecidual quanto pelo *speckle tracking,* podem estar discretamente reduzidos nos segmentos basal e médio da parede livre do VD, notadamente em atletas de resistência em comparação aos controles.^269^ Ainda é controverso se tal redução da deformação miocárdica do VD é apenas uma resposta adaptativa ao exercício ou se é uma alteração subclínica por lesão miocárdica.^270^ Alguns autores supõem que tal achado pode ser explicado pelas mudanças na curvatura entre o ápice e base do VD, resultando nessa diferença do *strain* entre os segmentos.

Ainda são iniciais os estudos da função atrial em atletas com a técnica do *speckle tracking* e apresentam resultados conflitantes. Um estudo mostrou que a contração atrial avaliada pelo SLG do AE diminuiu significativamente após treino.^271^ Outro estudo não mostrou diferenças no *strain* atrial entre atletas e sedentários.^272^

A medida do *strain* também se faz importante na avaliação da função diastólica. O exercício dinâmico leva a um relaxamento ventricular mais efetivo, além da dilatação biventricular, entretanto, o exercício estático pode estar relacionado ao de aumento da espessura miocárdica e hipertrofia concêntrica do VE, podendo levar a algum grau de comprometimento da função diastólica.^273^ Além disso, o uso de drogas ilícitas para o aumento da *performance* pode levar à deterioração da função ventricular, sistólica ou diastólica; a ecocardiografia com *speckle tracking* pode detectar precocemente essas alterações.^274^

Por isso, é fundamental que, na avaliação da função ventricular dos atletas profissionais e/ou amadores, utilizemos todas as ferramentas disponíveis no arsenal da ecocardiografia. O *strain* é capaz de detectar alterações incipientes da função sistólica muito antes que ocorra qualquer alteração da contratilidade ao estudo bidimensional ou diminuição da FE.

A ecocardiografia com *speckle tracking* tem se mostrado bastante promissora para complementar a ecocardiografia bidimensional de rotina na avaliação de atletas. O SLG nessa população (diferentemente da população sedentária) pode ser considerado normal com valores absolutos superiores a 16%, valores inferiores devem levantar suspeita de patologia, principalmente se diante de outros sinais sugestivos como hipertrofia ou dilatação ventricular significativas.^275^

## 11. *Strain* na Ecocardiografia com Estresse

A Tabela 11.1 mostra as principais aplicações do *strain* na ecocardiografia com estresse.

**Tabela 11.1:** – Aplicações do strain na ecocardiografia com estresse

Cenário clínico	Conceito
Detecção de isquemia^189,276-280^	•SL regional detecta isquemia endocárdica. •SC é útil para a diferenciação entre infarto transmural e não transmural. •O aumento do tempo até o pico de SL é útil para a detecção precoce de isquemia. •Encurtamento pós-sistólico (sensível, mas não específico para detecção de isquemia). •SRL sistólico (menor dependência de carga e FC em relação ao *strain*). •Parâmetros de deformação diastólica.
Avaliação da viabilidade^281-285^	•SL e SRL aumentam a acurácia para a detecção de viabilidade em paciente com IM tratados com ATC em baixas doses de dobutamina. •SL é o melhor preditor de melhora da função após CRM. •Ausência de resposta à dobutamina prediz com acurácia a ausência de melhora após CRM. •Diferenciação entre miocárdio atordoado e hibernante (SL e SRL diminuídos e encurtamento pós-sistólico presente).
Estenose aórtica^286,287^	•SLG na avaliação da estenose aórtica baixo fluxo baixo gradiente. •Pacientes com SLG > 10%, após uso da dobutamina, apresentam maior sobrevida. •SLG de estresse apresentou maior acurácia do que ao repouso.
Miocardiopatia hipertensiva^288^	•Déficit de SL e SR mais evidente ao estresse do que ao repouso nas fases iniciais, possibilitando medidas de prevenção.
Miocardiopatia diabética^289,290^	•SRL diminuído nos segmentos médios e basais durante ESD •Velocidades miocárdicas diminuídas precocemente em pacientes assintomáticos com resistência à insulina durante ESD.
Cardiomiopatias^132,291-293^	•Diminuição da reserva funcional sistólica detectada ao SL e SR após estresse. •Ausência de melhora de parâmetros de função diastólica. *•Time-to-peak* aumentado na CMPH associado à dissincronia. •Baixos valores do SL do VD na displasia arritmogênica não melhora significativamente com estresse.
Coração de atleta^294,295^	•Valores do SL discretamente reduzidos com reserva miocárdica preservada ou supranormal. •O uso do SLG de estresse ajuda a diferenciar o coração de atleta de uma cardiomiopatia.

*ATC: angioplastia transluminal coronária; CMPH: cardiomiopatia hipertrófica; CRM: cirurgia de revascularização miocárdica; ESD: ecocardiografia de estresse com dobutamina; FC: frequência cardíaca; IM: infarto do miocárdio; SC: strain circunferencial; SL: strain longitudinal; SR: strain radial, SLG: strain longitudinal global; SRL: strain regional longitudinal; VD: ventrículo direito.*

Em breve, um artigo de revisão mais completo sobre ecocardiografia de estresse será publicado neste periódico.

## 12. *Strain* nas Cardiopatias Congênitas

Alguns estudos já demonstraram o elevado valor prognóstico do *strain* obtido pelo *speckle tracking*, reforçando sua utilidade tanto em patologias congênitas como adquiridas.^9^No entanto, o *strain* miocárdico está sujeito a variações fisiológicas causadas por idade, sexo, frequência cardíaca, pré-carga, pressão arterial e superfície corpórea, além do tipo de *software* utilizado para análise.^296^ Um esforço contínuo vem sendo realizado no sentido de estabelecer valores normais do *strain* que possam ser utilizados como referência universal em pediatria, para que a avaliação da deformação miocárdica seja adotada na rotina clínica.^297-299^

Apresentaremos, nas tabelas a seguir, os valores de *strain* miocárdico já apresentados na literatura, em crianças normais (Tabelas 12.1 [Table t19] a 12.3) e em algumas cardiopatias congênitas, com recomendações de valores de corte (Tabela 12.4).

**Tabela 12.1:** – Valores normais de strain de ventrículo esquerdo, segundo as faixas etárias**298**

Idade em anos	*Strain* global de pico sistólico longitudinal	*Strain* de pico sistólico longitudinal corte apical 4 câmaras	*Strain* global de pico sistólico circunferencial	*Strain* de pico sistólico circunferencial ao nível dos músculos papilares	*Strain* global de pico sistólico radial	*Strain* de pico sistólico radial ao nível dos músculos papilares
0-1	18,7% (20,8; 16,7)	19,4% (22,2; 16,6)	_____	18,2% (22,6; 13,7)	_____	44,4% (36,6; 52,1)
2-9	21,7% (23,0; 20,5)	21,0% (21,8; 20,2)	24,5% (27,2; 21,7)	20,3% (21,4; 19,1)	48,0% (33,3; 62,8)	50,8% (47,4; 54,1)
10-13	20% (20,8; 19,1)	20,5% (21,7; 19,2)	21,9% (26,5; 17,4)	21,5% (23,1; 19,8)	43,7% (33,0; 54,5)	52,1% (48,5; 55,8)
14-21	19,9 (20,6; 19,2)	19,9% (21,2; 18,6)	16,4% (23,3; 9,6)	16,4% (23,3; 9,6)	44,0% (41,6; 46,4)	46,4% (39,7; 53,1)
Geral	20,2% (20,8; 19,6)	20,4% (21,1; 19,8)	22,3% (19,9; 24,6)	20,4% (21,1; 19,8)	45,2% (38,8; 51,7)	49,4% (47,2; 51,6)

*Valores expressos como média e intervalo de confiança de 95%.*

**Tabela 12.2 t19:** – Valores normais de *strain* de ventrículo direito, segundo as faixas etárias300

	31 dias a 24 meses	2 a 5 anos	5 a 11 anos	11 a 18 anos
SLG VD %	25,4 ± 3,9	25,9 ± 4,0	25,8 ± 4,7	25,4 ± 4,1

*SLG: strain longitudinal global; VD: ventrículo direito. Valores expressos como média ± desvio padrão.*

**Tabela 12.3:** – Valores normais de strain atrial direito e esquerdo, segundo as faixas etária**301**

Medida	31 dias a 24 meses	2 a 5 anos	5 a 11 anos	11 a 18 anos
***Strain* AE (R) %**	52,8 ± 10,1	55,7 ± 10,7	58,1 ± 10	57,6 ± 10,5
***Strain* AE (C) %**	14,2 ± 6,6	12,7 ± 6,1	14,0 ± 6,7	15,1 ± 7,0
***Strain* AD (R) %**	47,1 ± 9,6	49,6 ± 10,2	51,6 ± 10,7	52,0 ± 10,6
***Strain AD (C) %***	11,5 ± 6,0	11,9 ± 5,9	11,8 ± 6,3	12,8 ± 5,8

*AD: átrio direito; AE: átrio esquerdo; C: fase de contração atrial; R: fase de reservatório. Valores expressos como média ± desvio padrão.*

**Tabela 12.4:** – Exemplos de estudos que avaliam o uso do strain na hipertensão pulmonar idiopática e em cardiopatias congênitas

	Autores	Parâmetro	Ponto de corte	Achado	S/E
Hipertensão pulmonar idiopática	Muntean et al.^302^	*Strain* segmento médio PL VD	18,5%	Preditor de piora clínica	S: 91,7% E: 30,8% ASC = 0,88 ± 0,06
Anomalia de Ebstein	Kühn et al.^303^	SLG VD (06 segmentos)	20,15%	Diagnóstico de disfunção de VD (FE < 50% à RM)	S: 77% E: 46%
VD sistêmico (PO TGA Senning/Mustard)	Lipczyńska et al.^304^	SLG VD (06 segmentos)	14,2%	Diagnóstico de disfunção de VD (FE < 45% à RM)	S: 83% E: 90% ASC = 0,882
VD sistêmico (TCCGA)	Kowalik et al.^305^	SLG VD (06 segmentos)		Diagnóstico de disfunção de VD (FE < 45% à RM)	S: 77,3% E: 72,7%
PO OACE	Castaldi et al.^306^	*Strain* segmentar de pico do VE	14,8%	Preditor de fibrose à RM	S: 92,5% E: 93,7%
Fisiologia Univentricular (PO Fontan)	Park et al.^307^	*Strain rate* circunferencial	-1,0s^-1^	Preditor de internação prolongada (>14 dias) após anastomose CPT	S: 72% E: 60%

*ASC: área sob a curva; CPT: cavopulmonar total; E: especificidade; FE: fração de ejeção; OACE: origem anômala de artéria coronariana esquerda; PL: parede livre; PO: pós-operatório; RM: ressonância magnética; S: sensibilidade; SLG: strain longitudinal global; TCCGA: transposição corrigida das grandes artérias; TGA: transposição das grandes artérias; VD: ventrículo direito.*

Em breve, um artigo de revisão mais completo sobre o tema será publicado neste periódico.

## 13. *Strain* do Ventrículo Direito

### 13.1. Introdução

O VD tem importante papel na fisiopatologia das doenças cardiopulmonares. Um grande número de evidências tem demonstrado que a disfunção do VD é um importante marcador independente de morbidade e mortalidade em várias situações clínicas como: IC, doenças valvares, HP, embolia pulmonar (EP), cardiopatia isquêmica e na presença de cardiopatia congênita nos adultos.^308-313^

A RMC é considerada o exame não invasivo padrão-ouro para a obtenção dos volumes, FE e avaliação estrutural do VD. Tem, porém, como principais limitações um custo elevado, maior tempo da aquisição das imagens e pouca disponibilidade na maioria dos centros.^314^ A ecocardiografia bidimensional (2D) é o exame inicial mais utilizado na avaliação estrutural e funcional do VD, por ser mais disponível, de menor custo, não invasiva e com menor tempo para aquisição das imagens. Essa avaliação do VD pela 2D, entretanto, é desafiadora, pela estrutura complexa da cavidade, pela posição desfavorável dentro da parede torácica, pela intensa trabeculação miocárdica, que impede a melhor visualização do endocárdio, por possuir paredes mais finas e pela alta dependência das condições de carga dos índices mais utilizados da função sistólica.^315^

Vários parâmetros ecocardiográficos indicadores da função sistólica do VD são utilizados na prática clínica. Recentemente, a ecocardiografia 2D com *strain* pelo *speckle tracking* foi introduzida no cenário clínico como um indicador objetivo de contratilidade miocárdica regional e global, inicialmente na avaliação do VE e, mais recentemente, do VD. Com a aplicação dessa nova metodologia, mais pesquisas e publicações têm chamado atenção das vantagens da sua utilização em relação aos outros parâmetros convencionais ecocardiográficos.^316^

### 13.2. Características Anatômicas e Funcionais do Ventrículo Direito

Na Tabela 13.1, podemos observar as principais características que diferenciam os ventrículos.^317-319^

**Tabela 13.1:** – Características anatômicas e funcionais dos ventrículos

	Ventrículo direito	Ventrículo esquerdo
Estrutura	Via de entrada, região trabeculada, infundíbulo.	Não tem infundíbulo, e a trabeculação é limitada.
Forma	Várias bandas musculares Triangular no plano coronal Crescente no plano transversal	Elíptica
Orientação predominante das fibras miocárdicas	Subepicárdio: circunferencial Subendocárdio: longitudinal	Subepicárdio: oblíqua Mesocárdio: circunferencial Subendocárdio: longitudinal
Disposição das fibras na parede livre	Predomínio transversalmente	Predomínio obliquamente
Disposição das fibras no SIV	Obliquamente com extensão para a via de saída	Obliquamente
Contribuição do espessamento do SIV no eixo transversal e encurtamento no eixo longitudinal na sístole	+++	+++
Padrão de contração	Principalmente longitudinal da base para o ápex	Subepicárdio e subendocárdio: encurtamento longitudinal em direções opostas Mesocárdio: direção circunferencial
Massa (g/m^2^)	26 ± 5	87 ± 12
Espessura da parede (mm)	2 a 5	7 a 11

*SIV: septo interventricular.*

As funções do VE e VD estão intimamente relacionadas, fenômeno chamado de interação ventricular sistólica, pois compartilham fibras musculares dispostas obliquamente no SIV. Elas têm vantagens mecânicas sobre as fibras transversais da parede livre do VD.^36^ A continuidade dessas fibras musculares permite que a parede livre do VD seja tracionada quando ocorre a contração do VE*,* sendo estimado que 20 a 40% do volume sistólico ejetado e da pressão sistólica do VD resultem da contração do VE.^318,319^

### 13.3. Ventrículo Direito e Parâmetros Ecocardiográficos na Avaliação da Função Sistólica

Na avaliação da função sistólica do VD, vários índices são utilizados rotineiramente como a mudança fracional da área (FAC), a excursão sistólica do anel tricúspide (TAPSE), a velocidade de pico sistólico do anel tricúspide e o índice de *performance* miocárdica. Cada um deles tem vantagens e limitações, variável exequibilidade e reprodutibilidade, com discutível eficácia diagnóstica e prognóstica.^35,36^ Acredita-se que, no momento, nenhum deles seja, isoladamente, um bom indicador da função sistólica do VD. Uma vez que o vetor da contração longitudinal é o mais importante, pela orientação das fibras musculares longitudinais predominantes do anel tricúspide ao ápex, dá-se preferência aos índices que exploram a movimentação no eixo longitudinal na avaliação da função longitudinal regional ou global do VD.^92^

A ecocardiografia 2DST é uma modalidade de imagem que avalia a deformação miocárdica, propriedade intrínseca do miocárdio, nas três direções (longitudinal, circunferencial e radial), sendo a longitudinal a mais utilizada por sua boa reprodutibilidade, relevante informação prognóstica, validação em estudo experimental^75^ e em estudos clínicos com RMC em várias doenças CV.^320-323^

Assim*,* o 2DST do VD tem se mostrado um bom marcador da função sistólica, com valor prognóstico em várias doenças CV.^75,324-330^

### 13.4. Aquisição e Limitações

**Figura 13.1:**
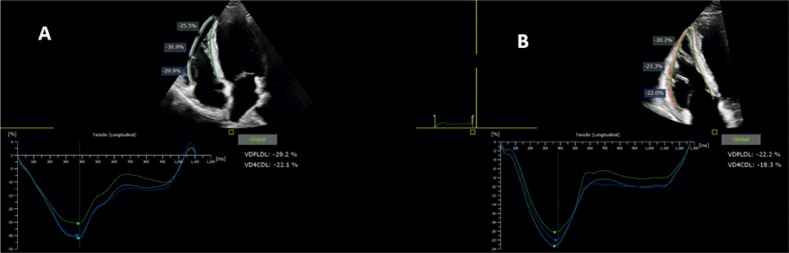
– Protocolo de imagem para medir a deformação do ventrículo direito (VD) por meio da ecocardiografia speckle tracking na projeção apical 4 câmaras. A) Janela apical 4 câmaras (forma de aquisição inadequada); B) Janela apical 4 câmaras focada no VD (forma de aquisição adequada). VD4CDL: strain global longitudinal do ventrículo direito; VDPLDL: strain de parede livre do ventrículo direito.

O SLGVD pelo 2DST deve ser obtido através da janela apical 4 câmaras modificada, focada no VD, com o transdutor deslocado mais lateralmente e direcionado para o ombro direito, o que permite a melhor visibilização da parede livre e reprodutibilidade das medidas (Figura 13.1). É importante otimizar a orientação, a profundidade e o ganho e, com isso, maximizar o tamanho do VD e visualizar o seu ápice durante todo o ciclo cardíaco.^36^ Um outro cuidado na aquisição é não anteriorizar ou posteriorizar o transdutor, evitando, respectivamente, o aparecimento da valva aórtica ou do seio coronário, exibindo apenas o septo interatrial.^331^ Uma vez que a visualização adequada for obtida, recomenda-se ajustar o aparelho para gravar três ciclos cardíacos e adquirir imagens com uma resolução temporal de 50–80 quadros por segundo. Essa taxa de enquadramento pode ser obtida por ajustes indiretos, como por meio da profundidade da imagem e da abertura do feixe de ultrassom e resolução, como também por ajustes diretos permitidos pelo aparelho de ecocardiograma utilizado. Em alguns *softwares*, ainda se faz necessária a definição do início e do fim do tempo de ejeção do VD por meio do Doppler pulsado registrado na via de saída do VD.

A ROI é definida pela borda endocárdica incluindo a parede livre do VD e SIV, com o cuidado de não incluir o pericárdio e ajustar a largura da ROI para não ficar muito estreita, pois isso pode levar a resultados errôneos. Atenção deve ser dada no posicionamento dos pontos de referência basais, pois se estiver abaixo do ideal, ou seja, no lado atrial do anel tricuspídeo, pode resultar em valores reduzidos da deformação longitudinal.^332^

A ROI pode ser traçada manualmente pelo usuário ou gerada automaticamente. Se for gerada automaticamente, o usuário deve ter permissão para verificar e, eventualmente, editá-la de modo manual.^92^ Depois de verificar a qualidade do rastreamento e sua aprovação final pelo operador, os valores de deformação regional serão exibidos. Pelas recomendações atuais, o valor utilizado deve ser o maior valor modular alcançado durante a sístole (*strain* de pico sistólico), sendo o traçado do Doppler da valva pulmonar utilizado para determinar o final da diástole e da sístole.^92^ Sempre que possível, deve-se utilizar um *software* apropriado, já que o algoritmo de detecção automática dos segmentos do VD reduz a necessidade de intervenções por parte do operador, contribuindo, assim, para uma melhor reprodutibilidade dos resultados.

A segmentação da parede livre do VD entre o ápice e a base inclui três segmentos (segmentos basal, médio e apical). O SIV é segmentado de maneira semelhante. O *strain* longitudinal da parede livre do VD (SL-PLVD) é a média dos valores de deformação dos seus três segmentos, enquanto o SLGVD é a média dos valores do *strain* dos segmentos de sua parede livre e do SIV. O SL-PLVD é o mais utilizado na prática e na pesquisa clínica, uma vez que o SLGVD sofre interferência da função ventricular esquerda pelo SIV, obtendo, assim, valores absolutos relativamente mais baixos.^333^ Para fins de padronização, deve-se relatar como parâmetro padrão o SL-PLVD, sendo opcional o cálculo do SLGVD.^92^

Como limitações, além de janelas acústicas inadequadas, estudos experimentais e modelos matemáticos mostraram que a magnitude da deformação miocárdica é influenciada pela frequência cardíaca, além da pré- e pós-carga. Com função sistólica preservada, estudos confirmaram que o *strain* pode aumentar com o aumento da pré-carga e da frequência cardíaca e pode reduzir com o efeito contrário dessas variáveis.^16,334^

### 13.5. Indicações/Valores de Normalidade

A disfunção sistólica do VD é bem estabelecida como um fator de prognóstico ruim em várias doenças CV, e o SLGVD é um marcador prognóstico independente nos pacientes com HP, IC, cardiopatia isquêmica e outras CMPs, assim como tem melhor correlação com a FE do VD pela RMC em comparação aos parâmetros tradicionais.^35,320-23,334-336^

**Figura 13.2:**
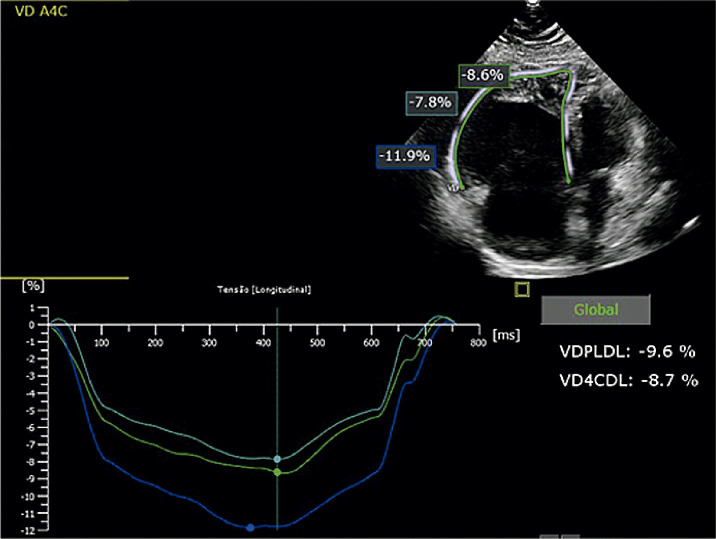
– Exemplo de strain de ventrículo direito em paciente com hipertensão pulmonar primária de longa data. Software Tomtec. VD4CDL: strain global longitudinal do ventrículo direito; VDPLDL: strain de parede livre do ventrículo direito.

Nos pacientes com HP, o SLGVD está diminuído, mostrando uma boa correlação com os parâmetros hemodinâmicos invasivos da *performance* do VD.^337^ Além disso, foi demonstrado que o SLGVD é preditor independente de mortalidade por todas as causas e eventos relacionados à HP. Com o objetivo de avaliar o valor prognóstico do SLGVD nesses pacientes, uma metanálise recente mostrou que sua redução relativa de 19% está associada a um maior risco de eventos relacionados à HP, enquanto a redução relativa de 22% do SLGVD está associada a um maior risco de morte por todas as causas.^324^ A Figura 13.2 mostra um exemplo de *strain* de VD em um paciente com HP primária de longa data.

**Figura 13.3:**
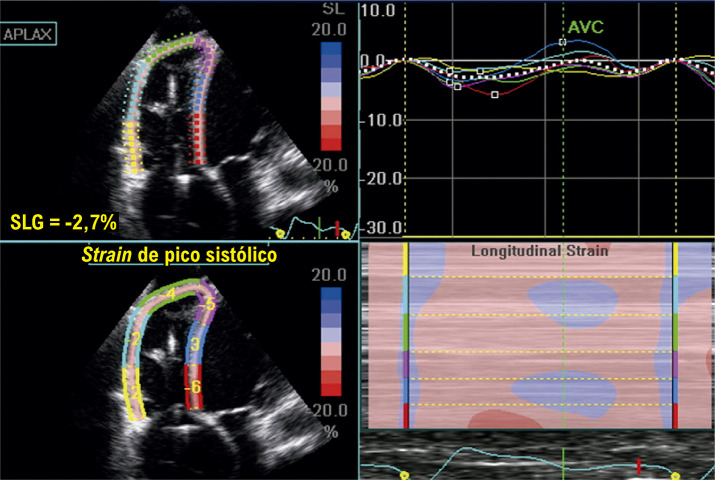
– Exemplo de strain de ventrículo direito em paciente em uso de dispositivo de assistência ventricular. Software Echopac GE. SLG: strain de parede livre do ventrículo direito.

Em pacientes com IC, o SLGVD tem elevada sensibilidade e acurácia no diagnóstico da disfunção sistólica dessa câmara cardíaca.^338^ Uma publicação recente evidenciou que valores absolutos < 14,8% estão associados a eventos adversos como morte, transplante cardíaco e hospitalização, independentemente da FEVE e DD do VE.^19^ Além disso, o SLGVD e o SL-PLVD foram capazes de detectar anormalidades sutis da função sistólica do VD em pacientes com IC e FEVE reduzida e em menor grau nos com IC e FEVE preservada.^329^ Quanto aos pacientes elegíveis para o implante de dispositivo de assistência ventricular esquerda, o SLGVD é uma ferramenta útil na estratificação de risco de falência do VD. Com uma sensibilidade de 68% e uma especificidade de 76%, o valor absoluto de SLGVD < 9,6% foi capaz de identificar os pacientes que evoluíram com falência do VD pós-procedimento, definida como necessidade de dispositivo de assistência ventricular direita ou uso de inotrópicos por mais de 14 dias.^339^ Já em transplantados cardíacos, a combinação das medidas do SLGVE e o SL-PLVD pode ser útil para excluir rejeição celular aguda e reduzir o número de biópsias de rotina.^340^ As Figuras 13.3 e 13.4 mostram exemplos de *strain* de VD em paciente com dispositivo de assistência ventricular e em paciente transplantado cardíaco, respectivamente.

**Figura 13.4:**
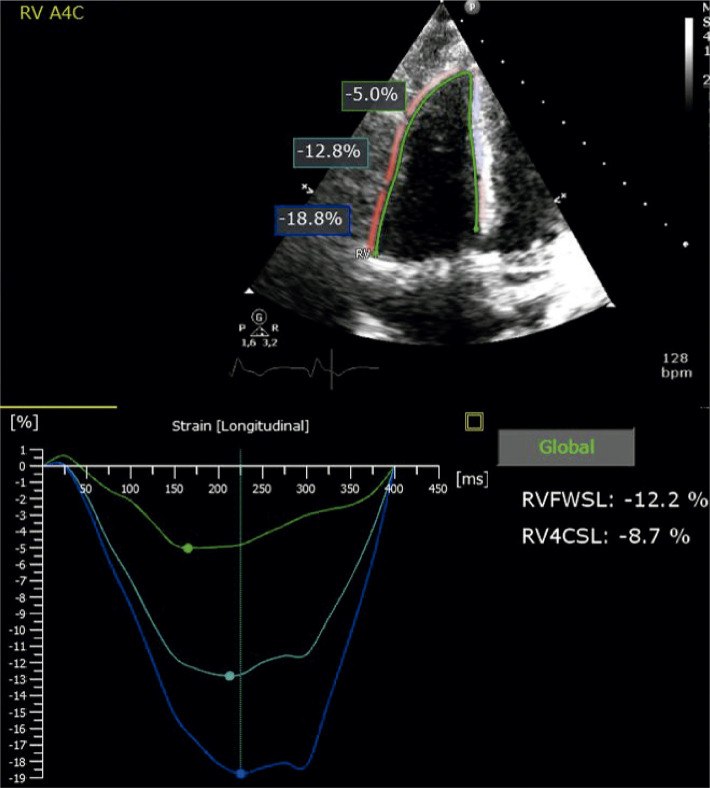
– Exemplo de strain de ventrículo direito em paciente transplantado cardíaco. Software Tomtec. RV4CDL: strain global longitudinal do ventrículo direito; RVFWSL: strain de parede livre do ventrículo direito.

No IAM, o SLGVD é o parâmetro ecocardiográfico que tem melhor correlação com a fração de ejeção do ventrículo direito (FEVD) obtida pela RMC.^341^ Somado a isso, esse parâmetro demonstrou ser preditor independente de morte, reinfarto e hospitalização por IC, confirmando seu papel fundamental na avaliação dessa população.^342^A avaliação de pacientes com displasia arritmogênica do VD é discutida em outra sessão.

Recentemente, o papel da disfunção sistólica do VD tem sido investigado em outras CMPs. Na CMPH, foram descritos valores de SLGVD reduzidos em relação a um grupo-controle saudável,^343^ e também diferenciou pacientes com CMPH e hipertrofia secundária à hipertensão, com alta sensibilidade e especificidade.^344^

A estenose mitral é a doença valvar cardíaca que mais acomete o VD, com alteração frequente dos parâmetros convencionais da sua avaliação. O SLGVD demonstra um padrão de alteração segmentar, com valores significativamente menores no SIV e na parede livre basal do VD e valores normais na parede livre média e apical.^345,346^ Em pacientes com insuficiência tricúspide funcional importante, o SL-PLVD identificou, em maior proporção, os pacientes com disfunção do VD (84,9%) em comparação à FAC (48,5%) e à TAPSE (71,7%). Além disso, o SL*-*PLVD esteve associado de maneira independente com a mortalidade por todas as causas e teve um valor prognóstico incremental quando associado aos parâmetros tradicionais de avaliação do VD.^328^

Atualmente, falta um consenso em relação aos valores normais de *strain* do VD devido à escassez de estudos nessa área. O último documento de recomendações para quantificação das câmaras cardíacas pela ecocardiografia em adultos da Sociedade Americana de Ecocardiografia e da Associação Europeia de Imagem Cardiovascular sugere que valores, tanto do SLGVD quanto do SL-PLVD*,* inferiores a 20% sejam considerados como anormais.^35^ No entanto, é preciso cautela, pois os diferentes tipos de aparelho trazem *softwares* diferentes, com valores de referência particulares e diferenças quanto ao nível de mapeamento (endocárdico, epicárdico ou abrangendo toda a parede miocárdica).

A Tabela 13.2 resume as principais recomendações da utilização do SLG na avaliação do VD.

**Tabela 13.2:** – Indicações da utilização do *strain* longitudinal global na avaliação do ventrículo direito

•Hipertensão pulmonar (incluindo pacientes com embolia pulmonar aguda ou crônica)
•Insuficiência cardíaca com fração de ejeção reduzida ou preservada
•Infarto agudo do miocárdio
•Cardiomiopatias (DAVD, CMPH, CMD)
•Valvopatias (estenose mitral, insuficiência tricúspide funcional)
•Candidatos a implante de dispositivo de assistência ventricular
•Transplante cardíaco

*CMPH: cardiomiopatia hipertrófica; CMD: Cardiomiopatia dilatada; DAVD: displasia arritmogênica de ventrículo direito.*

## 14. *Strain* do Átrio Esquerdo e do Átrio Direito

### 14.1. Técnica de Obtenção e Análise do strain do Átrio Esquerdo

A análise da função do AE usando o *strain* permite que os três componentes da função do AE sejam analisados: AESr, que avalia a função reservatório; AEScd, que avalia a função de conduto; e o AESct, que avalia a função contrátil. Embora menos utilizada, também há a taxa de deformação ou *strain rate*, descrita como pAESRr (pico *strain rate* na fase reservatório), pAESRcd (pico de *strain rate* na fase de conduto) e pAESRct (pico de *strain rate* durante contração atrial).^92,347^

Para a análise do *strain* do AE, usam-se imagens apicais 4 câmaras e 2 câmaras otimizadas para o AE e com frequência de quadros alta, habitualmente entre 40 e 80 fps. Seleciona-se um ciclo cardíaco específico e faz-se manualmente o traçado ponto a ponto a partir da borda endocárdica do anel mitral até o anel mitral oposto, extrapolando-se a entrada das veias pulmonares e do apêndice atrial esquerdo. O *software* cria a ROI, a qual é ajustada para 03 mm de largura e deve cobrir da borda endocárdica até a epicárdica. Caso a qualidade do *tracking* seja reprovada em dois ou mais segmentos, mesmo após ajuste manual, deve-se excluir essa incidência da análise. Finalmente, o *software* calcula o SLG para cada uma das janelas apicais citadas acima.

Na análise do *strain* do AE, há dois métodos diferentes como ponto de referência ou zero: o início da onda P do ECG^2^ ou o pico da onda R do QRS.^348^ O primeiro método permite o reconhecimento mais fácil dos componentes do *strain* do AE, sendo necessária a soma dos valores absolutos de AEScd e AESct, para se obter o AESr. O segundo método oferece diretamente o valor do AESr, que é o dado com maior valor prognóstico, sendo que os demais componentes são obtidos a partir do gráfico. O método que usa a onda R como referência é o mais recomendado, porque esse é o ponto de menor volume do AE, sendo o AESr mais facilmente obtido. A maior parte dos trabalhos usa esse método.^92^

### 14.2. Valores de Normalidade

O *strain* do AE apresenta grande heterogeneidade na literatura quanto aos valores de normalidade. A metanálise realizada por Pathan et al*.* é a melhor evidência no momento: os valores médios do AESr, AEScd e AESct foram, respectivamente: 39,4% (IC95% 38%–40,8%); 23% (IC95% 20,7%–25,2%) e 17,4% (IC95% 16,0%–19,0%).^37^

### 14.3. Aplicabilidade Clínica do *Strain* do Átrio Esquerdo

**Figura 14.1:**
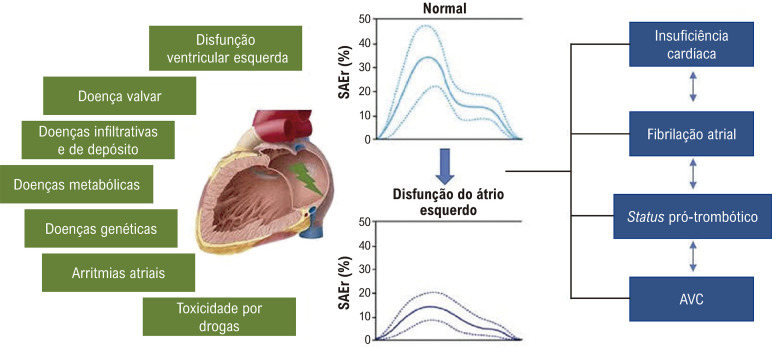
– Mecanismos envolvidos na disfunção atrial esquerda avaliada pelo strain.

A avaliação do *strain* do AE demonstrou valor prognóstico incremental em diversos contextos clínicos, quando comparada à mensuração volumétrica isolada^349^ (Figura 14.1).

#### 14.3.1. Insuficiência Cardíaca e Avaliação de Função Diastólica

O *strain* do AE está deprimido na ICFEr e possui valor prognóstico para previsão de morte por todas as causas ou de nova internação por IC,^350^ boa correlação com capacidade funcional^351^ e pressões de enchimento do VE,^352^ além de ser bom preditor de resposta à terapia de ressincronização miocárdica.^353^ Na ICFEp, o *strain* do AE apresenta importante papel no diagnóstico ^354^ e prognóstico,^73,355^ além de ser capaz de predizer o risco de evolução para FA.^98^

Cerca de 20% dos casos de ICFEp podem ter padrão indeterminado na avaliação da função diastólica do VE,^93^ e o *strain* do AE é capaz de recategorizar esses pacientes,^90^ sendo que os três componentes da função atrial demonstraram boa acurácia em determinar aumento da pressão atrial esquerda.^89^

#### 14.3.2. Fibrilação Atrial

Na FA, as funções de reservatório e conduto do AE estão deprimidas e a contrátil é ausente. O *strain* do AE é capaz de prever FA nova em diversas patologias como ICFEr,^356^ estenose mitral,^357^ doença de Chagas^358^ e após implante de marca-passo,^359^ além de prever o risco de recorrência de FA após cardioversão^360^ ou ablação.^361-363^ É possível que a avaliação da função do AE pelo *strain* seja incorporada no processo decisório da indicação de ablação de FA. O AESr também está associado com ocorrência de AVCi independente de CHA2DS2-VASc escore, idade e uso de anticoagulante.^364^

#### 14.3.3. Valvopatias

O *strain* do AE pode sinalizar maior gravidade e evolução desfavorável na valvopatia mitral e aórtica.^365,366^ Na IM primária grave, o AESr demonstrou ser preditor de hospitalização por IC ou morte por todas as causas, independentemente das indicações de intervenção cirúrgica.^367,368^

#### 14.3.4. Doença Arterial Coronariana

A DAC associa-se à disfunção atrial por dois mecanismos principais: DD do VE e isquemia direta do AE.^369^ O *strain* do AE pode ter importante valor prognóstico na síndrome coronariana aguda, correlacionando-se com maior gravidade^370^ e desfechos desfavoráveis.^371^

## 14.4. *Strain* Atrial Direito

O *strain* do AD carece de dados, mas um estudo recente avaliou 101 voluntários saudáveis e descreveu os seguintes valores utilizando o complexo QRS como referência: reservatório (37,6% ± 6,9), conduto (26,0% ± 7,1) e contração (11,6% ± 4,4).^372^ A avaliação da função do AD é alvo de interesse em cardiopatias congênitas,^301,373^ valvopatia tricúspide e HP.^374^

## 15. Avaliação da Torção do Ventrículo Esquerdo

### 15.1. Introdução

A função do VE é determinada pelas interações complexas entre a anatomia do tecido, a contratilidade miocárdica e hemodinâmica. No miocárdio do VE, as fibras musculares possuem direções diferentes. Na região subendocárdica, as fibras são quase paralelas à parede e produzem um movimento de rotação do tipo direito (hélice de mão direita), o que gradualmente muda no subepicárdico para fibras anguladas a 60–70 graus, promovendo uma rotação do tipo esquerda (hélice de mão esquerda).^375,376^

**Figura 15.1:**
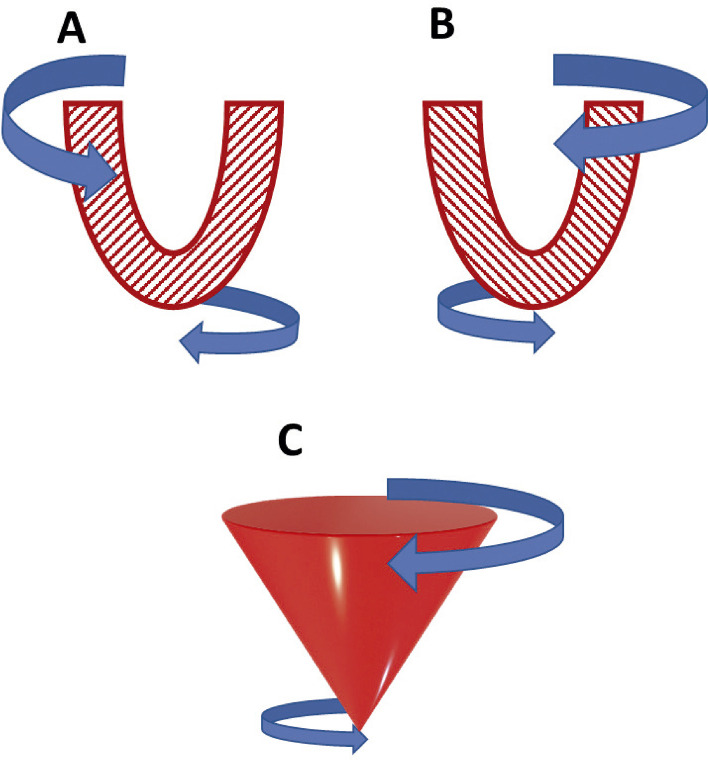
– Direção da rotação das fibras subendocárdicas (A); direção da rotação das fibras subepicárdicas (B); e rotação resultante geral do ventrículo esquerdo com a contração simultânea das fibras (C). Adaptado de Stöhr et al.*384*

A contração das fibras subepicárdicas faz com que o ápice do VE gire no sentido anti-horário e a sua base no sentido horário. Por outro lado, a contração das fibras subendocárdicas faz o ápice e a base do VE girarem exatamente nas direções opostas. Dado ao maior raio de rotação da camada epicárdica, a direção das fibras subepicárdicas prevalece na direção geral de rotação quando ambas as camadas se contraem simultaneamente. Isso resulta em rotação global do VE no sentido anti-horário próxima ao ápice e na rotação no sentido horário próxima à base do VE durante a ejeção ventricular,^377^ como ilustrado na Figura 15.1.

Esse movimento de torção do VE contribui para manter uma distribuição uniforme do encurtamento e do estresse das fibras ao longo de todas as paredes, produzindo, assim, uma FE relativamente elevada (~60%), a despeito de encurtamento limitado (~20%).^378^ A torção e o cisalhamento das fibras subendocárdicas durante a ejeção ventricular resultam no armazenamento de energia potencial, que é subsequentemente usada para o desenrolar diastólico das fibras e, assim, destorcer as hélices, produzindo juntos a sucção diastólica.^379,380^ As condições de pré- e pós-carga e contratilidade alteram a extensão da torção ventricular.^381^ O aumento da pré-carga ou da contratilidade aumentam a torção do VE, enquanto o aumento na pós-carga causa efeito inverso.

Várias modalidades e técnicas de imagem podem ser usadas para quantificar a mecânica da torção ventricular: ecocardiografia (Doppler tecidual, ST2D e ST3D, imagem de velocidade vetorial [VVI]), RMC (*tissue tagging*) e sonomicrometria. Atualmente, não existe um padrão-ouro para a avaliação da mecânica de torção do VE, tendo as modalidades de imagem listadas acima boa concordância.^382^ Devido à sua segurança, disponibilidade e melhor custo/efetividade, a ecocardiografia tem sido a modalidade de imagem mais empregada.

### 15.2. Definições e Nomenclaturas

Torção, *twist, twist rate, untwist, untwist rate* são as terminologias comumente utilizadas para descrever os achados da rotação sistólica e a rotação diastólica reversa da base e do ápice do VE, como vistos pelo ápex. As definições desses termos podem ser encontradas nas Tabelas 15.1 e 15.2.

**Tabela 15.1:** – Definições e parâmetros utilizados para avaliação do mecanismo de *twist* do ventrículo esquerdo na sístole

Parâmetro	Definição
Rotação apical (º)	Pico da rotação sistólica no sentido anti-horário da região apical do VE (medido em graus)
Taxa de rotação apical (º/s)	Pico de velocidade de rotação apical no sentido anti-horário (medido em graus/segundo)
Rotação basal (º)	Pico da rotação sistólica no sentido horário da região basal do VE (medido em graus)
Taxa de rotação basal (º/s)	Pico de velocidade de rotação basal no sentido horário (medido em graus/segundo)
*Twist* VE (º)	Diferença do pico das rotações sistólicas do ápice e da base do VE (medido em graus)
Torção do VE (º/cm)	*Twist* normalizado: razão do ângulo do twist pela distância entre a base e o ápice na sístole (medido em graus/centímetro)
*Twist* rate (º/s)	Velocidade de pico do *twist* do VE (medido em graus/segundo)

*(o): graus; (o/s): graus por segundo; (º/cm): graus por centímetro; VE: ventrículo esquerdo.*

**Tabela 15.2:** – Definições e parâmetros utilizados para avaliação do mecanismo de twist do ventrículo esquerdo na diástole

Parâmetro	Definição
Rotação reversa apical (º)	Pico da rotação diastólica no sentido horário da região apical do VE (medido em graus)
Taxa de rotação reversa apical (º/s)	Pico de velocidade da rotação reversa apical no sentido horário (medido em graus/segundo)
Rotação reversa basal (º)	Pico da rotação diastólica no sentido anti-horário da região basal do VE (medido em graus)
Taxa de rotação reversa basal (º/s)	Pico de velocidade da rotação basal (medido em graus/segundo)
*Untwist* (º)	Diferença do pico das rotações diastólicas reversas do ápice e da base do VE (medido em graus)
*Untwist* rate (º/s)	Velocidade de pico do untwist do VE (medido em graus/segundo)

*(o): graus; (o/s): graus por segundo; VE: ventrículo esquerdo.*

### 15.3. Passo a Passo da Avaliação da Torção Ventricular pelo Ecocardiograma com *Speckle Tracking*

**Figura 15.2:**
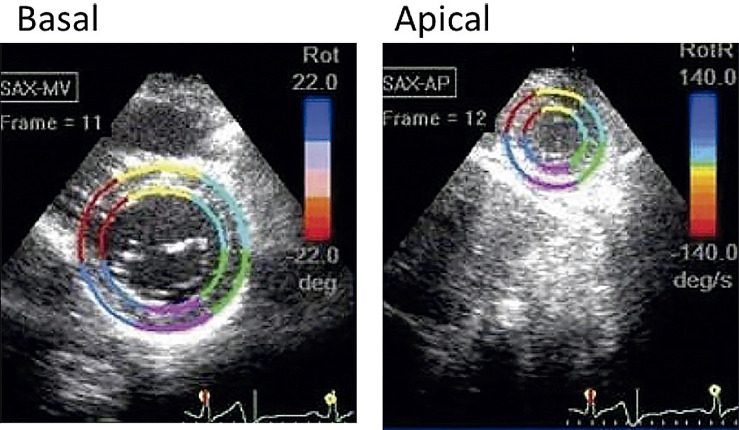
– Planos de obtenção das imagens para medida da rotação basal e apical. Imagens cedidas pelo Dr. Marcio Lima.

Para avaliação do mecanismo de rotação, são obtidas imagens paraesternais do eixo curto do VE ao nível basal (valva mitral) e apical (abaixo dos músculos papilares) (Figura 15.2). É importante obter a imagem apical do VE onde não apareça o VD ou apenas uma parte deste, em geral um a dois espaços intercostais abaixo da posição habitual. A maioria dos erros de avaliação ocorre devido à seleção inapropriada dos planos basal e apical e do ajuste da ROI.

**Figura 15.3:**
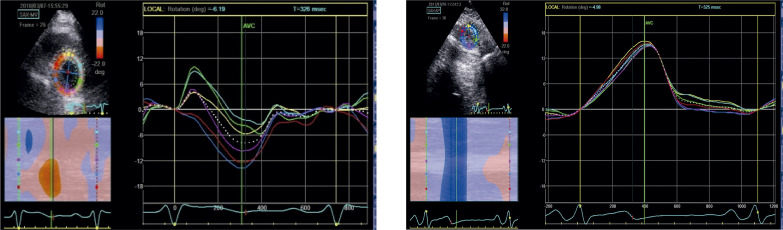
– Registro das rotações horária (abaixo da linha de base) e anti-horária (acima da linha de base). Imagens cedidas pelo Dr. Marcio Lima.

Por convenção, quando a rotação é horária, o traçado é registrado abaixo da linha de base e, quando a rotação é anti-horária, o traçado é inscrito acima da linha de base (Figura 15.3).

O valor normal do *twist* global é de 9,7° ± 4,1°. Para a torção, há poucos valores de referência na literatura, sendo estimada em 1,35°/cm ± 0,54°/cm.^383^

### 15.4. Aplicações Clínicas

Os parâmetros de torção do VE têm sido usados principalmente para avaliar as alterações na mecânica ventricular que ocorrem em patologias com FEVE reduzida (CMP isquêmica e dilatada) ou preservada (ICFEp, hipertensão, CMPH, EA, IAo e IM), bem como na avaliação de disfunção miocárdica subclínica causada por quimioterápicos.

A medida do *twist* e da torção, embora sejam bons parâmetros para ajudar na análise da função sistólica global, tem limitações quanto à reprodutibilidade, em especial devido à falta de parâmetros anatômicos para o corte apical. Os achados de alterações no *twist* e torção ventricular não são específicos, mas podem contribuir para o entendimento da fisiopatologia de diferentes CMPs, auxiliando na diferenciação entre elas (Tabela 15.3).

**Tabela 15.3:** – Twist do ventrículo esquerdo em diferentes doenças cardiovasculares

Doença cardiovascular	*Twist* do VE	Achados
Cardiomiopatia isquêmica	Diminuído	*Twist* diminui dependendo da localização e extensão da isquemia.
Cardiomiopatia dilatada	Diminuído	Diminuição do *twist* proporcional à queda da fração de ejeção
Cardiomiopatia hipertrófica	Aumentado	Aumento do *twist* em especial se houver obstrução da via de saída do VE
Estenose aórtica	Aumentado	Aumento do *twist* em caso de aumento da pós-carga do VE.

*VE: ventrículo esquerdo.*

## 16. *Strain* na Análise da Dissincronia Ventricular

### 16.1. Introdução

A terapia de ressincronização cardíaca (TRC) é uma opção terapêutica com indicações já estabelecidas em diretrizes nacionais e internacionais e redução expressiva comprovada em morbidade e mortalidade. Ela é recomendada como classe I para pacientes com CMP dilatada, sintomáticos, em tratamento clínico otimizado, ECG com padrão de BRE, duração do QRS ≥ 150 ms e FEVE abaixo de 35% (nível de evidência: A).^385^

Essas diretrizes empregam a presença de BRE com duração ≥ 150 ms ao ECG como marcador de dissincronia devido à ausência de evidências da utilidade da avaliação ecocardiográfica da sincronia até o momento. Entretanto, o ECG também apresenta limitações como marcador de dissincronia. Assim, atualmente, a avaliação ecocardiográfica da dissincronia para a seleção da TRC deve ser realizada de maneira individualizada e criteriosa por um examinador com treinamento adequado e interpretada juntamente aos dados clínicos do paciente. Também é importante lembrar que o papel da ecocardiografia engloba não apenas a avaliação da sincronia cardíaca na seleção dos pacientes, mas também o auxílio na escolha do melhor local para o implante do eletrodo VE, além de avaliação da resposta e remodelamento reverso e, mais recentemente, a identificação do risco de arritmias ventriculares.^385^

### 16.2. Avaliação da Dissincronia na Seleção dos Pacientes para a Terapia de Ressincronização Cardíaca

A avaliação da dissincronia pelo *strain*, isoladamente, não indica a TRC, nem a análise de sua eficiência. Entretanto, reconhece-se que, mesmo com a indicação precisa, a chance de sucesso, isto é, melhora clínica, funcional e/ou de variáveis obtidas por métodos de imagem, fica em torno de 60–70% dos casos. A taxa de resposta à TRC pode ser estimada e até melhorada com o emprego da ecocardiografia. Nesse panorama, a medida de valores da deformação miocárdica se destaca.

**Figura 16.1:**
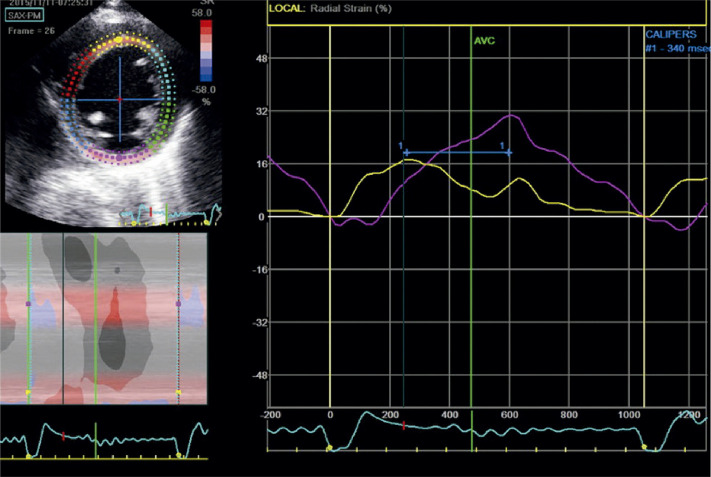
– Imagem de strain radial com curvas dos segmentos médios das paredes anterosseptal e inferolateral. O intervalo acima de 130 ms correlaciona-se com uma maior taxa de resposta à terapia de ressincronização cardíaca; nesse caso, 340 ms.

A análise inicial da dissincronia pelo *strain* radial descreveu a diferença de tempo entre a deformação máxima radial dos segmentos médios anterosseptal e inferolateral. Uma medida com valor superior a 130 ms caracteriza pacientes com maior taxa de resposta,^386^ como mostrado na Figura 16.1.

**Figura 16.2:**
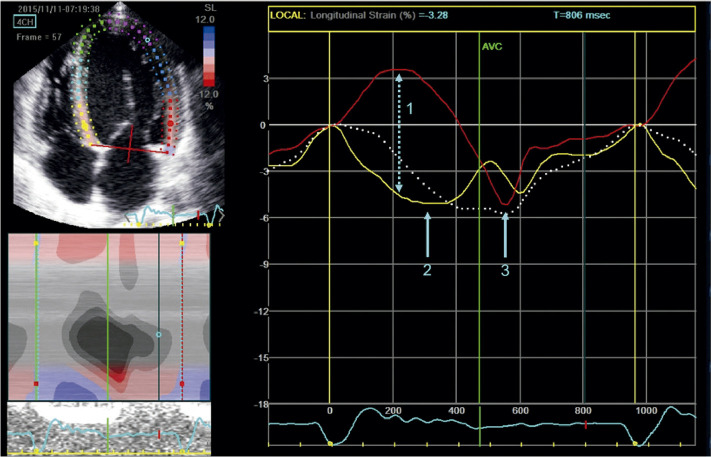
– Imagem do strain longitudinal com curvas dos segmentos basais inferosseptal (amarela) e anterolateral (vermelha) com padrão de bloqueio de ramo esquerdo típico: 1) oposição do pico das curvas septal (negativa) e lateral (positiva), inicialmente; 2) pico de deformação negativa do septo em até 70% do tempo de ejeção, com o encurtamento sendo interrompido durante a sístole, isto é, antes do fechamento da valva aórtica (AVC), resultando em estiramento sistólico; 3) pico de deformação negativa de parede lateral após o fechamento da valva aórtica.

Além da dissincronia radial, a identificação de um padrão de estiramento rebote da parede septal (SRS, *septal rebound stretch*) pela técnica de *speckle tracking* demonstrou-se um preditor independente de prognóstico em longo prazo, além de remodelamento reverso ventricular esquerdo com valor incremental à presença de BRE e de *apical rocking* detectada pela análise visual. Esse padrão reflete à incoordenação da contração cardíaca com resultante redução da *performance* miocárdica. Assim, estudos recentes indicam que a presença de SRS pode melhorar a seleção dos pacientes na TRC, especialmente no subgrupo de pacientes sem BRE definido.^387^ A denominação desse padrão clássico é feita por meio de três elementos obtidos pelo padrão de deformação longitudinal dos segmentos (frequentemente basais) inferosseptal e anterolateral: 1) oposição de pico das curvas septal (negativa), lateral (positiva) inicialmente; 2) pico de deformação negativa do septo em até 70% do tempo de ejeção; 3) pico de deformação negativa de parede lateral após fechamento da valva aórtica,^388^ conforme demonstrado na Figura 16.2.

**Figura 16.3:**
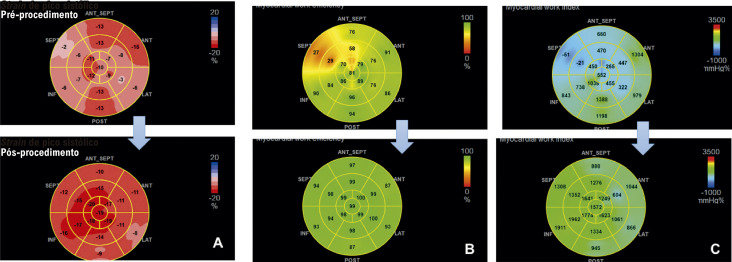
– Mapas polares comparativos pré- e pós-procedimento do strain longitudinal (A), do myocardial work (B), myocardial efficiency (C) em paciente submetido a terapia de ressincronização com sucesso.

Recentemente, a análise da eficiência do TM global ventricular esquerdo (GLVMWE, *global left ventricular myocardial work efficiency*) tem se mostrado promissora no contexto da TRC. O GLVMWE pode ser quantificado de maneira não invasiva a partir das curvas de *strain* miocárdico e medidas da pressão arterial. Valores reduzidos de GLVMWE estiveram associados, de maneira independente, a melhor prognóstico em longo prazo.^389^ Na Figura 16.3, exemplificam-se as modificações ocorridas no *strain, myocardial work* e *myocardial efficiency* em paciente submetido a TRC com sucesso.

### 16.3. Avaliação de Viabilidade Miocárdica

Outra aplicação da deformação miocárdica no contexto da TRC refere-se à correlação da presença de fibrose miocárdica com valores reduzidos de *strain*. Valores de *strain* global radial reduzidos correlacionam-se a maior grau de fibrose (detectados por RMC) e, assim, identificam pacientes com chance reduzida de recuperação da função ventricular. A redução da deformação longitudinal em pacientes com cardiopatia isquêmica também pode ser empregada para essa finalidade.

### 16.4. Orientação do Local de Implante dos Eletrodos

Tão importante como a caracterização da fibrose total do VE, a presença de valores comprometidos em segmentos no local de implante do eletrodo do VE tem se relacionado à menor taxa de pacientes respondedores à TRC.

Estudos demonstram que o posicionamento do eletrodo do VE no segmento que apresenta maior atraso da contração mecânica resulta em maiores taxas de sucesso à TRC. A identificação do segmento a ser estimulado pode ser realizada pela técnica de *speckle tracking*. No estudo TARGET, o posicionamento do eletrodo do VE guiado pela técnica de ST2D resultou em melhor resposta clínica e menores taxas de morte e hospitalizações por IC.^390,391^

### 16.5. Avaliação Prognóstica após a Terapia de Ressincronização Cardíaca

A análise da dissincronia pelo *strain* longitudinal após a TRC foi um preditor forte de arritmias ventriculares. A persistência ou o aumento da dispersão mecânica 6 meses após a TRC, avaliada pelo *speckle tracking,* está associada a pior prognóstico. Além disso, a resposta à TRC evidenciada pelo remodelamento reverso foi dependente da melhora tanto da função longitudinal quanto da circunferencial após a TRC.^392^

### 16.6. Ajuste nos Parâmetros de Ressincronização

Cerca de 30% dos pacientes submetidos a TRC são considerados não respondedores devido à ausência de melhora clínica e/ou funcional, além da ausência de remodelamento reverso evidenciada pela redução das dimensões ventriculares e o aumento da FE.^393^ Nesse grupo de pacientes, ajustes dos intervalos atrioventricular, interventricular e intraventricular esquerdo podem melhorar a resposta individual à TRC. Alguns estudos têm demonstrado que a *speckle tracking* pode ser empregada como guia para ajuste dos parâmetros da TRC, com melhora significativa da classe funcional e da FE em pacientes não respondedores.^394^

## 17. *Myocardial Work* (Trabalho Miocárdico)

### 17.1. Introdução

Uma nova ferramenta ecocardiográfica chamada *myocardial work* (MW) surgiu recentemente visando incrementar informações acerca da função ventricular, adicionando o efeito da pós-carga do VE à medida do *strain* longitudinal.

Com o trabalho experimental de Suga et al. em 1979,^395^ demonstrando que a área sob a curva pressão-volume adquirida de forma invasiva com um cateter de condutância intraventricular refletia o trabalho miocárdico (TM) regional e o consumo de oxigênio por batimento, crescia o interesse dos métodos de imagem em tornar essa análise factível de forma não invasiva.^396,397^

**Figura 17.1:**
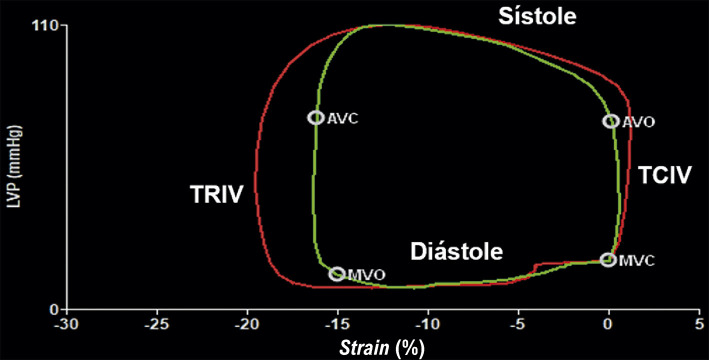
– Alça pressão vs. deformação do ventrículo esquerdo. Em vermelho: alça pressão vs. deformação global do ventrículo esquerdo. Em verde: alça pressão-deformação do segmento basal da parede ínfero-lateral. LVP: pressão do ventrículo esquerdo estimada pela pressão arterial sistólica; AVO: abertura da válvula aórtica; AVC: fechamento da válvula aórtica; MVO: abertura da válvula mitral; MVC: fechamento da válvula mitral.

Russell et al.,^398^ em 2012, validaram a alça pressão-deformação (PD) do VE obtida de forma totalmente não invasiva, integrando a pressão arterial sistólica (PAS) no momento do exame com o *strain* longitudinal utilizando o *speckle tracking*, o que gera, quando interpretadas por um *software* próprio, alças PD global e por segmento (Figura 17.1). A área sob a curva representa o TM e obteve excelente correlação com as medidas diretas intraventriculares. Além disso o TM foi capaz de refletir o metabolismo miocárdico regional de oxigênio, quando comparado à medida pela tomografia por emissão de pósitrons com (18^F^) fluorodesoxiglicose.^399-401^

Aumentos modestos na pressão arterial podem gerar redução de até 9% no SLG, podendo levar a uma errônea interpretação de redução de contratilidade, quando, na verdade, o TM permanece preservado, refletindo apenas uma elevação da pós-carga. Nesse sentido, o TM é considerado um avanço na compreensão da mecânica ventricular.^398,402^ As principais diferenças entre o TM e o *strain* do VE são demonstrados na Tabela 17.1.

**Tabela 17.1:** – Principais diferenças entre o trabalho miocárdico e o *strain* do ventrículo esquerdo

	*Strain*	Trabalho miocárdico
Utiliza AFI^®^	Sim	Sim
Medida realizada	AVO-AVC	TCIV+ Tej + TRIV
Integra com PA	Não	Sim
Valor	%	mmHg%
Incorpora pós carga	Não	Sim
Medida de eficiência	Não	Sim
Estima consumo miocárdico de oxigênio	Não	Sim

*AVO: abertura da válvula aórtica; AVC: fechamento da válvula aórtica; TCIV: tempo de contração isovolumétrico; Tej: tempo de ejeção; TRIV: tempo de relaxamento isovolumétrico.*

### 17.2. Aquisição do Trabalho Miocárdico

Para a obtenção de resultados reprodutíveis e com boa acurácia, é importante o uso de técnica adequada para o cálculo do TM, não só para a aquisição das imagens, mas também para o pós-processamento e a análise dos parâmetros. Essa tecnologia atualmente está apenas disponível em estações de trabalho (*workstations*) ou embarcadas em aparelhos com *software* desenvolvido por apenas um fabricante (GE Healthcare, Horten, Norway). As análises podem ser realizadas diretamente no aparelho ou pós-processadas nas *workstations* a partir de imagens previamente adquiridas.

**Figura 17.2:**
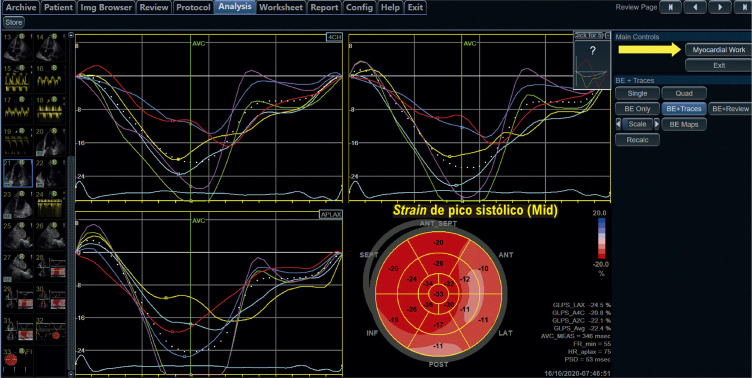
– Após a realização da análise de strain bidimensional longitudinal do ventrículo esquerdo pelo método AFI (automated functional imaging), tendo processado as três janelas apicais, o software oferece a opção de cálculo do trabalho miocárdico (myocardial work) – seta.

**Figura 17.3:**
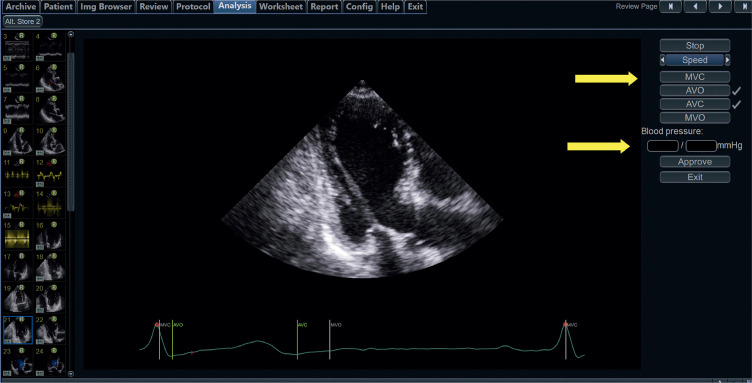
– Tela inicial de cálculo do trabalho miocárdico. Podemos inserir manualmente, nessa etapa, o valor da pressão arterial não invasiva aferida (seta) e reavaliar ou realizar a marcação de eventos cardíacos, como a abertura e fechamento das valvas mitral e aórtica (setas).

O protocolo de obtenção de imagens para o cálculo do TM segue os mesmos pré-requisitos técnicos necessários para a análise do SLG, abordados em capítulo específico. Após a realização das análises de *strain* bidimensional, utilizando imagens adquiridas nas três projeções apicais através da técnica do AFI (*automated functional imaging*), o *software* permite que seja selecionada a análise do TM (Figura 17.2). Como passo inicial, devemos inserir manualmente os valores de pressão arterial não invasiva (PNI) medidos no momento do exame, e isso pode ser realizado a qualquer momento do exame na tela de identificação do paciente ou posteriormente, na própria tela de cálculo de TM (Figura 17.3). Essas medidas de PNI serão automaticamente integralizadas no cálculo de curva de pressão vs. deformação.

**Figura 17.4:**
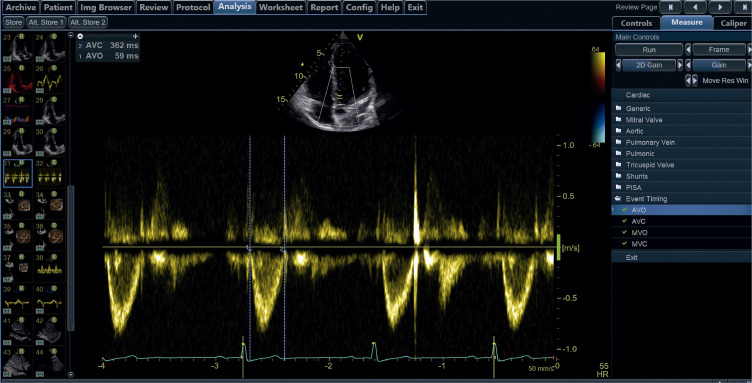
– Marcação de eventos cardíacos com o uso do Doppler espectral. Em A, observamos a marcação de abertura (MVO) e o fechamento da valva mitral (MVC) através do influxo mitral. Em B, observamos a marcação de abertura (AVO) e o fechamento da valva aórtica (AVC) através do fluxo da via de saída do ventrículo esquerdo.

**Figura 17.5:**
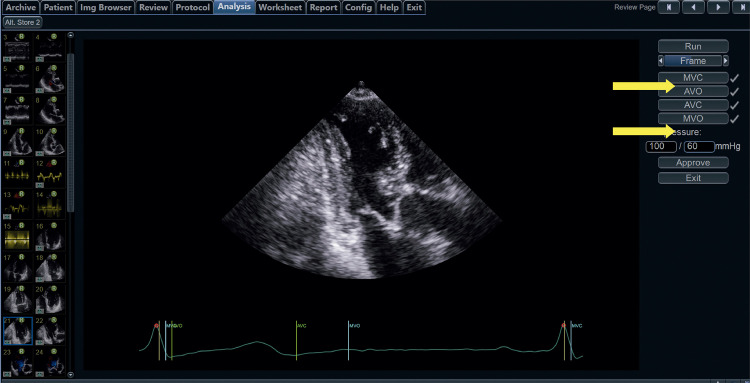
– A marcação de eventos cardíacos também pode ser realizada na própria tela de cálculo do trabalho miocárdico (myocardial work), modificando quadro a quadro (“frame”) a imagem do apical 3 câmaras, selecionando o momento exato de cada evento, clicando nos campos à direita (setas).

**Figura 17.6:**
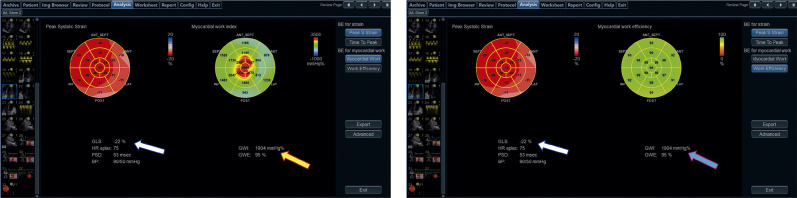
– Após a aprovação dos dados obtidos, a marcação adequada de eventos e o preenchimento de pressão não invasiva, o software dispõe os valores de strain longitudinal global e por segmento à esquerda (setas brancas) e os mapas polares com valores de índice de trabalho miocárdico (A – seta amarela) e de eficiência de trabalho miocárdico (B – seta azul).

**Figura 17.7:**
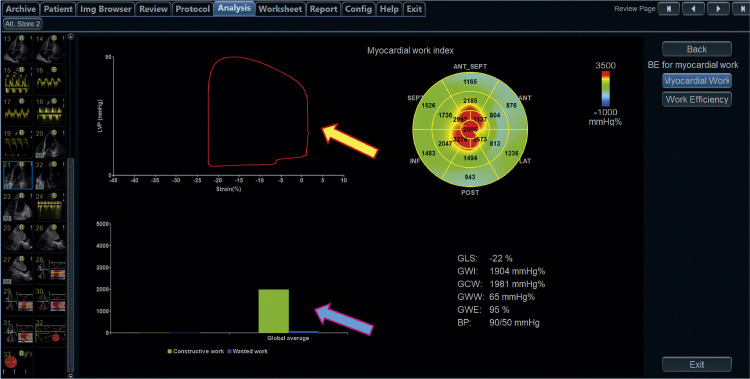
– Quando selecionamos a tecla “advanced”, são geradas as análises por curvas e gráficos, onde é possível observar as alças de pressão do ventrículo esquerdo (left ventricular pressure, LVP) vs. strain ao longo do ciclo cardíaco (seta amarela), além de um gráfico de barras que demonstra a participação do trabalho miocárdico construtivo e do trabalho miocárdico desperdiçado (seta azul).

Para que seja possível obter a indexação temporal dos valores obtidos, é necessário que sejam feitas as marcações de eventos do ciclo cardíaco, identificando a abertura e fechamento das valvas mitral e aórtica, o que pode ser realizado através do Doppler espectral dos fluxos mitral e aórtico, ou ao bidimensional, na análise da projeção apical 3 câmaras, em que podemos evidenciar a abertura e fechamento de ambas as valvas (Figura 17.4). Essas marcações também podem ser realizadas na própria tela de cálculo do TM, modificando quadro a quadro (“*frame*”) a imagem do apical 3 câmaras, selecionando qual o momento exato de cada evento (Figura 17.5). Após aprovar as imagens e marcações realizadas (“*aprove*”), o *software* realiza os cálculos e dispõe lado a lado o mapa polar (*bull’s eye*) com valores de SLG e *strain* de pico por segmento e, à direita o mapa polar com o índice de TM por segmento, dispondo na parte inferior os valores de índice de TM global (GWI) e GMWE. Ao selecionarmos, à direita, a tecla “*work efficiency*” o *software* dispõe no mapa polar os valores de GMWE por segmento (Figura 17.6). Quando selecionamos a tecla “*advanced*”, são geradas as análises por curvas e gráficos, nas quais é possível observar as alças de pressão do VE (*left ventricular pressure*, LVP) vs. *strain* ao longo do ciclo cardíaco, além de um gráfico de barras que demonstra a participação do TM construtivo e do TM desperdiçado (Figura 17.7).

Os seguintes parâmetros são fornecidos pelo *software*:

1. Índice de TM global (ITMG/GWI): corresponde ao trabalho total dentro da área sob a curva PD, sendo calculado a partir do fechamento da válvula mitral até a abertura da valva mitral. Um *bull’s eye* com valores de TM segmentar e global é fornecido (Figura 17.6).

2. TM construtivo (TMC/GCW): é o trabalho que contribui para a ejeção do VE durante a sístole, sendo obtido considerando-se o encurtamento dos miócitos durante a sístole, adicionando o alongamento dos miócitos durante o relaxamento isovolumétrico (Figura 17.7).

3. TM desperdiçado (TMD/GWW): é o trabalho que não contribui para a ejeção do VE, sendo obtido considerando-se o alongamento dos miócitos (em vez de encurtamento) durante a sístole, somado ao encurtamento durante a fase de relaxamento isovolumétrico (encurtamento pós-sistólico) (Figura 17.7).

4. Eficiência do TM (ETM/GMWE): é obtido por meio da fórmula:  TM construtivo/(TM construtivo +TM desperdiçado)  . Seu valor é dado em porcentagem de 0 a 100% de eficiência (Figura 17.6).

### 17.3. Valores de Normalidade

Devido à validação recente do TM e de suas variáveis para o uso clínico, não há ensaios multicêntricos com número adequado de pacientes para gerar valores de normalidade definitivos.

Manganaro et al. recentemente analisaram os dados do estudo NORRE visando estabelecer limites de referência normais para o TM. Esse foi um estudo multicêntrico e prospectivo europeu formado por 226 pacientes oriundos de 22 laboratórios de ecocardiografia, que forneceu valores de referência para a maioria dos dados ecocardiográficos 2D e 3D.^403^ A média ou mediana com o desvio padrão e o intervalo de confiança das variáveis do TM foram, respectivamente, 1.896 + 308 mmHg% (1.292–2.505) para o GWI, 2.232 ± 331 mmHg% (1.582–2.881) para o GCW, 79 mmHg% (53–122) para o GWW e 96% (94–97) para a GMWE.^403^

O GWI e GCW foram maiores em mulheres acima de 40 anos, e existe forte correlação no aumento do GWI e GCW com o aumento da pressão arterial sistólica.^404^

### 17.4. Potencial Uso Clínico

A grande limitação para a democratização do uso do TM é o fato de apenas uma empresa ser detentora do *software* (GE Healthcare). Além disso, o cálculo do TM usa basicamente a pressão sistólica aferida manualmente como medida de pós-carga em sua validação. É importante também levar em consideração situações clínicas em que há um aditivo da pós-carga além da PAS, como na EA, CMPH obstrutiva e em algumas cardiopatias congênitas. Apesar de se tratar de uma nova ferramenta bastante promissora, à luz do conhecimento científico atual, ainda é restrita ao campo da pesquisa.

**Figura 17.8:**
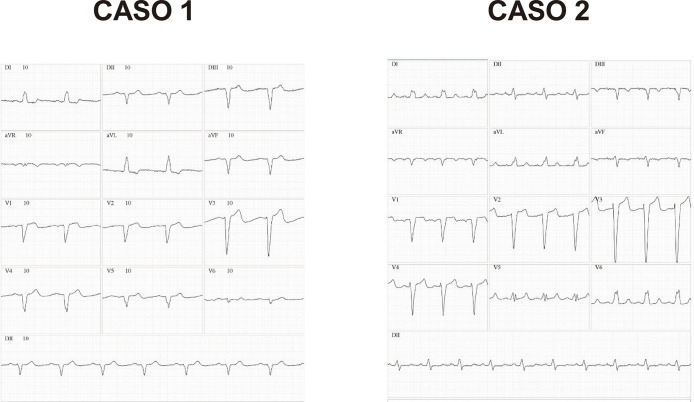
– Casos 1 e 2 com morfologias bem semelhantes de bloqueio de ramo esquerdo.

**Figura 17.9:**
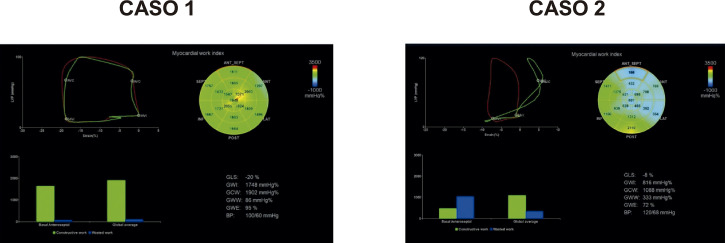
– Caso 1 não apresenta alteração na mecânica miocárdica visualizado pela linha verde em que o trabalho miocárdico na alça pressão-strain é semelhante ao valor global em vermelho. Já no Caso 2, o “verdadeiro” bloqueio de ramo esquerdo, note que na mesma porção septal a curva em verde adquire um formato típico de 8.

Existem algumas publicações que já vêm ganhando notoriedade da aplicação do uso do TM na prática clínica, uma delas é na seleção de pacientes para ressincronização miocárdica. Algumas vezes, a análise do bloqueio do ramo esquerdo pode gerar dúvidas na interpretação (Figura 17.8). Por outro lado, através da análise visual e quantitativa do TM, tornou-se mais fácil o reconhecimento dos casos que têm um ECG com alargamento do QRS e que talvez não tenha um dissincronismo mecânico associado (Figura 17.9). Além da análise visual, tem-se valorizado o valor do trabalho construtivo para identificar os respondedores à terapia de ressincronização, sendo um equivalente de reserva contrátil.^405^ Outro dado interessante para identificar os pacientes que se beneficiam com a ressincronização é a análise do trabalho perdido do septo.^406^ Portanto, a análise do TM nos pacientes com BRE pode ajudar a melhorar a estratificação pela análise visual, assim como a quantificação do trabalho construtivo e o trabalho perdido.

Outro campo interessante na avaliação do TM é na doença coronariana obstrutiva. Ele tem demonstrado sua capacidade de detecção em repouso de doença coronária obstrutiva sendo superior ao SLGVE, mesmo naqueles com FE preservada e sem alteração da contratilidade segmentar.^407^ A sua aplicação também tem demonstrado identificar aqueles pacientes com infarto agudo que irão apresentar mais complicações hospitalares e predizer aqueles com recuperação da função miocárdica^408^ e determinar complicações a longo prazo em pacientes com IAM com supradesnível de ST.^409^

Além das duas situações clínicas descritas, é promissora sua aplicação na CMP dilatada, CMPH e amiloidose. Provavelmente, quando houver mais praticidade do *software* e mais evidência na literatura, a aplicação do TM será incorporada na prática clínica.

## 18. *Strain* no 3D: O Que Pode Acrescentar ao Exame

### 18.1. Introdução

A avaliação tridimensional (3D) da deformação miocárdica das câmaras cardíacas através da técnica de rastreamento dos *“speckles”* tem inúmeras vantagens em relação à avaliação bidimensional (2D). Considerando que o miocárdio ventricular esquerdo é composto por três camadas de fibras, que estão dispostas em direções diferentes (longitudinal, circunferencial e transversal), os “*speckles*” acabam por apresentar uma trajetória não linear, fugindo do plano bidimensional da imagem por alguns momentos do ciclo cardíaco. Por mais que se realizem múltiplas aquisições longitudinais e transversais do miocárdio ventricular esquerdo, a avaliação dos “*speckles*” no ciclo cardíaco se faz por interpolação, não sendo tão precisa quanto o 3D, que permite acompanhá-los durante todo o ciclo cardíaco nas múltiplas dimensões. Assim, as medidas do *strain* 3D não são impactadas pela “*out-of-plane*”, pela torsão miocárdica ou encurtamento apical.^410^

Em relação ao VD, a metodologia 3D é a única que permite a análise global de todo o miocárdio da câmara, ao passo que, no 2D, apenas a septo e/ou parede livre é avaliada. Ademais, a avaliação do *strain* 3D é mais fidedigna e fisiológica, pois a análise dos diferentes componentes da deformação miocárdica ocorre de forma simultânea em um único *dataset* ou ciclo cardíaco. Dessa forma, o *strain* 3D é uma aplicação promissora para uma avaliação quantitativa, objetiva, compreensiva e reprodutiva da função mecânica do miocárdio. Contudo, essa metodologia apresenta forte dependência da uma boa janela acústica e de um ritmo cardíaco regular, fatores que são os principais limitantes para a incorporação rotineira e sistemática do *strain* 3D.^411^ A aplicação clínica também é limitada devido a diferenças nos algoritmos para acompanhamento dos “*speckles*” e o cálculo (*cut-off*) da deformação miocárdica, que não está padronizado entre os diferentes fabricantes de *softwares*.^412,413^

Uma vez que as medidas do *strain* 3D obtidas por diferentes fabricantes e *softwares* não são intercambiáveis, a incorporação clínica em avaliações sequenciais requer que as aquisições basais e no acompanhamento do paciente, bem como as análises, sejam obtidas usando o mesmo equipamento, e a interpretação dos resultados deve considerar os valores da normalidade específicos para o equipamento em questão.^410,412^ Os valores de referência da normalidade também diferem entre as metodologias bidimensional e tridimensional, havendo apenas uma correlação modesta entre os valores do *strain* longitudinal. Por fim, ainda se fazem necessárias pesquisas clínicas para avaliar a acurácia e o valor prognóstico do *strain* 3D.

### 18.2. *Strain* Ventricular Esquerdo

O ST3D (ou *strain* tridimensional) apresenta um princípio melhor quando comparado ao ST2D por não ser limitado a um plano de corte e por permitir dados vetorizados em três planos ortogonais para uma análise. Do ponto de vista de implementação do método, sabemos que o *strain* bidimensional precisa de resolução temporal muito elevada (34–50 vps), porque os “*speckles*” ficam pouco tempo (alguns milissegundos) no plano de corte, o que não ocorre no modo tridimensional. Além disso, para o ST3D, o ideal é a obtenção de seis batimentos cardíacos (“*6 beat acquisition*”), com a maior densidade de linhas e com 44 vps na frequência de 2 a 4 Mhz (pois foi o que apresentou maior acurácia quando comparado a RM). Não se recomenda a aquisição com volumes únicos, e o aumento da resolução temporal no equipamento diminui a qualidade da imagem e o *tracking* por reduzir a densidade das linhas.^414^

A factibilidade geral é em torno de 85%, e as limitações para a implementação da técnica são: janela acústica desfavorável, arritmias cardíacas (impedem aquisições em múltiplos batimentos), visualização incompleta dos seguimentos apicais do VE e VD, problemas de *tracking* dos *speckles* nos seguimentos basais (distantes do transdutor) e determinação dos valores de normalidade e de prognóstico clínico.

### 18.3. *Strain* Ventricular Direito

A análise da contração do VD é importante especialmente para entender o mecanismo dessa câmara diante das doenças congênitas e adquiridas. Porém, ao contrário do VE, a estimativa do VD é mais difícil devido à forma complexa que o VD apresenta e devido à parede fina que possui. Apesar disso, imagens de RM e *speckle tracking* pelo ecocardiograma têm sido promissoras na análise ventricular direita. No entanto, os valores obtidos pelo ST3D para o VD ainda não estão bem estabelecidos.^415^ Persistem problemas técnicos para a análise do *strain* 3D quando o objetivo é analisar as câmaras direitas, uma vez que o *software* foi criado para a análise do VE e ainda é adaptado para o VD na maioria das máquinas. Apesar de o *strain* 3D permitir a análise global de todo miocárdio direito, a tecnologia tridimensional para essa câmara ainda está em andamento, não havendo valores de corte bem definidos para o *strain* 3D do VD até o momento.

#### 18.3.1. Aquisição e Análise do *Full-volume* 3D

Usando imagem harmônica, idealmente se obtém quatro batimentos *triggados* na captura. A profundidade deve ser adequada para que somente o VD, suas paredes e o anel tricúspide preencham o volume, e geralmente é o pico sistólico do *strain* que é usado para análise.

## 18.4. *Strain* Atrial Esquerdo

O sistema de ultrassom na avaliação do *strain* 3D do AE (assim como dos ventrículos) é capaz de adquirir os dados volumétricos do átrio em tempo real e pode medir todos os componentes do *strain*. Contudo, o *tracking* em três dimensões é um grande desafio, e a resolução temporal e espacial do 3D é inferior à do 2D, o que torna a análise tridimensional complexa e mais demorada, pois a alta qualidade de imagem é necessária para a aquisição do *strain* 3D.

Outro ponto em discussão, é a variabilidade inter e intraobservador na avaliação da mecânica cardíaca.^414^ Sabe-se que tanto a fase de reservatório como a de conduto e a de bomba atrial podem ser bem analisadas pelo *strain* 2D, e há valores médios de corte relativamente definidos para o *strain* 2D em cada uma dessas fases. Contudo, ainda não temos valores de referência para o *strain* 3D. As principais aplicações do método nessa câmara, em que a análise do *strain* torna-se relevante são: IC com FE preservada,^7^ avaliação das pressões de enchimento intracavitárias,^95^ função atrial do atleta de elite^416^ e CMPs,^417^ mas os estudos corroboram mais os dados do *strain* 2D do que do *strain* 3D.

## 19. O papel da Ressonância e Tomografia Cardíacas na Avaliação do *Strain*

### 19.1. Introdução

A RMC, com sua alta resolução espacial e temporal e natureza não invasiva, tornou-se uma importante modalidade para a avaliação da função global e segmentar dos ventrículos. A avaliação do *strain* é uma medida estabelecida e confiável de quantificação de disfunção contrátil regional e global e possui a capacidade de detectar disfunção cardíaca subclínica sendo, portanto, uma ferramenta útil para a avaliação da função miocárdica. O ecocardiograma é, atualmente, o método mais disponível e de menor custo para a avaliação do *strain,* porém a análise pode ser prejudicada em pacientes com limitação de janela acústica.

### 19.2. Métodos de Aquisição do *Strain* pela Ressonância Magnética Cardíaca

O *tagging* miocárdico (“marcação miocárdica”) é a técnica mais validada em estudos e consiste em uma fase de preparação em que “*tags*” magnéticos (linhas pretas, *tags*) são ortogonalmente sobrepostas ao miocárdio no início de uma sequência de cine.^12,418^ Outra alternativa ao *tagging* que proporciona análise direta do *strain* miocárdico pela RMC são as técnicas de SENC (*strain-encoded)* e DENSE *(displacement encoding with stimulated echoes).*^419^ Recentemente, foi desenvolvido o método de *feature tracking* (FT), que permite a quantificação da deformação do miocárdio nas imagens tradicionais de cine RMC sem a necessidade de aquisições adicionais ou longa análise.^420,421^

Em todas as técnicas de análise do *strain*, os parâmetros de *strain* circunferencial e longitudinal globais foram mais reprodutíveis e consistentes do que os regionais.^422^ Mais detalhes sobre os métodos de aquisição do *strain* pela RMC podem ser obtidos nas referências.^12,418-422^

### 19.3. *Strain* do Ventrículo Direito pela Ressonância Magnética Cardíaca

A medida do *strain* miocárdico é um método preciso e prático de avaliação da função do VD, por se tratar de um marcador mais sensível e precoce de disfunção contrátil do que outros métodos disponíveis, como a FE. Estudos têm demonstrado o potencial do *strain* do VD, avaliado pela RMC, em fornecer informações aditivas e prognósticas independentes.^423-428^ Existem alguns estudos publicados nos quais os autores analisaram o *strain* do VD em indivíduos saudáveis e também em grupos-controle sem cardiopatia.^423,426,429,430^

As patologias que mais acometem o VD, como cardiopatias congênitas, HP e displasia arritmogênica (DAVD) foram as que tiveram maior aplicabilidade na análise do *strain* do VD. O FT–RMC (*feature tracking* pela RMC) foi utilizado em pacientes com tetralogia de Fallot corrigida, e os valores de *strain* estavam reduzidos nesses pacientes e estavam relacionados com parâmetros de função sistólica (FE biventricular) e também capacidade funcional no teste cardiopulmonar.^425^

A avaliação das funções globais e segmentares do VD é fundamental para o diagnóstico multiparamétrico de DAVD, e o *strain* do VD provou ser uma ferramenta extremamente útil.^428,431^ Os *strains* global e segmentar do VD estão significativamente reduzidos em pacientes com DAVD, independente das dimensões e função do VD, de modo que o comprometimento da deformação do VD pode representar um marcador precoce da doença.^428^

**Figura 19.1:**
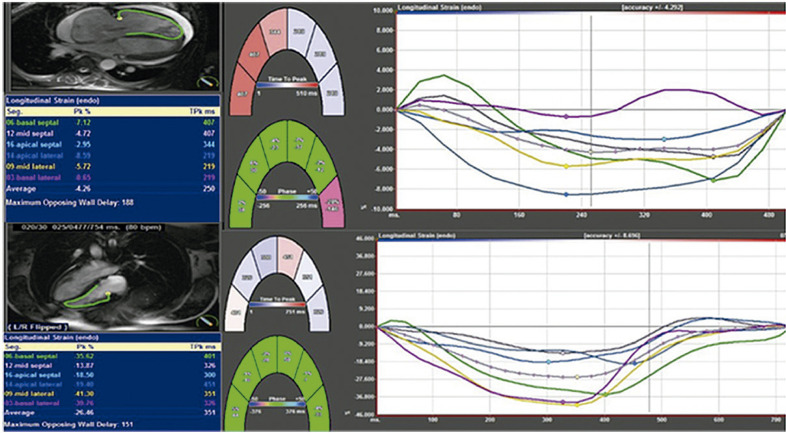
– Análise do strain longitudinal global pelo método de feature tracking em paciente com hipertensão pulmonar (acima) e sem hipertensão pulmonar (abaixo). O paciente com hipertensão pulmonar apresentou strain de 4,26% e o sem hipertensão pulmonar de 26,46%.

A Figura 19.1 apresenta exemplos de *strain* do VD em paciente normal e paciente com HP.

### 19.4. *Strain* do Ventrículo Esquerdo pela Ressonância Magnética Cardíaca

Os valores médios em indivíduos saudáveis dos tipos de *strain* do VE (SCG, SRG, SLG) foram pesquisados na última década através do FT–RMC,^419-423^ incluindo uma importante metanálise.^424^ Os maiores e mais recentes estudos em SCG e SR pelo FT-RMC aplicaram a análise da média de três cortes do eixo curto. A maior parte do SLGVE foi calculada através de um corte 4 câmaras, enquanto algumas publicações mais recentes trazem avaliações da média de três cortes longitudinais. Os valores do SLG e do SCG flutuam dentro de uma margem restrita, enquanto os valores de SRG tiveram intervalos de confiança mais amplos. Especula-se que a movimentação através do plano e a grande variabilidade pessoal podem explicar parcialmente esse fenômeno. Entretanto, a real causa ainda permanece incerta.^424^

Observa-se uma forte relação dos graus de deformação miocárdica com a presença de realce tardio (RT) miocárdico, em especial o SCG, mas também o SLG derivados do FT-RMC. Ademais, observa-se boa correlação entre as técnicas derivadas entre a ecocardiografia e a RMC.^425^

Na CMP dilatada, a presença de um SLG acentuadamente reduzido se relacionou fortemente com pior sobrevida, mesmo naqueles com FE muito reduzida, independentemente da classe funcional e outros achados da RMC.^426^ O FT-RMC pode identificar o subgrupo de portadores de IC com FE preservada e DD através do SLG alterado em comparação a indivíduos saudáveis.^427^

Na diferenciação entre pericardite constritiva (PC) e CMP restritiva, o SLG derivado da RMC apresentou valor diagnóstico similar ao do ecocardiograma, além de apresentar alto valor discriminatório entre essas patologias. Os valores de SLG são significativamente menores na CMP restritiva, enquanto na pericardite são próximos dos valores encontrados em controles normais.^428^ Os componentes longitudinal e circunferencial do *strain* são alterados também em casos de miocardite.^429^

Publicações que exploraram a capacidade do *strain* derivado pela ressonância usando *tagging* miocárdico demonstraram alta capacidade em determinar portadores de amiloidose com presença de RT, sendo potencialmente mais sensível que a própria sequencia pós-contraste.^430^ A perda do gradiente base-ápice do *strain* circunferencial parece representar um achado precoce das alterações observadas na doença de Fabry, já as modalidades de *strain* longitudinal e circunferencial não apresentaram variação significativa entre os controles sadios.^431^

Usando o FT-RMC, foi observado que pacientes com CMPH têm redução do SLG, SRG e SCG comparados com controles sadios, sendo o SLG e o SRG preditores de eventos adversos,^432^ assim como já foi demonstrado que o SLG é significativamente superior em pacientes portadores de HAS do que em portadores de CMPH.^433^

O diagnóstico da doença coronariana isquêmica pela RMC pode ser aperfeiçoado se adicionada a análise com FT-RMC, sendo possível detectar pequenas alterações no *strain* circunferencial após o estresse por dobutamina, podendo o SLG ser útil na detecção de infarto e avaliação de viabilidade.^434^ Os três tipos de *strain* estão reduzidos em pacientes que sofreram IAM com supradesnivelamento do segmento ST, sendo preditores independentes de eventos cardiovasculares adversos.^435^

Pacientes portadores de EA importante têm SLG e SCG reduzidos em relação aos controles sadios, a despeito dos sintomas apresentados.^436^ Em pacientes portadores de valva aórtica bicúspide e FE preservada, foram observados indícios de DD através de alterações no *strain* circunferencial.^437^ A cardiotoxicidade induzida por quimioterapia apresenta anormalidades no SLG e SCG muito antes do declínio da FEVE.^438^

Recentemente, foi reportado que o SLG pelo FT-RMC apresenta associação mais intensa com mortalidade do que os observados pela combinação da FEVE e pelo RT miocárdico. Até o momento, essa foi a maior experiência em avaliar o valor prognóstico do SLG avaliado pelo FT-RMC. Ajustado para fatores de risco clássicos, incluindo FEVE e RT, a piora de 1% no SLG foi associada a um aumento de 89% no risco de morte em pacientes isquêmicos e não isquêmicos.^439^

### 19.5. *Strain* do Átrio Esquerdo pela Ressonância Magnética Cardíaca

A avaliação da função do AE tem sido cada vez mais reconhecida como um fator crucial em uma diversidade de patologias cardíacas. Normalmente, sua alteração está associada a pior prognóstico e precede o estabelecimento de IC.

O AE tem a função de reservatório para a drenagem das veias pulmonares, servindo de conduto para a passagem do fluxo até o VE por diferença de pressão causada pela abertura das cúspides mitrais e por fim de função contrátil, com a sístole atrial ocorrendo no final da diástole do VE.^440^

A análise do *strain* atrial baseada em FT-RMC quantifica de forma confiável o strain longitudinal do AE e o *strain rate*. Usando imagens cine-RM padrão, ela discrimina entre pacientes com relaxamento ventricular esquerdo alterados e pacientes saudáveis, como podemos observar na Tabela 19.1.^441^ Em um subestudo do MESA, a redução no SLG atrial e alteração do volume indexado mínimo do AE foram fatores preditores independentes para a instalação de IC, mesmo ajustados para a massa do VE e pro-BNP.^442^ Da mesma forma, a avaliação da função fásica do AE foi um preditor de risco independente para a admissão por IC ou morte, mesmo após ajustar para o volume do AE e o remodelamento ventricular.^443^

**Tabela 19.1:** – *Strain* do átrio esquerdo

Tipos	ICFEp	CMPH	Normais
Reservatório	16,3 ± 5,8	22,1 ± 5,5	29,1 ± 5,3
Conduto	11,9 ± 4,0	10,4 ± 3,9	21,3 ± 5,1
Contrátil	4,5 ± 2,9	11,7 ± 4,0	7,8 ± 2,5

*CMPH: cardiomiopatia hipertrófica; ICFep: insuficiência cardíaca com fração de ejeção preservada.*

### 19.6. *Strain* pela Tomografia Cardíaca

A avaliação do *strain* pela tomografia cardíaca (TC) pode ser realizada utilizando-se o método *feature tracking* (FT-TC) nas aquisições contrastadas e trigadas, com reconstruções funcionais do ciclo cardíaco. Os dados ainda são escassos, mas a sua aplicabilidade foi testada em algumas publicações recentes em pacientes portadores de EAo importante, submetidos a implante de prótese aórtica transcutânea. Os resultados demonstram valores similares do SLG entre a FT-TC e a derivada pelo ECO,^444,445^ assim como alta reprodutibilidade intraobservador e intraclasse para SLG FT-TC do VE, apesar de aparentemente subestimar os valores.^444^

Outra publicação explorou a relação do *strain* FT-TC com a doença isquêmica do coração em portadores de lesão significativa na artéria descendente anterior. Observou-se uma redução do *strain* longitudinal nos segmentos do território da artéria descendente anterior, a despeito de volumes diastólico, sistólico e FE normais.^446^

No momento, as limitações para a utilização do *strain* pela RMC e TC se devem à escassa disponibilidade e ao elevado custo de *softwares* de pós-processamento.
