# Supplementary material for: Posicionamento do Departamento de Imagem Cardiovascular da Sociedade Brasileira de Cardiologia sobre o Uso do Strain Miocárdico na Rotina do Cardiologista – 2023
Source: Arq Bras Cardiol. 2023 Dec 12;120(12):e20230646. [Article in Portuguese] doi: 10.36660/abc.20230646 (PMC10789373; doi:10.36660/abc.20230646)
Supplement: Translation

# Translation: Position Statement on the Use of Myocardial Strain in Cardiology Routines by the Brazilian Society of Cardiology’s Department Of Cardiovascular Imaging – 2023

**Authors:** André Luiz Cerqueira Almeida, Marcelo Dantas Tavares de Melo, David Costa de Souza Le Bihan, Marcelo Luiz Campos Vieira, José Luiz Barros Pena, José Maria Del Castillo, Henry Abensur, Renato de Aguiar Hortegal, Maria Estefania Bosco Otto, Rafael Bonafim Piveta, Maria Rosa Dantas, Jorge Eduardo Assef, Adenalva Lima de Souza Beck, Thais Harada Campos Espirito Santo, Tonnison de Oliveira Silva, Vera Maria Cury Salemi, Camila Rocon, Márcio Silva Miguel Lima, Silvio Henrique Barberato, Ana Clara Rodrigues, Arnaldo Rabschkowisky, Daniela do Carmo Rassi Frota, Eliza de Almeida Gripp, Rodrigo Bellio de Mattos Barretto, Sandra Marques e Silva, Sanderson Antonio Cauduro, Aurélio Carvalho Pinheiro, Salustiano Pereira de Araujo, Cintia Galhardo Tressino, Carlos Eduardo Suaide Silva, Claudia Gianini Monaco, Marcelo Goulart Paiva, Cláudio Henrique Fisher, Marco Stephan Lofrano Alves, Cláudia R. Pinheiro de Castro Grau, Maria Veronica Camara dos Santos, Isabel Cristina Britto Guimarães, Samira Saady Morhy, Gabriela Nunes Leal, Andressa Mussi Soares, Cecilia Beatriz Bittencourt Viana Cruz, Fabio Villaça Guimarães, Bruna Morhy Borges Leal Assunção, Rafael Modesto Fernandes, Roberto Magalhães Saraiva, Jeane Mike Tsutsui, Fábio Luis de Jesus Soares, Sandra Nívea dos Reis Saraiva Falcão, Viviane Tiemi Hotta, Anderson da Costa Armstrong, Daniel de Andrade Hygidio, Marcelo Haertel Miglioranza, Ana Cristina Camarozano, Marly Maria Uellendahl Lopes, Rodrigo Julio Cerci, Maria Eduarda Menezes de Siqueira, Jorge Andion Torreão, Carlos Eduardo Rochitte, Alex Felix

**Affiliations:** 1 Brazil Santa Casa de Misericórdia de Feira de Santana, Feira de Santana, BA – Brazil; 2 Brazil Universidade Federal da Paraíba, João Pessoa, PB – Brazil; 3 Brazil Instituto do Coração da Faculdade de Medicina da Universidade de São Paulo (Incor/FMUSP), São Paulo, SP – Brazil; 4 Brazil Faculdade Ciências Médicas de Minas Gerais, Belo Horizonte, MG – Brazil; 5 Brazil Hospital Felicio Rocho, Belo Horizonte, MG – Brazil; 6 Brazil Escola de Ecografia de Pernambuco (ECOPE), Recife, PE – Brazil; 7 Brazil Beneficência Portuguesa de São Paulo, São Paulo, SP – Brazil; 8 Brazil Instituto Dante Pazzanese de Cardiologia, São Paulo, SP – Brazil; 9 Brazil DF Star, Rede D’Or São Luiz, Brasília, DF – Brazil; 10 Brazil Hospital Israelita Albert Einstein, São Paulo, SP – Brazil; 11 Brazil Santa Casa de Feira de Santana, Feira de Santana, BA – Brazil; 12 Brazil Instituto de Cardiologia e Transplantes do DF, Brasília, DF – Brazil; 13 Brazil Diagnoson, Grupo Fleury, Salvador, BA – Brazil; 14 Brazil Hospital da Bahia, Salvador, BA – Brazil; 15 Brazil Escola Bahiana de Medicina e Saúde Pública, Salvador, BA – Brazil; 16 Brazil Hospital do Coração (HCor), São Paulo, SP – Brazil; 17 Brazil CardioEco Centro de Diagnóstico Cardiovascular e Ecocardiografia, Curitiba, PR – Brazil; 18 Brazil UnitedHealth Group, Rio de Janeiro, RJ – Brazil; 19 Brazil Faculdade de Medicina da Universidade Federal de Goiás, Goiânia, GO – Brazil; 20 Brazil Hospital Pró-Cardiaco, Rio de Janeiro, RJ – Brazil; 21 Brazil Hospital Universitário Antônio Pedro da Universidade Federal Fluminense (UFF), Rio de Janeiro, RJ – Brazil; 22 Brazil Secretaria de Estado de Saúde do Distrito Federal, Brasília, DF – Brazil; 23 Brazil Clínica Cardio & Saúde, Curitiba, PR – Brazil; 24 Brazil Heart & Mind Care Serviços Médicos (HMC), Manaus, AM – Brazil; 25 Brazil Cardiologia Não Invasiva, Clínica CNI, Uberlândia, MG – Brazil; 26 Brazil Diagnósticos da América SA (DASA), São Paulo, SP – Brazil; 27 Brazil Hospital 9 de Julho, São Paulo, SP – Brazil; 28 Brazil Universidade Federal do Paraná (UFPR), Curitiba, PR – Brazil; 29 Brazil Departamento de Cardiologia Pediátrica (DCC/CP) da Sociedade Brasileira de Cardiologia (SBC), São Paulo, SP – Brazil; 30 Brazil Sociedade Brasileira de Oncologia Pediátrica, São Paulo, SP – Brazil; 31 Brazil Universidade Federal da Bahia (UFBA), Salvador, BA – Brazil; 32 Brazil Instituto da Criança e do Adolescente do Hospital das Clinicas Faculdade de Medicina da Universidade de São Paulo (FMUSP), São Paulo, SP – Brazil; 33 Brazil Hospital Evangélico, Cachoeiro de Itapemirim, ES – Brazil; 34 Brazil Instituto do Coração de Marília (ICM), Marília, SP – Brazil; 35 Brazil Instituto do Câncer do Estado de São Paulo (ICESP), São Paulo, SP – Brazil; 36 Brazil Hospital Aliança Rede D’Or São Luiz, Salvador, BA – Brazil; 37 Brazil Fundação Oswaldo Cruz (FIOCRUZ), Rio de Janeiro, RJ – Brazil; 38 Brazil Grupo Fleury, São Paulo, SP – Brazil; 39 Brazil Hospital Cardio Pulmonar Rede D’or São Luiz, Salvador, BA – Brazil; 40 Brazil Universidade Federal do Ceará, Fortaleza, CE – Brazil; 41 Brazil Universidade Federal do Vale do São Francisco (UNIVASF), Petrolina, PE – Brazil; 42 Brazil Hospital Nossa Senhora da Conceição, Tubarão, SC – Brazil; 43 Brazil Universidade do Sul de Santa Catarina (UNISUL), Tubarão, SC – Brazil; 44 Brazil EcoHaertel - Hospital Mae de Deus, Porto Alegre, RS – Brazil; 45 Brazil Universidade Federal de Ciências da Saúde de Porto Alegre, Porto Alegre, RS – Brazil; 46 Brazil Universidade Federal de São Paulo (UNIFESP), São Paulo, SP – Brazil; 47 Brazil Quanta Diagnóstico por Imagem, Curitiba, PR – Brazil; 48 Brazil Hospital Santa Izabel Salvador, BA – Brazil; 49 Brazil Santa Casa da Bahia, Salvador, BA – Brazil; 50 Brazil Instituto Nacional de Cardiologia (INC), Rio de Janeiro, RJ – Brazil

## Abstract

**Central Illustration:** : Position Statement on the Use of Myocardial Strain in Cardiology Routines by the Brazilian Society of Cardiology’s Department Of Cardiovascular Imaging – 2023

**Table:** 

Position Statement on the Use of Myocardial Strain in Cardiology Routines by the Brazilian Society of Cardiology’s Department Of Cardiovascular Imaging – 2023	
The report below lists declarations of interest as reported to the SBC by the experts during the period of the development of these statement, 2022/2023.	
Expert	Type of relationship with industry
Adenalva Lima de Souza Beck	Nothing to be declared
Alex Felix	Nothing to be declared
Ana Clara Rodrigues	Nothing to be declared
Ana Cristina Camarozano	Nothing to be declared
Anderson da Costa Armstrong	Financial declaration A - Economically relevant payments of any kind made to (i) you, (ii) your spouse/partner or any other person living with you, (iii) any legal person in which any of these is either a direct or indirect controlling owner, business partner, shareholder or participant; any payments received for lectures, lessons, training instruction, compensation, fees paid for participation in advisory boards, investigative boards or other committees, etc. From the brazilian or international pharmaceutical, orthosis, prosthesis, equipment and implants industry: - Amyloidosis: Anylam. Other relationships Funding of continuing medical education activities, including travel, accommodation and registration in conferences and courses, from the brazilian or international pharmaceutical, orthosis, prosthesis, equipment and implants industry: - Health area; CARDIOVASF.
André Luiz Cerqueira de Almeida	Financial declaration A - Economically relevant payments of any kind made to (i) you, (ii) your spouse/partner or any other person living with you, (iii) any legal person in which any of these is either a direct or indirect controlling owner, business partner, shareholder or participant; any payments received for lectures, lessons, training instruction, compensation, fees paid for participation in advisory boards, investigative boards or other committees, etc. From the brazilian or international pharmaceutical, orthosis, prosthesis, equipment and implants industry: - Boston Scientific: Speaker.
Andressa Mussi Soares	Financial declaration A - Economically relevant payments of any kind made to (i) you, (ii) your spouse/partner or any other person living with you, (iii) any legal person in which any of these is either a direct or indirect controlling owner, business partner, shareholder or participant; any payments received for lectures, lessons, training instruction, compensation, fees paid for participation in advisory boards, investigative boards or other committees, etc. From the brazilian or international pharmaceutical, orthosis, prosthesis, equipment and implants industry: - Bayer: Anticoagulation and heart failure; Pfizer: Anticoagulation and amyloidosis; Jannsen: Leukemia. Other relationships Funding of continuing medical education activities, including travel, accommodation and registration in conferences and courses, from the brazilian or international pharmaceutical, orthosis, prosthesis, equipment and implants industry: - Bayer: Heart failure.
Arnaldo Rabischoffsky	Nothing to be declared
Aurélio Carvalho Pinheiro	Nothing to be declared
Bruna Morhy Borges Leal Assunção	Nothing to be declared
Camila Rocon	Nothing to be declared
Carlos Eduardo Suaide Silva	Nothing to be declared
Carlos Eduardo Rochitte	Nothing to be declared
Cecilia Beatriz Bittencourt Viana Cruz	Nothing to be declared
Cintia Galhardo Tressino	Nothing to be declared
Claudia Gianini Monaco	Nothing to be declared
Claudia R. Pinheiro de Castro Grau	Nothing to be declared
Cláudio Henrique Fischer	Nothing to be declared
Daniel de Andrade Hygidio	Nothing to be declared
Daniela do Carmo Rassi Frota	Nothing to be declared
David Costa de Souza Le Bihan	Financial declaration A - Economically relevant payments of any kind made to (i) you, (ii) your spouse/partner or any other person living with you, (iii) any legal person in which any of these is either a direct or indirect controlling owner, business partner, shareholder or participant; any payments received for lectures, lessons, training instruction, compensation, fees paid for participation in advisory boards, investigative boards or other committees, etc. From the brazilian or international pharmaceutical, orthosis, prosthesis, equipment and implants industry: - Edwards Lifescience; Abbott; GE Healthcare; Philips.
Eliza de Almeida Gripp	Nothing to be declared
Fábio Luis de Jesus Soares	Nothing to be declared
Fabio Villaça Guimarães Filho	Nothing to be declared
Gabriela Nunes Leal	Nothing to be declared
Henry Abensur	Nothing to be declared
Isabel Cristina Britto Guimarães	Nothing to be declared
Jeane Mike Tsutsui	Other relationships Any economically relevant equity interest in companies in the healthcare or education industry or in any companies competing with or supplying to SBC: - Health area: Grupo Fleury.
Jorge Andion Torreão	Other relationships Any economically relevant equity interest in companies in the healthcare or education industry or in any companies competing with or supplying to SBC: - Partner of an education company in the health sector.
Jorge Eduardo Assef	Nothing to be declared
José Luiz Barros Pena	Nothing to be declared
Jose Maria Del Castillo	Nothing to be declared
Marcelo Dantas Tavares de Melo	Nothing to be declared
Marcelo Goulart Paiva	Nothing to be declared
Marcelo Haertel Miglioranza	Nothing to be declared
Marcelo Luiz Campos Vieira	Nothing to be declared
Márcio Silva Miguel Lima	Nothing to be declared
Marco Stephan Lofrano Alves	Nothing to be declared
Maria Eduarda Menezes de Siqueira	Nothing to be declared
Maria Estefânia Bosco Otto	Nothing to be declared
Maria Rosa Dantas	Nothing to be declared
Maria Veronica Camara dos Santos	Nothing to be declared
Marly Maria Uellendahl Lopes	Nothing to be declared
Rafael Bonafim Piveta	Other relationships Any economically relevant equity interest in companies in the healthcare or education industry or in any companies competing with or supplying to SBC: - Partner at WavesMed (digital platform for continuing education/updates).
Rafael Modesto Fernandes	Nothing to be declared
Renato de Aguiar Hortegal	Nothing to be declared
Roberto Magalhães Saraiva	Nothing to be declared
Rodrigo Bellio de Mattos Barretto	Financial declaration A - Economically relevant payments of any kind made to (i) you, (ii) your spouse/partner or any other person living with you, (iii) any legal person in which any of these is either a direct or indirect controlling owner, business partner, shareholder or participant; any payments received for lectures, lessons, training instruction, compensation, fees paid for participation in advisory boards, investigative boards or other committees, etc. From the brazilian or international pharmaceutical, orthosis, prosthesis, equipment and implants industry: - GE; Abbot; Edwards.
Rodrigo Julio Cerci	Nothing to be declared
Salustiano Pereira de Araujo	Nothing to be declared
Samira Saady Morhy	Nothing to be declared
Sanderson Antonio Cauduro	Nothing to be declared
Sandra Marques e Silva	Other relationships Funding of continuing medical education activities, including travel, accommodation and registration in conferences and courses, from the brazilian or international pharmaceutical, orthosis, prosthesis, equipment and implants industry: - Pfizer: Amyloidosis; Sanofi, Pint Pharma, Takeda and Chiesi: Fabry.
Sandra Nívea dos Reis Saraiva Falcão	Nothing to be declared
Silvio Henrique Barberato	Nothing to be declared
Thais Harada Campos Espirito Santo	Nothing to be declared
Tonnison de Oliveira Silva	Nothing to be declared
Vera Maria Cury Salemi	Nothing to be declared
Viviane Tiemi Hotta	Financial declaration B - Research funding under your direct/personal responsibility (directed to the department or institution) from the Brazilian or international pharmaceutical, orthosis, prosthesis, equipment and implants industry: - Pfizer: Amyloidosis.

## Table of contents

1. Basic Concepts in Left Ventricular Strain 7

1.1. Brief Introduction to the Physical Principles of Speckle Formation in Cardiovascular Imaging 7

1.2. Definitions 7

**1.2.1. Strain and Strain Rate** 7

**1.2.2. Longitudinal, Circumferential, and Radial Deformation** 7

**1.2.3. Timing Mechanical Events** 8

**1.2.4. Peak Measurements Extracted from Strain Curves** 8

1.3. Factors Affecting Strain Estimation 8

**1.3.1 Image Quality** 8

**1.3.2. Cardiovascular Imaging Modality** 8

**1.3.3. Software Manufacturer and Version** 8

**1.3.4. Hemodynamic Conditions** 8

1.4. Global Longitudinal Strain 9

2. General Recommendations for Using Strain: Clinical Applicability, Comparison with Ejection Fraction, and Adequate Description for Echocardiography Reports 10

2.1. Prognostic Value, Parametric Patterns, and Subclinical Detection of Heart Disease Through Myocardial Strain 10

2.2. Which is Better, Strain or Ejection Fraction? 11

2.3. General Recommendations for Reporting Strain Results and Normality Values 12

2.4. Conclusion 12

3. Strain in Cardio-oncology 13

4. Strain in Diastolic Dysfunction 15

4.1. Introduction 15

4.2. Left Ventricle Strain 15

4.3. Left Atrial Strain 15

4.4. Conclusion 16

5. Strain in Cardiomyopathies 16

5.1. Introduction 16

5.2. Dilated Cardiomyopathy 16

5.3. Arrhythmogenic Cardiomyopathy 16

5.4. Hypertrophic Cardiomyopathy 17

5.5. Endomyocardial Fibrosis 17

5.6. Noncompacted Myocardium 17

6. Strain in Valvular Heart Disease 18

7. Strain in Ischemic Heart Disease 19

7.1. Introduction 19

7.2. Strain in Acute Coronary Syndrome 19

7.3. Strain in Chronic Coronary Syndromes 20

7.4. Right Ventricular Strain in Ischemic Heart Disease 20

8. Strain Assessment in Systemic Diseases (Amyloidosis and Fabry Disease) 20

8.1. Strain Assessment in Cardiac Amyloidosis 20

**8.1.1. Myocardial Strain Assessment in the Diagnosis of Cardiac Amyloidosis** 21

8.2. Fabry Disease 23

9. Strain in Hypertension 24

9.1. Introduction 24

9.2. Hypertension without Left Ventricular Hypertrophy Criteria 24

9.3. Hypertension with Left Ventricular Hypertrophy Criteria 25

9.4. Clinical Treatment 25

9.5. Conclusion 25

10. Strain in Athletes 25

11. Strain in Stress Echocardiography 26

12. Strain in Congenital Heart Disease 26

13. Right Ventricle Strain 26

13.1. Introduction 26

13.2. Anatomical and Functional Characteristics of the Right Ventricle 28

13.3. Right Ventricle and Echocardiographic Parameters in Systolic Function Assessment 28

13.4. Acquisition and Limitations 28

13.5. Indications/Normal Values 30

14. Left and Right Atrial Strain 31

14.1. Left Atrial Strain Assessment Techniques 31

14.2. Normality Values 31

14.3. Clinical Applicability of Left Atrial Strain 32

**14.3.1. Left Atrial Strain and Diastolic Function in Heart Failure** 32

**14.3.2. Atrial Fibrillation** 32

**14.3.3. Valvular Heart Disease** 32

**14.3.4. Coronary Artery Disease** 32

14.4. Right Atrial Strain 32

15. Assessing Left Ventricular Torsion 32

15.1. Introduction 32

15.2. Definitions and Nomenclature 33

15.3. Step-by-step Assessment of Ventricular Torsion by Speckle Tracking Echocardiography 33

16. Strain in Ventricular Dyssynchrony Analysis 34

16.1. Introduction 34

16.2. Dyssynchrony Assessment when Selecting Patients for Cardiac Resynchronization Therapy 35

16.3. Myocardial Viability Assessment 35

16.4. Electrode Implantation Site 35

16.5. Prognostic Assessment after CRT 35

16.6. Adjustment of Resynchronization Parameters 36

17. Myocardial Work 36

**17.1. Introduction** 36

**17.2. Calculating Myocardial Work** 36

**17.3. Normality Values** 37

**17.4. Potential Clinical Use** 37

18. Three-dimensional Strain Assessment: What can be Added 40

18.1. Introduction 40

18.2. Left Ventricular Strain 41

18.3. Right Ventricular Strain 41

**18.3.1. Full-volume 3D Acquisition and Analysis** 41

18.4. Left Atrial Strain 41

19. The Role of Cardiac Resonance and Tomography in Strain Assessment 41

19.1. Introduction 41

19.2. Strain Acquisition Methods by Cardiac Magnetic Resonance Imaging 41

19.3. Determining Right Ventricle Strain Through Cardiac Magnetic Resonance Imaging 42

19.4. Determining Left Ventricular Strain Through Cardiac Magnetic Resonance Imaging 42

19.5. Determining Left Atrial Strain Through Cardiac Magnetic Resonance Imaging 42

19.6. Determining Strain Through Cardiac Tomography 43

References 43

## 1. Basic Concepts in Left Ventricular Strain

### 1.1. Brief Introduction to the Physical Principles of Speckle Formation in Cardiovascular Imaging

Optical coherence imaging systems such as laser, optical coherence tomography, or ultrasound produce grainy reflections known as speckle.^1,2^ In echocardiography, an emitted ultrasound pulse moves in a straight line, interacting with the acoustic interfaces of the thoracic cavity until it reaches the heart. Some of the beam is reflected by different cardiac structures, producing an echo that is partially captured by the transducer, and the software uses it as an input to produce images. In this case, the beam’s wavelength is usually shorter than the structures that reflect it.

However, when the wavelength is longer than the microstructures it interacts with, the beam is a scattered, radiating in all directions (diffuse scattering). This phenomenon is the result of an interference pattern in all wavefronts scattered by the different phenomena (eg, local differences in tissue density and compressibility). Some this diffuse scattering, or speckle, is captured by the transducer. Although speckle makes the B-mode image less clear for human operators, it should not be seen as noise, since it carries unique information, acting as a myocardial “fingerprint”.^1^

### 1.2. Definitions

#### 1.2.1. Strain and Strain Rate

Strain is the amount that an object deforms compared to its original shape.^3^ In cardiology, this concept is represented as the magnitude (%) of myocardial fiber contraction/relaxation in relation to its initial measurement. This concept can be applied to a single segment (regional strain) or to entire heart chambers (global strain), such as the left ventricle (LV). Strain rate indicates the rate of myocardial deformation (%) every second (s^-1^), ie, the speed at which deformation occurs.^3-4^

#### 1.2.2. Longitudinal, Circumferential, and Radial Deformation

Through the concept of strain, the contraction/relaxation of the LV myocardium from its orientation in different axes can be studied. In fact, due to the helical arrangement of cardiac muscle fibers, LV systolic shortening is determined by the longitudinal and circumferential action of fibers,^5^ the two active force vectors of strain (Figure 1.1 A).

**Figure 1.1:** – Myocardial strain in different axes. A) Strain can be measured in the longitudinal, circumferential, and radial directions.*4* A third vector results from the interaction of 2 of these force-vectors. B) Radial-circumferential shear strain. C) Longitudinal-circumferential shear strain (ie, ventricular twist/torsion). D) Radial-circumferential shear strain.

When longitudinal and circumferential forces are applied to a material with low compressibility, such as myocardial tissue, the myocardium is thickened radially (passive deformation component).^6^ Ultimately, this accounts for radial shrinkage of the ventricular cavity.^4^The deformation process is much more complex than we can measure, since for each interaction between the force vectors, a new vector arises from the shear between the different deformations, called shear strain (Figure 1.1 B, C and D).

Systolic fiber shortening/thinning in the longitudinal and circumferential direction produces negative strain values, whereas systolic thickening results in positive strain values. Many authors express only the absolute value (modulus value), and we will do the same herein.

#### 1.2.3. Timing Mechanical Events

Some fundamental definitions for clinical practice are provided below:^1,7^

**•End-systole:** The point of aortic valve closure. Potential substitutes: peak global strain or the volume curve. The software should show which criterion is used to determine end-systole.
**•End-diastole**: The point at which the QRS complex peaks. Event timing should be performed using Doppler imaging in reference to the electrocardiogram.

#### 1.2.4. Peak Measurements Extracted from Strain Curves (Figure 1.2)

**•End-systolic strain:** The point of the strain curve at end-systole, as defined above (aortic valve closure). This is the standard parameter for describing myocardial deformation.
**•Peak systolic strain:** The point at which the peak of the strain curve occurs during systole.
**•Positive peak systolic strain:** the highest positive value of local myocardial stretching at some point during systole.
**•Peak strain:** the highest point of the strain curve during the entire heart cycle. Although this point is usually reached before aortic valve closure, when it occurs afterwards, it is described as post-systolic strain^8^ or post-systolic shortening. Post-systolic strain is the deformation of segments after aortic valve closure that does not contribute to ventricular ejection.

**Figure 1.2:** – Peak measurements derived from strain curves A) Peak systolic strain, peak strain, and end-systolic (ES) strain coincide upon aortic valve closure (AVC). B) Peak systolic strain and peak strain coincide, although both occur immediately before AVC (red bar), resulting in slight dissociation between these and ES strain. C) Peak and end-systolic strain coincide (both with lower absolute values), although peak strain occurs after AVC (post-systolic shortening phenomenon).

## 1.3. Factors Affecting Strain Estimation

### 1.3.1 Image Quality

Image quality is a critical factor in any software that estimates myocardial strain. Several authors have reported the sensitivity of strain and strain rate estimation proportional to the quality of the image and the tracking algorithm.^9-11^

### 1.3.2. Cardiovascular Imaging Modality

Different cardiovascular imaging modalities provide different strain values. Tee et al.^12^ reported differences between transthoracic echocardiography, computed tomography, and cardiac magnetic resonance imaging (MRI).

### 1.3.3. Software Manufacturer and Version

Two studies by the European Association of Cardiovascular Imaging and the American Society of Echocardiography tested the variability of global longitudinal strain (GLS) measurements among different equipment types and software, finding significant divergences.^13-14^ However, such differences are smaller than the ejection fraction (EF) variability reported in the literature.^9-10,15^ In addition to variability among manufacturers, measures can vary in different programs by the same manufacturer, with significant changes in GLS having been reported.^11,15^

Thus, serial echocardiographic studies should ideally be performed using the same device and software and under similar hemodynamic conditions, especially when GLS variation in can restrict therapy options, such as when assessing chemotherapy-induced cardiotoxicity.^4^

### 1.3.4. Hemodynamic Conditions

LV deformation varies considerably according to the ventricle’s preload and afterload conditions.

## 1.4. Global Longitudinal Strain

The evidence is more robust for GLS than for any other cardiac strain parameter, and it is the most relevant type for clinical practice.^9^ It reflects relative LS (%) in the LV myocardium, which occurs from the isovolumetric contraction until the end of the ejection period.^1,5,15^

Mathematically, the contraction at any instant is computed using the algorithm: GLS(t)=100[L(t)−L(ED)/L(ED)] , in which L(t) is the longitudinal length at time t, with L(ED) being the end-diastolic length.^1^

Programs vary significantly regarding L(ED) length: the entire line of the region of interest x the mean of a given number of points in a region of interest x the mean value in each segment of the same frame. The normal GLS value is approximately 20%,^9^ although there is evidence that normality varies according to sex and age.^7^

To analyze LVGLS by speckle tracking, a series of image acquisition precautions are necessary:

1) The patient must be monitored electrocardiographically.
2) If possible, an attempt should be made to achieve expiratory apnea, avoiding translational movements of the heart with respiratory incursions.
3) Balance should be sought between the echocardiographic method’s spatial and temporal resolution (focus, depth, and width adjustments) to optimize the cardiac chamber of interest vs frame rate. The latter should be kept between 40-80 frames per second in patients with a normal heart rate. The higher the heart rate, the higher the required RR values.
4) Avoid foreshortening in LV images.
5) Four-, 3-, and 2-chamber apical acoustic windows must be acquired, preferably with a minimum of 3 beats, excluding extrasystoles.

Table 1.1 and Figure 1.3 summarize the GLS measurement procedure.

**Table 1.1:** – Step-by-step measurement of global longitudinal strain for most manufacturers4

•Good quality cardiac electrocardiographic monitoring
•Obtain 3-, 4-, and 2-chamber acoustic windows with a frame rate between 40-80 frames per second.
•Mark aortic valve closure.
•Mark topographic definitions to determine the region of interest in the 3-, 4- and 2-chamber windows.
•Accept or discard the myocardial segments tracked in each window and make adjustments if necessary.
•Evaluate the curves and interpret the results of the polar map.
•Properly record the hemodynamic conditions under which GLS was measured.

**Figure 1.3:** – Step by step to obtain the global longitudinal strain. Initially, images are acquired in 3, 4 and 2 chambers, with good quality EKG. Images must be acquired at an adequate frame rate (40-80 frames per second) (A: blue ovoid). Aortic valve closure (AVC) is marked using pulsed or continuous Doppler tracking (B). Three points (2 at the base and 1 at the apex) are then marked on the 3 acquired images, determining whether the software adequately tracked the 2D images (C and D). Finally, the curves (E), bull’s eye map (F), and global longitudinal strain values are obtained. Adapted from Tressino et al.*4*

## 2. General Recommendations for Using Strain: Clinical Applicability, Comparison with Ejection Fraction, and Adequate Description for Echocardiography Reports

### 2.1. Prognostic Value, Parametric Patterns, and Subclinical Detection of Heart Disease Through Myocardial Strain

Myocardial strain analysis is a robust and versatile tool that offers additional information with less variability than normal prognosis parameters, in addition to parametric cardiomyopathy patterns, and subclinical lesion detection.

Recent studies have shown the increasing value of LVGLS in relation to left ventricular ejection fraction (LVEF).^10^ The inter- and intraobserver variability of strain analysis is 4.9-8.6%, which is much lower than LVEF, probably because it is less influenced by ventricular preload and afterload.^13,16^ LVGLS is superior to LVEF in patients with heart failure (HF) and in those with reduced and preserved EF (HFrEF and HFpEF, respectively).^17,18^ In addition to LV analysis, worsening right ventricular (RV) strain provides additive prognostic value in patients with HFpEF.^19^

The morphological findings of cardiomyopathies usually overlap, which is a major diagnostic challenge in daily clinical practice. Increased ventricular mass and thickness is common, being associated with diastolic dysfunction (DD) and preserved LVEF in earlier stages. Parametric analysis of LVGLS with polar mapping can reveal some diagnoses in echocardiogram examination that are undetectable through normal parameters, having been described as a fingerprint for some. One example is the apical sparing pattern in amyloidosis, which will be described below in a specific chapter.^20^ Such phenotypic characterization has been met with enthusiasm since it facilitates diagnosis of rare pathologies. However, if strain data are not combined with the patient’s clinical history and morphological and hemodynamic aspects, diagnostic error can result. See examples of apical sparing in Figure 2.1.

**Figure 2.1:** – Global longitudinal strain with apical sparing patterns in different heart diseases. 1: Anthracycline cardiotoxicity; 2: Non-compacted myocardium; 3: Hypothyroidism; 4: Transthyretin amyloidosis.

Strain’s utility as a diagnostic and prognostic tool was consolidated through application in cardio-oncology. This is currently the only area of clinical practice in which it guides conduct (ie, variation from baseline values during chemotherapy). When a relative reduction > 15% occurs, cardiotoxicity with subclinical myocardial injury is considered to have occurred.^21^ In 2019, a group of societies developed criteria for the proper use of different imaging modalities to evaluate cardiac structures in non-valvular heart disease. Of their 81 recommendations, only 4 dealt with the adequate use of strain: 3 in cardio-oncology and 1 in hypertrophic cardiomyopathy (HCM).^22^ Although well-designed studies have not validated strain for other applications, it is widely used in large cardio-oncology centers and was reinforced in a recent update of the Brazilian Cardio-oncology Guidelines.^23^

### 2.2. Which is Better, Strain or Ejection Fraction?

LVEF is one of the main echocardiographic parameters for assessing ventricular function in daily practice, since the data are easy to interpret and are widely accessible through basic ultrasound equipment. This parameter has been extensively validated for heart disease, having being used as a patient inclusion criterion in large therapeutic-intervention studies, as well as a parameter for evaluating and monitoring the results.^24^ The prognostic value of LVEF has been well established in chronic HF,^25^ and the European Society of Cardiology’s current recommendation is for HF to be classified according to the following LVEF values: **1)** HFpEF: EF ≥ 50%; **2)** HF with a moderately reduced EF (40-49%); and **3)** HFrEF: EF < 40%).^26^

LVEF is an important quantitative parameter for defining specific strategies in HF, eg, it is used to indicate cardiac resynchronization therapy in patients with refractory HF (LVEF ≤ 35%) or to detect cardiotoxicity in cancer patients who are using anthracyclines (≥ 10% drop in LVEF compared to baseline and values below the lower limit of normality).^27^ However, its accuracy is limited when estimated with the 2D Simpson method due to high interobserver variability, which can reach up to 13%.^28^ Unlike the 2D Simpson method, 3D echocardiography is not based on geometric assumptions and directly measures cavity volumes and LVEF. Its results are quite comparable to those obtained by cardiac MRI. Since it involves automatic algorithms and a lower susceptibility to variation in the acquisition windows (orientation of the apical slices), 3D echocardiography has less intra- and interobserver variability than the 2D method (0.4 [SD, 4.5%]),^29^ making it a good alternative for monitoring patients who have ventricular dysfunction or are at risk of myocardial damage.

Myocardial deformation assessment techniques, such as strain, consider the 3 directions of myocardial fiber contraction: longitudinal, radial, and circumferential. LVEF is primarily determined by the radial and circumferential components, which result in thickening of the myocardial walls and reduction of the ventricular cavity in systole. However, LVEF (ie, the “ejective” function) is not the only determinant of ventricular performance: it also depends on adequate LV end-diastolic volume for normal systolic volume. This is why patients with cardiomyopathies involving phenotypic expression of concentric parietal hypertrophy (eg, infiltrative or hypertrophic) can have normal LVEF and low cardiac output. These patients clinically present as HFpEF and, despite normal LVEF, generally have a worse prognosis than patients with normal LVEF and preserved cardiac output due to changes in contractile function detectable only by GLS.^30^

In fact, longitudinal deformation is the earliest myocardial contractility component to change in most cardiomyopathies and may signal the process at an initial and subclinical stage (prior to reduced LVEF), when therapeutic or cardioprotective measures could have better results. GLS may even be altered in genetic diseases that are not phenotypically expressed, such as Friedreich’s ataxia, which presents with normal mass and LVEF, and might even predict reduced LVEF and its prognosis.^31^

The prognostic value of GLS has been demonstrated in patients with HF, facilitating the prognostic effect of LVEF, especially in patients with EF > 35%.^32^ For this reason, Potter et al. suggested a new classification of ventricular function, complimenting LVEF with LVGLS to facilitate clinical decision-making and prognostic evaluation, especially in patients with LVEF > 53% (HFpEF).^10^

### 2.3. General Recommendations for Reporting Strain Results and Normality Values

To simplify description in echocardiography reports, the type of strain (which defines the contraction or relaxation movements) should be indicated, as well as its absolute values (mainly in sequential comparative studies) to prevent mistaken interpretations of worsening strain. The patient’s vital signs (blood pressure and heart rate) should also be included, due to preload and afterload changes that influence the overall strain value, in addition to the brand of ultrasound equipment and software version, due to varying normality parameters between the manufacturers.^33,34^Table 2.1 lists essential information to be included in a complete strain report.^9^

**Table 2.1:** – Essential elements for describing strain in echocardiography reports

Relevant Strain Information	Description
Vital signs	Blood pressure and heart rate ^16^
*Strain type*	Longitudinal, circumferential, and radial
*Absolute strain value*	For describing chamber function
Cardiac chamber	LV, RV, or LA
Polar map pattern (Figure 2.2)	Any typical patterns, such as amyloidosis or hypertrophic cardiomyopathy ^40^
Variation (%) in sequential exams	Proven use in cardio-oncology. ∆%= baseline strain examination (current/baseline)^21^
Equipment and software version	Normal strain values vary according to equipment brand and version^37^

*∆%: percentage (delta) change; LA: left atrium; LV: left ventricle; RV: right ventricle.*

**Figure 2.2:** – Strain pattern in polar mapping: A: Typical amyloidosis pattern (apical sparing); B: Typical hypertrophic cardiomyopathy pattern with apical predominance (lower strain predominates in the apex, where hypertrophy was more pronounced in 2D assessment).

Normal strain reference values^9,35-39^ must be included in the report. Table 2.2 describes simplified average normality values for different strain types, as well as the degree of use in clinical practice. Unlike LVEF, strain normality values have not yet been consistently assimilated in clinical cardiology and, thus, they must be included in the report as a reference.

**Table 2.2:** – General normality values for different strain and cardiac chamber modalities and applicaiton in clinical practice

Chamber/strain type	Normality value (absolute value)	Application in clinical practice
**LV** Longitudinal (negative) Radial (positive) Circumferential (negative)	18%^35,39^ 20%^35^ 40%^35^ 20%	++++ + +
**RV** Longitudinal free wall (negative)	20%^35,36^	+++
**LA** (varies with age, positive) Reservoir (most common) Conduit Pump	39%^38,39^ 23%^38^ 17%^38,41^	+++ + +

*LA: left atrium; LV: left ventricle; RV: right ventricle. ++++ Commonly used; +++ Used; ++ Limited use in clinical practice; + Limited use in clinical practice and not included in echocardiography equipment software.*

### 2.4. Conclusion

Although current evidence for incorporating strain into daily clinical practice is robust, there are still several challenges to doing so in Brazil, such as unequal access to echocardiogram services with analysis software and a lack of national population data. We used values extrapolated from a population with a very different sociodemographic profile, with application adapted for the Brazilian population. The Brazilian Cardiology Society’s Department of Cardiovascular Imaging is promoting a multicentric work (already in progress), when echocardiographic data from healthy Brazilians is being analyzed. Strain adds to normal echocardiogram values, increasing prognostic robustness, enabling the diagnosis of cardiomyopathies (especially those presenting with increased myocardial thickness) and subclinical myocardial injury.

## 3. Strain in Cardio-oncology

Cancer treatment-related cardiac dysfunction is an important cause of morbidity and mortality.^42,43^ This complication can interrupt treatment and compromise healing and/or adequate cancer control.^44,45^HF due to the cardiotoxicity of chemotherapy drugs often has a worse prognosis than many neoplasms, with a 2-year mortality of up to 60%.^42^ Early identification of cardiotoxicity and cardioprotective intervention could affect prognosis in such cases.^46,47^ However, normal diagnostic methods, such as the 2D LVEF, have low sensitivity.^48,49^ Thus, earlier markers, such as strain analysis, could be of great importance.

Diagnostic imaging methods play a fundamental role in such circumstances, and echocardiography has been the most common tool due to its anatomical correspondence, non-invasive nature, easy access, low cost, and lack of ionizing radiation.^27^ Two-dimensional LVEF, the most common parameter for diagnosing cardiotoxicity, is calculated using Simpson’s method.^21^ However, 3D echocardiography, when available, is the technique of choice for monitoring LVEF in cancer patients. Its main advantages include greater accuracy in recognizing LVEF values below the lower limit of normality and greater reproducibility than the 2D technique, with accuracy similar to cardiac MRI. However, its low availability, high cost, and learning curve prevent more widespread use.^27,50^

Cancer treatment-related ventricular dysfunction is defined as a > 10% absolute drop in LVEF to < 50%, with or without HF symptoms. This echocardiographic examination should be repeated within 2-3 weeks to assess the effects of preload and afterload on LVEF.

Despite being an important and established prognostic factor, LVEF has low sensitivity for diagnosing cardiotoxicity. LVEF is dependent on certain factors, such as cardiac preload, image quality, and examiner experience. It can underestimate real cardiac damage, since compensatory hemodynamic mechanisms allow adequate LV systolic performance despite myocyte dysfunction.^48^ Thus, reduced LVEF often occurs at a very late stage, when therapeutic intervention does not lead to functional recovery in most cases.^46,48,49^

When cardiotoxicity is detected and treated early, patients have a greater chance of recovering ventricular function,^46,51^ and myocardial strain analysis can play an important role in this. Determining strain through 2D speckle-tracking has emerged as a sensitive and reproducible means of analyzing systolic function and LV contractility; this method has been validated in both in vitro and in vivo models*.*^52,53^ A growing number of publications have shown that myocardial strain analysis through 2D speckle-tracking is useful for early and subclinical detection of chemotherapy-induced cardiotoxicity, especially regarding relative decreases in GLS.^23,54-57^

GLS analysis is recommended for patients who undergo potentially cardiotoxic chemotherapy treatment. A ≥ 12% decrease in GLS in relation to baseline suggests a subclinical diagnosis of cardiotoxicity.^21,27^Without baseline (pre-chemotherapy) values for comparison, expert opinion suggests an absolute strain value of < 17% as a marker of subclinical cardiotoxicity, provided there is no other underlying myocardial disease. GLS reductions < 8% from baseline are considered non-significant. Figure 3.1 presents an example of subclinical cardiotoxicity suggested by relative GLS reduction.

**Figure 3.1:** – Example of subclinical cardiotoxicity in a patient with breast cancer. Analysis of LVEF through Simpson’s method and GLS with bull’s eye mapping. A and B: pre-chemotherapy evaluation; B and C: evaluation after a cumulative dose of 240 mg/m2 of doxorubicin. Although there was no significant reduction in LVEF, there was a 22% relative decrease in GLS. LVEF: left ventricular ejection fraction; GLS: global longitudinal strain.

Figure 3.2 presents an algorithm for echocardiographic follow-up in cancer patients based on LVEF and GLS. Figures 3.3 and 3.4 present echocardiographic monitoring data in patients treated with anthracyclines and trastuzumab, respectively.

**Figure 3.2:** – Algorithm for evaluating cancer patients during chemotherapy treatment, based on LVEF and GLS. ACE: angiotensin-converting enzyme; CV: cardiovascular; CT: chemotherapy Echo: echocardiography; GLS: global longitudinal strain; HF: heart failure; LVEF: left ventricular ejection fraction.

**Figure 3.3:** – Echocardiographic monitoring during anthracycline therapy. Echo: echocardiography; GLS: global longitudinal strain; LVEF: left ventricular ejection fraction.

**Figure 3.4:** – Echocardiographic monitoring during trastuzumab therapy. Echo: echocardiography. GLS: global longitudinal strain; LVEF: left ventricular ejection fraction.

SUCCOUR was the first prospective multicenter trial to show the prognostic impact of GLS-based cardioprotection compared to LVEF (3D echocardiography)-based cardioprotection. In patients receiving anthracycline-based chemotherapy (ie, at high risk of cardiotoxicity), cardioprotective intervention (ACE inhibitors and beta-blockers) based on a ≥ 12% reduction in GLS relative to baseline resulted in smaller LVEF reductions and a lower incidence of cancer treatment-related cardiac dysfunction over 1 year of follow-up.^58^

Compared to 2D speckle tracking, calculating strain through 3D tracking has shown technical advantages regarding accuracy, reproducibility, and applicability in different contexts.^59-62^ Recently, small studies have demonstrated the impact of 3D speckle-tracking on early recognition of chemotherapy-related mechanical changes.^63-66^ However, larger studies with longer follow-up are needed to assess this technique’s prognostic value.

Nevertheless, GLS analysis has certain limitations, among which we highlight measurement variability among equipment manufacturers. Thus, measurements must always be performed on the same devices. Like LVEF, GLS is influenced by preload and afterload effects, ventricular geometry, tissue changes (eg, infarction and myocarditis) and conduction disorders. Finally, certain clinical and oncological information is essential and must be clear in the echocardiography report for accurate interpretation, as shown in the Table 3.1.

**Table 3.1:** – Relevant Clinical and Oncological Information in Echocardiography Reports

Time between chemotherapy drug infusion and echocardiography (pre- or post-)
Heart rate Blood pressure Volume status (description of the diameter and variation of the inferior vena cava)
Comparison with baseline, especially left ventricular ejection fraction and GLS: if there is a relative decrease, report the percentage)
Equipment/software used for GLS analysis

*GLS: global longitudinval strain.*

## 4. Strain in Diastolic Dysfunction

### 4.1. Introduction

DD is considered an early marker of myocardial damage and, even when asymptomatic, it has high mortality rates. As DD increases, LV filling pressures and HFpEF increase.^67,68^ The latter accounts for more than 50% of HF hospitalizations, with mortality rates comparable to those of HFrEF.^69^ Unlike HFpEF, preclinical DD may be reversible. However, its pathophysiology is complex and, despite involving several parameters, the currently recommended algorithm is not very sensitive for subclinical stages.^70,71^

Indeterminate cases are also frequent since these parameters do not always change simultaneously or linearly.^70^ Non-diastolic factors can also contribute to HFpEF, leading to varied phenotypic expression depending on the predominant pathophysiological mechanism.^72^ Tools that assess LV and left atrial (LA) mechanics by measuring strain can overcome these diagnostic challenges.^73,74^ The role of RV mechanics in this context is still under investigation.^75^

### 4.2. Left Ventricle Strain

Several studies have shown that myocardial strain parameters evaluated through speckle tracking, especially LVGLS, have a better correlation with LV relaxation and more accurately predict filling pressures and exercise intolerance than those derived from tissue Doppler.^76-78^ Reduced LVGLS helps detect DD at earlier stages and also predicts cardiovascular events in HFpEF.^17,79-82^ In light of this evidence, reduced LVGLS (<16%) has already been included as a diagnostic criterion in a new algorithm in recent HFpEF guidelines.^83^

### 4.3. Left Atrial Strain

LA strain allows for a more detailed analysis of LA function and its various components (reservoir, conduit, and pump function).^70^ Changes in LA strain express ventriculoatrial coupling and result from chronic exposure to elevated LV pressure, and reductions in LA compliance and relaxation,^41,84,85^ which may precede morphological remodeling.^86-88^ Although reductions in all LA strain components has been described,^86,89,90^ the reservoir LA strain is the most robust parameter, changing linearly with DD progression.^91-93^ Morris et al, among other authors, demonstrated that reduced reservoir LA strain (< 23%) increased DD detection and correlated with filling pressures and clinical outcomes.^93-99^

Due to the growing evidence, LVGLS and LA reservoir strain could be integrated into the current DD algorithm, as proposed in the Central Figure. This strategy may help reclassify indeterminate cases and increase accuracy in identifying earlier stages of DD, especially in individuals with cardiovascular risk factors or unexplained dyspnea.^97^

Standardization of strain methodology has helped minimize variability between manufacturers, which is still a limitation.^7,92,100,101^ New prospective multicenter studies are expected to determine whether modifying these indices with treatment can improve DD and HFpEF prognosis.

### 4.4. Conclusion

LVGLS and LA reservoir strain are subclinical disease markers that can be incorporated into current recommendations to refine the diagnosis, staging, and prognosis of DD. Considering the complex nature of such assessments, using artificial intelligence to validate and implement algorithms would be beneficial.

## 5. Strain in Cardiomyopathies

### 5.1. Introduction

Generally speaking, cardiomyopathies are disorders of the heart muscle. Strictly speaking, they are not associated with certain conditions known to be aggressive to the myocardium, such as coronary artery disease, arterial hypertension, valvular heart disease, and congenital heart disease. They can be divided into the following groups: dilated, hypertrophic, restrictive, arrhythmogenic cardiomyopathy, and “unclassified”.^102^

### 5.2. Dilated Cardiomyopathy

Dilated cardiomyopathy is a disease that, by definition, affects myocardial tissue and leads to a progressive reduction in systolic function and dilation of the LV cavity. Clinically, individuals may present signs and symptoms of HF, requiring treatment, hospitalization, and finally, heart transplantation.^102-107^

Echocardiography, part of the first-line diagnostic arsenal, plays an extremely important role in diagnosis and prognosis. Its main objectives are to determine the volume of the cardiac chambers and assess LV systolic performance. This usually involves estimating the EF, which should be done according to Simpson’s method. Strain assessment is an additional echocardiographic tool that can enrich this process. It allows detection of subtle, subclinical abnormalities in the early stages of disease.

Abduch et al. found excellent correlation between volumetric parameters obtained by 3D echocardiography and strain assessment in patients with dilated cardiomyopathy.^108^ Since the clinical course of dilated cardiomyopathy leads to more pronounced phases of LV systolic impairment, there will be greater reductions in strain and strain rate in the longitudinal, radial, and circumferential components Figure 5.1.^109^ LV torsion also follows this downward trend with disease progression. Additionally, in very advanced stages, the rotations may also be inverted, ie, basal segments rotating counterclockwise and apical segments rotating clockwise.^110-112^

**Figure 5.1:** – A) Example of normal left ventricle global longitudinal strain. Note the negative deflections of the curves, which are relatively homogeneous in the 3 views. In the lower right is the parametric bull’s eye mapping, with all fields in more intense red, indicating good global strain; B) Example of strain in an individual with dilated cardiomyopathy due to Chagas disease. Note the reduced amplitude of the curves, which are also very heterogeneous. The septal and inferior wall strain curves have a positive deflection, ie, indicating distension or dyskinesia, which in the bull’s eye map appears blue (yellow arrows), in addition to pink areas, which indicate low strain values (GLS = -5.6%).

GLS is an independent predictor of all-cause mortality in patients with HFrEF, especially men without AF.^113^ In patients with recovered LVEF, abnormal GLS predicts the likelihood of decreased LVEF during follow-up, whereas normal GLS predicts the likelihood of stable LVEF during recovery.^114^

### 5.3. Arrhythmogenic Cardiomyopathy

Arrhythmogenic cardiomyopathy is histologically characterized by fibrofatty infiltration in the myocardial tissue. Although this infiltration generally occurs in the entrance, exit, and apex of the RV (the “dysplasia triangle”), the LV can also be affected concomitantly or even exclusively.^115-117^

Macroscopically, the ventricular wall tends to thin and forms microaneurysms, progressing to systolic impairment and cavity dilation. The gold standard diagnostic method is cardiac MRI, although echocardiography is the initial examination. RV free wall strain assessment can help determine systolic impairment in this cavity.

In 2007 Prakasa et al. were the first to analyze strain in RV dysplasia/cardiomyopathy, finding a consistent difference in strain values between diseased (10% [SD 6%]) and healthy individuals (28% [SD 11%], P = 0.001).^118^

RV free wall LS is associated with the rate of structural progression in patients with arrhythmogenic cardiomyopathy. It can be a useful marker for determining which patients require closer follow-up and treatment. Patients with RV strain < 20% have a higher risk of structural progression (odds ratio: 18.4; 95% CI, 2.7-125.8; P = 0.003).^119^

Patients with arrhythmogenic cardiomyopathy have reduced right atrial (RA) strain in all diastole phases, even when the RA volume is normal. RA strain, obtained during the reservoir and pump phases, is associated with a greater risk of cardiovascular events.^120^

### 5.4. Hypertrophic Cardiomyopathy

HCM, an autosomal dominant disease, is the most common heart disease of genetic etiology. It is characterized by increased ventricular myocardial thickness of different morphologies (concentric, apical, or septal, hypertrophy of the LV free wall and RV) and is related to increased morbidity and mortality in affected patients.^121-123^

Echocardiography is the most common imaging method for morphological and hemodynamic diagnosis. About 25% of these patients have an LV outflow tract gradient > 30 mm Hg at rest, which can be quantified by continuous Doppler.^124^ The gradient, which may be dynamic in these patients, can be better evaluated echocardiographically either using the Valsalva maneuver or through physical or pharmacological stress with dobutamine.^125^ A high intraventricular gradient in HCM may be a determinant of decreased myocardial deformity and torsion mechanisms, as well as LA strain.^126^

Myocardial strain measured through speckle-tracking helps clarify regional and global cardiac mechanics in HCM and can detect early changes in systolic function, fibrosis, and a greater risk of arrhythmia, even in patients with normal systolic function.^126-130^ Polar mapping helps differentiate phenocopies that lead to increased thickness and reduced longitudinal myocardial strain at the site of hypertrophy^20,131,132^ (Figure 5.2).

**Figure 5.2:** – A) Two-dimensional image demonstrating non-obstructive asymmetric septal hypertrophy, with an interventricular septum measuring 23 mm and a posterior wall measuring 11 mm; B) normal global longitudinal strain (GLS) of the LV (20%), with parametric bull’s eye mapping segments with good myocardial strain are darker red, while pink indicates lower strain values in the septal segments (8%); C) 4-chamber view, with lower myocardial strain in the middle and basal inferoseptal segments; D) note the reduced amplitude of the curves representing septal segments, also shown in pink in the bull’s eye map, representing lower strain values (8%).

Hiemstra et al. found that indexed LA volume and LVGLS were independent prognostic factors for adverse outcomes, such as sudden death and heart transplantation, as well as independent GLS values > 10%.^133^ Although RV global longitudinal strain (RVGLS) may be altered in HCM patients due to structural heart disease, its prognostic significance is unknown.^133,134^

### 5.5. Endomyocardial Fibrosis

Endomyocardial fibrosis is the most common restrictive cardiomyopathy in tropical climates, affecting approximately 10 million people worldwide. It is characterized by subendocardial fibrosis of the apices and inflow tracts of one or both ventricles. Its etiology, which is still unknown, may be related to hypereosinophilia, parasitic infestations, or protein malnutrition, especially in populations of low socioeconomic status.

Echocardiography will show ventricles of normal or reduced size with “mushroom” or “V” ventricular morphology due to fibrosis, which may be associated with apical endocardial thrombosis, hyperkinesis of the basal portion of the LV (Merlon sign), greatly increased atrial volume, often preserved ventricular systolic function, and DD.^135-137^

Although few studies have assessed endomyocardial fibrosis using 3D echocardiography with speckle-tracking, they have found reduced GLS, especially with more severe apical involvement.^137,138^

### 5.6. Noncompacted Myocardium

Noncompacted myocardial tissue is characterized by prominent trabeculae and deep intertrabecular recesses due to incomplete compaction during embryonic life. This can lead to a clinical picture of HF, arrhythmias, and thromboembolic events. Existing in sporadic and familial forms, the latter are related to sarcomere protein mutations. Myocardial strain indices allow a proper regional analysis of ventricular function in patients with noncompacted myocardium and help differentiate it from other forms of HCM.

An Indian study compared the myocardial strain in 12 patients with noncompacted myocardium, 18 patients with HCM, and 18 healthy controls. Both patient groups had reduced LS, although it was lower in the apical region in the noncompacted myocardium group than the HCM group (12.18 [SD, 6.25] vs 18.37 [SD, 3.67]; p < 0.05), suggesting that this region was more involved in myocardial noncompaction. Furthermore, an apical-basal gradient in LS was observed in noncompacted myocardium but not HCM.^139^ DD was found in both patient groups. Another study found greater LS in a LV non-contraction group than a non-specific dilated cardiomyopathies group, reporting that the base-apex mid-wall gradient of strain is a useful index for differentiating these diseases (sensitivity: 88.4 %; specificity: 66.7%).^140^

In normal hearts, the base rotates clockwise during systole, while the apex rotates counterclockwise, with torsion being the apical minus basal rotation. A study found that 50% of patients with noncompacted myocardium have rigid body rotation, with clockwise rotation of the apex and base, although others have found prevalences of 53.3% and 83%.^141,142^ A study of 28 children with noncompacted myocardium found that 39% had rigid body rotation. This group had lower LS, but not LVEF than those without rigid body rotation, which may have prognostic value.^143^ These authors also suggested that rigid body rotation may be related to dysfunction of the compacted subepicardial apical layer and unrelated to trabeculae distribution. Another study of 101 children with noncompacted myocardium found that the adverse outcome group had lower longitudinal, radial, and circumferential strain, which suggests that this disease affects the heart globally rather than just the non-compacted region.^144^

## 6. Strain in Valvular Heart Disease

Due to its combined analysis of changes in anatomy and valve function, transthoracic Doppler echocardiography is the first-line method for diagnosing valvular heart disease and classifying its severity.^145^ This method helps define the best intervention type and time point for treating valvular heart diseases.

Classically, treatment is based on symptoms and complicating factors,^145^with LV dysfunction considered the most important complicating factor.^145^LV function is usually determined through echocardiographic LVEF measurement.^146^

However, there is evidence that LV strain can identify ventricular dysfunction before EF decreases. Perhaps mitral regurgitation can best represent this paradox, since in this condition the high preload and low afterload mean that EF does not adequately represent LV systolic function. For this reason, the definition of LV dysfunction in this condition is quite conservative in clinical guidelines.^145,147-149^ However, some studies have indicated that even with these parameters, clinical outcomes after surgical correction of mitral regurgitation may not be satisfactory, especially regarding EF reduction and the presence of HF.^150-151^ Thus, studies have shown that even in patients with an EF > 60% and an LV end-systolic diameter < 40 mm, reduced GLS (≤ 19%) is associated with a postoperative EF < 50%.^152-154^ GLS < 18.1%, has also been associated with higher mortality and more cardiovascular events in a prospectively followed cohort of mitral regurgitation patients who underwent corrective surgery.^155^

In aortic insufficiency, valvular heart disease severity has been correlated with decreased LV strain.^156^ Furthermore, in patients with asymptomatic severe chronic aortic insufficiency and preserved EF, GLS < 19% was associated with higher mortality over time, which was corrected through valve replacement.^157^ In the same group, postoperatively, GLS < 19% or a > 5% drop in GLS was correlated with higher mortality.^158^

In severe aortic stenosis, EF < 50% and/or symptoms have been the standard factors for indicating treatment.^145,147-149^ However, waiting for the LVEF to drop < 50% before indicating aortic surgery can lead to unsatisfactory clinical outcomes.^159^ Thus, a robust parameter for detecting subclinical myocardial dysfunction, such as GLS, would be a valuable tool in risk stratification (Figure 6.1). While LVEF cannot be used to distinguish between degrees of aortic stenosis, GLS decreases linearly with disease progression,^160^ leading to a higher risk of adverse clinical outcomes, even in asymptomatic patients.^161^

**Figure 6.1:** – Two patients with classic severe high-flow aortic valve stenosis with normal left ventricular ejection fraction (LVEF) but very different global longitudinal strain (GLS) values. (A) Mean aortic transvalve gradient = 61 mm Hg, with normal EF and GLS. (B) Mean aortic transvalve gradient = 88 mm Hg, with normal EF and reduced GLS.

Several studies have examined the prognostic value of GLS for predicting mortality and cardiovascular events in asymptomatic individuals with aortic stenosis and preserved LVEF to determine which ones should receive early valve intervention.^162-164^ In a meta-analysis of these studies, GLS < 14.7% was the cut-off for higher mortality risk (sensitivity 60% and specificity 70%; area under the curve [AUC] = 0.68).^165^ GLS < 14.7% was found in approximately one-third of the patients with moderate-to-severe aortic stenosis and preserved LVEF, resulting in a 2.6 times higher mortality risk. Of note, the relationship between GLS and mortality was significant in patients with LVEF 50%-59% as well as in those with LVEF ≥ 60%. In contrast, GLS > 18% was associated with an excellent clinical outcome (97% [SD, 1%]; 2-year survival). Therefore, together with other clinical and echocardiographic data, reduced GLS, despite preserved LVEF, must be considered a strong prognostic predictor in clinical decision making about asymptomatic severe aortic stenosis.

## 7. Strain in Ischemic Heart Disease

### 7.1. Introduction

Echocardiography is an excellent tool in emergency departments for diagnosing acute coronary syndrome and its complications, providing information about the patient’s short- and long-term prognosis. Its role is well defined in risk stratification for stable coronary artery disease and in myocardial viability assessment.

Two-dimensional echocardiography with strain assessment through 2D speckle tracking can add information without greatly extending the examination time. It accurately assesses subendocardial ischemia through LS in acute and chronic events.

This chapter will review all indications for longitudinal, circumferential, and radial strain in ischemic heart diseases, as well as other data involved in calculating strain, such as mechanical dispersion. Table 7.1 shows the main indications for strain assessment in ischemic heart disease.

**Table 7.1:** – Main applications of strain assessment in ischemic heart disease

Strain indications in ischemic heart disease
Assessing segmental and global change
Differentiating subendocardial and transmural infarcts (longitudinal, radial, and circumferential change)
Determining the culprit artery according to “bulls eye” mapping
Assessing improvement in global and segmental strain after myocardial revascularization
Improving detection of coronary artery disease
Predicting outcomes and remodeling after acute mysocardial infarction
Predicting outcomes, such as hospitalization for heart failure and all-cause mortality
Identifying patients at risk of arrhythmia

### 7.2. Strain in Acute Coronary Syndrome

Two-dimensional strain assessment has good sensitivity for detecting myocardial ischemia. It is considered more reproducible than LVEF and its accuracy has been confirmed in cardiac MRI.^52,166^ Subendocardial fiber is more sensitive to the initial stages of ischemia, with the longitudinal component predominating in this type.^167^ GLS is reduced in acute myocardial infarction (AMI) and correlates with the extent of the infarction, EF, adverse events, and response to reperfusion strategies.^168-172^

Patients with small infarcts have reduced global and radial strain, while circumferential and twist strain remain preserved. However, circumferential strain is compromised in transmural infarction.^173^

Identifying the extent of transmural infarction is important, since it is associated with poor prognosis and adverse events. Subendocardial and non-transmural infarcts are associated with recovery after revascularization (Figure 7.1).^174^

**Figure 7.1:** – Two-dimensional bull’s eye mapping showing strain in the apical region of the left ventricle; the reduced segmental values are compatible with injury in the anterior descending artery.

A strain value of 15% correlates with segmental changes (76% sensitivity and 95% specificity).^168^At a cutoff of 16.5%, radial strain differentiates transmural from non-transmural infarctions (70% sensitivity and 71.2% specificity), while circumferential strain < 11% differentiates transmural from non-transmural infarctions (70% sensitivity and 71.2% specificity),^175^ and 4.5% regional LS distinguishes transmural from non-transmural infarctions (81.2% sensitivity and 81.6% specificity).^176,177^

Another important diagnostic benefit of GLS is that it can help determine the culprit artery in non-ST-elevation acute coronary syndromes. In a cohort of 58 patients, 33 with significant coronary artery disease (lesion > 50% determined through coronary angiography) who underwent strain analysis prior to the procedure, a cut-off of 19.7% could detect acute coronary disease (sensitivity 81%, specificity 88%, AUC = 0.92). A cut-off of 21% could exclude significant coronary stenosis in 100% of the patients. Regional LS was calculated as the mean systolic peak strain of each segment in the region of the studied vessel. If a cut-off of 21% were applied to the sample, 16 patients would have been spared from coronary angiography.^178,179^

Strain analysis can help detect acute coronary occlusion in patients without ST elevation who may benefit from early reperfusion therapy. One study evaluated 150 patients who underwent an echocardiographic examination prior to referral for coronary angiography, finding that 33 had an acute coronary occlusion. Although strain < 14% identified acute coronary occlusion with 85% sensitivity and 70% specificity, more robust studies are needed to validate the technique.^180^

Strain analysis has emerged as a new technique for detecting subclinical segmental and global changes. Alongside enzymatic tests, electrocardiography, and risk scores, it enhances the prognostic evaluation of patients with acute coronary disease. This quick examination can be performed at bedside prior to coronary angiography, especially by trained echocardiographers. The above-mentioned studies on non-ST-elevation acute coronary syndromes indicate the technique for assessing segmental alterations and global ventricular function, differentiating small and transmural infarctions, determining the likely culprit artery, and for evaluating percutaneous revascularization outcomes. It can also be used to assess myocardial viability after an AMI.^181,182^

### 7.3. Strain in Chronic Coronary Syndromes

The subendocardial region, in which the fibers are oriented longitudinally, is the most susceptible area to ischemia. Thus, assessing LS with 2D speckle-tracking could be a better method of detecting ischemia in this region than conventional echocardiography.^183^

Interaction between normal and abnormal myocardium generates typical regional myocardial strain patterns, indicating that myocardial contraction and myocardial strain are not interchangeable parameters.^16,184^

Since it assesses longitudinal LV function, GLS may be much more sensitive than LVEF for early detection of myocardial ischemia, although its specificity is not better than changes in wall mobility.^185,186^

The variability of regional strain measurements in speckle tracking is relatively high, which makes these assessments less suitable for routine use. However, GLS measurements have proven reproducible and robust, probably due to being largely automated.^187^ The other change is regional heterogeneity of myocardial activation, which affects the temporal sequence of myocardial contraction and relaxation.

In ischemia, not only is the amplitude of contraction reduced, but the beginning and duration of fiber contraction change, which leads to characteristic shortening or thickening of the myocardium after aortic valve closure.^187^ This “post-systolic shortening” is characteristic of ischemia, although it can also occur in any type of regional dysfunction (scarring, dyssynchrony, etc.).^187,188^ Post-systolic shortening can be understood as a signal to delay relaxation so that the ischemic region can shorten while the LV pressure reduces and the surrounding tissue relaxes.^16^ In healthy hearts, less post-systolic shortening with normal systolic function is a frequent finding (30%-40% of myocardial segments) and can be found mainly at the apex and base of the inferior, septal, and anteroseptal walls.^16,189^ In the context of ischemic cardiomyopathy, temporal evaluation of the GLS curve pattern is important, since ischemic segments can often have preserved peak systolic values but are temporally delayed in relation to non-ischemic segments.

It should be pointed out that regional LS measurements do not necessarily align visually with contraction changes, which involve radial thickening and endocardial movement into the cavity.^16^

GLS can help detect coronary artery disease in patients with stable angina (stenosis ≥ 70%), having reduced values in the presence of coronary artery disease (17.1 [SD 2.5%] vs 18.8 [SD 2.6%]; p < 0.001), especially when associated with exercise testing. It can also determine which artery has likely been affected.^183^ LS and (especially) strain rate can detect segmental changes in the late post-myocardial infarction period with higher sensitivity and accuracy.^189^

In stratifying post-AMI patients, GLS < 15% prior to hospital discharge was an independent predictor of LV dilation in 3-6 months of follow-up, in addition to indicating the size of the infarct area.^190^In the same context, GLS < 14% was an independent predictor of cardiovascular death and hospitalization due to HF.^191^ In stable chronic patients, GLS < 11.5% was a predictor of all-cause mortality and the combined outcome (all-cause mortality and hospitalization for HF).^192^

Heterogenous regional myocardial contraction can also be assessed by mechanical dispersion, which is the standard deviation of the time to maximum myocardial shortening across all LV segments. This index has a predictive value for ventricular tachyarrhythmia in post-infarction patients. Mechanical dispersion was higher in patients with recurrent arrhythmias after AMI than in those without them (85 [SD, 29] ms vs 56 [SD, 13] ms, p < 0.001).^193^

### 7.4. Right Ventricular Strain in Ischemic Heart Disease

RV function is compromised in approximately one-third of inferior wall infarctions, and its involvement has been described as an important predictor of hospital mortality and major complications. Assessing RV function is challenging due to its structural complexity. RV free wall strain has been shown to predict proximal right coronary artery occlusion in patients with inferior wall AMIs (RV free wall strain < 14.5%, AUC = 0.81; p < 0.001) .^194^

In the stable phase of chronic ischemic disease, RV free wall strain is altered in patients with right coronary stenosis (lesions > 50%) and can be used to detect subclinical dysfunction.^19 5^

## 8. Strain Assessment in Systemic Diseases (Amyloidosis and Fabry Disease)

### 8.1. Strain Assessment in Cardiac Amyloidosis

Amyloidosis is a systemic disease caused by extracellular deposition of insoluble amyloid fibrils in tissue. Cardiac involvement is an important prognostic factor and has a great impact on quality of life, occurring more commonly in light chain amyloidosis and transthyretin amyloidosis.^196^

Echocardiography is a first-line method for diagnosis and prognostic evaluation of cardiac amyloidosis (CA) and other infiltrative heart diseases. Most classic echocardiography findings and more specific signs of CA only occur at very advanced stages.^30^ Clinical conditions such as HFpEF and ventricular hypertrophy may be warning signs for CA.^197^

#### 8.1.1. Myocardial Strain Assessment in the Diagnosis of Cardiac Amyloidosis

The consistent LVGLS changes in patients with CA are directly related to the degree of amyloid infiltration, which is quantified in MRI through the degree of delayed gadolinium enhancement and extracellular volume calculated in T1 imaging.^30^ A relative apical sparing pattern of LS has been described in the literature, characterized by a basal-apical gradient (“cherry-on-top” pattern) (Figure 8.1).

**Figure 8.1:** – Longitudinal 2D strain of the left ventricle in a patient with cardiac transthyretin amyloidosis, showing relative apical preservation (apical sparing pattern), with lower strain in the middle and basal segments and higher strain in the apical segments.

Phelan et al., originally calculated relative apical sparing with the following equation: mean apical LS/(mean LS of the middle segments + mean LS of the basal segments). Values > 1.0 had good accuracy in diagnosing CA and differentiating it from ventricular hypertrophy due to aortic stenosis and HCM (AUC: 0.94).^20^

This regional LS pattern, with its basal-apical gradient, is indistinct in immunoglobulin light chain amyloidosis and transthyretin amyloidosis. It should be pointed out that the classic apical sparing pattern, although generally characteristic of CA, may be absent, as reported by Ternacle et al., who found that 52% of CA patients had “non-diagnostic” relative apical sparing (< 1.0).^198^ In some cases, this could be explained by a low degree of amyloid infiltration in the myocardium in very early stages of the disease. LS > 2.1 in the basal and apical regions of the septum associated with a mitral inflow deceleration time < 200 ms also accurately differentiated CA from other diseases involving LV hypertrophy and a parietal phenotype, such as Fabry disease, Friedreich’s ataxia, and LV hypertrophy related to systemic arterial hypertension.^199^

An LVEF/GLS ratio > 4.1 accurately differentiated CA from HCM, performing better than relative apical sparing or LS in the basal and apical regions of the septum, regardless of CA type.^200^ RV myocardial strain is generally reduced in CA patients, and it may help differentiate CA from other causes of parietal hypertrophy (Figure 8.2). A relative apical sparing pattern has also been found, similar to what has described in the LV.^201^ Bellavia et al. demonstrated that RV changes can occur early in patients with immunoglobulin light chain amyloidosis, even in cases where LV parietal thickness is still normal.^202^

**Figure 8.2:** – Longitudinal 2D strain assessment in the right ventricle of a patient with cardiac transthyretin amyloidosis, showing a lower absolute global value (RVGLS = 9.9%) and an absolute reduction in mean free wall strain (FWS) (11.4%), with lower values in middle and basal segments and higher values in apical segments. TAPSE: tricuspid annular plane systolic excursion.

In CA, like other infiltrative cardiomyopathies, other myocardial strain components may be significantly compromised, such as circumferential strain,^203^ radial strain,^204^ and twist (Figure 8.3). In early-stage systemic amyloidosis with no evidence of CA, twist and untwist may be increased in compensation,^205^ with these parameters progressively deteriorating during the clinical course of the disease.^206^ In advanced cases, the cardiac base and apex may rotate in the same direction, creating a pattern called “rigid body rotation”, in which the important contribution of cardiac torsion to ventricular mechanics is completely lost.

**Figure 8.3:** – Transthyretin amyloidosis. Significantly lower absolute left ventricular (LV) radial strain values in all basal segments in 2D assessment (A). There is also a change in circumferential strain, resulting in significantly reduced “twist” (4º) and LV torsion (B).

LA strain often undergoes significant change in CA, due partially to LV DD but also to direct amyloid fibril infiltration in the atrial wall (Figure 8.4). In a recent study by Aimo et al., only peak atrial LS (in addition to classic echocardiographic variables and cardiac biomarkers) was independently associated with CA diagnosis.^207^ It was also found that advanced atrial infiltration could cause severe dysfunction and loss of mechanical efficiency, leading to atrial electromechanical dissociation.^208^ In a large cohort, Bandera et al. found atrial electromechanical dissociation (determined by LS analysis) in 22.1% of the patients in sinus rhythm, which was a determining factor for poor prognosis compared to patients in sinus rhythm with preserved atrial mechanical function.^209^ In a Mayo Clinic series of 156 CA patients, intracardiac thrombi were detected by transesophageal echocardiography in 27%,^210^ which has been reproduced in other studies, with thrombi occurring even in patients in sinus rhythm (Figure 8.5).^211,212^

**Figure 8.4:** – Immunoglobulin light chain amyloidosis. Two-dimensional left atrial (LA) strain, with 4-chamber and 2-chamber apical windows, showing an important reduction in LA strain (reservoir strain = 4%).

**Figure 8.5:** – Transthyretin amyloidosis. The 2D apical window shows: (A) a large mobile mass inside the right atrium in a patient with sinus rhythm; (B) a 3D-rendered transesophageal echocardiography image showing a large mass (thrombus) adhered to the right atrial appendage. IAS: interatrial septum; SVC: superior vena cava; TV: tricuspid valve.

Three-dimensional strain assessment can help demonstrate changes in all components of myocardial strain in patients with CA. Vitarelli et al. found that LV peak basal rotation and RV and LV basal LS could accurately distinguish CA from other ventricular hypertrophies.^213^ In 3D echocardiography LS, Baccouche et al.^214^ found the same apical sparing pattern, with a characteristic basal-apical gradient.

Myocardial work (MW) has also been assessed in patients with CA. Clemmensen et al. found that patients with CA had a lower left ventricular myocardial work (LVMW) index than the control group, with more pronounced changes in basal segments and when undergoing stress echocardiography. The LVMW index from rest to peak exercise increased 1974 mm Hg% in controls (95% CI, 1699-2250 mm Hg%; P < 0.0001) but only 496 mm Hg% in CA patients (95% CI, 156-835 mm Hg%; P < 0. 01).^215^

The use of strain to assess clinical course and monitor response to specific CA treatments is very promising. Giblin et al. retrospectively evaluated 45 patients with cardiac transthyretin amyloidosis at 1 year of follow-up, comparing LS and MW values between groups of patients treated or not with Tafamidis.^216^ In untreated patients, they found greater deterioration in GLS (p = 0.02), LVMW index, and MW efficiency (p = 0.04), with no significant differences between groups in circumferential strain, radial strain, or twist.

Myocardial deformation parameters have also been extensively studied as prognostic indices in CA due to their ability to provide quantitative data, as well as to their high sensitivity and reproducibility. At a mean follow-up of 11 months Ternacle et al. found that mean apical LS (cut-off: -14.5%), elevated N-terminal pro–B-type natriuretic peptide, and New York Heart Association functional class III or IV were independent predictors of major cardiovascular events.^198^ In another study, relative apical sparing index was independently associated with a composite outcome of death or heart transplantation within 5 years (hazard ratio [HR] 2.45; p = 0.003), maintaining its predictive value even in the multivariate analysis (p = 0.018).^217^

A large study by Buss et al. of 206 patients with systemic light chain amyloidosis found that Doppler-based LS and GLS were strongly associated with N-terminal pro–B-type natriuretic peptide levels and survival (best cut-off: -11.78%); in the multivariate analysis, only DD and GLS remained as independent predictors of survival.^218^

A recent study by Liu et al. included 40 patients with multiple myeloma with preserved EF before beginning bortezomib treatment, measuring baseline GLS and MW parameters. Global MW efficiency was significantly associated with adverse cardiac events after 6 months of chemotherapy (AUC = 0.896; 95% CI: 0.758-0.970; p < 0.05).^219^ RVLS has also been associated with CA prognosis. In a study of 136 patients with CA, Huntjens et al. found that strain values in all cavities were significantly associated with survival in median follow-up of 5 years.^220^ Peak LALS and mean RV free wall strain continued to be independently associated with prognosis in the multivariate analysis. As an independent variable, peak LALS had the most robust association with survival (p < 0.001), while the greatest prognostic value was obtained by combining LALS, GLS, and mean RV free wall strain (p < 0.001).

## 8.2. Fabry Disease

Fabry disease (or Anderson-Fabry disease) is the most common glycogen storage disease, affecting 1 in 50,000 individuals.^221^ Being an X-linked recessive disease, it is more common in men; women are carriers without alpha-galactose activity. The condition leads to a progressive accumulation of globotriaosylceramide in the kidneys, heart, and nerves. Clinically, patients present with skin changes (angiokeratomas), peripheral neuropathy, renal failure, and HF due to restrictive cardiomyopathy with increased myocardial thickness. Although these clinical manifestations can occur in childhood, they are more common after the third decade of life.^222^

Morphological analysis shows characteristically increased LV thickness, which may progress to a reduced compliance and HFpEF due to restrictive cardiomyopathy. Other interesting findings that may be considered red flags are papillary muscle hypertrophy, double contour sign, and dynamic obstruction of the LV outflow tract.^223^ This similar phenotype to HCM is described in 6% of men^224^ and 12% of women diagnosed in later age.^225^ On the other hand, bull’s eye parametric analysis of LVLS plays an important role in differentiating cardiomyopathies that involve increased myocardial thickness, especially in asymmetric LV hypertrophy with an etiological possibility of HCM, amyloidosis (mainly transthyretin), Fabry disease, or hypertensive heart disease in older patients.

HCM typically involves lower segmental values and greater thickness, while amyloid cardiomyopathy produces an apical sparing pattern and has a greater effect on the middle and basal regions of the LV. On the other hand, hypertensive heart disease can present with slightly reduced GLS. Curiously, Fabry disease involves a unique pattern in which, despite an asymmetrical increase in ventricular thickness, the basal portion of the inferolateral wall is most affected by LS (Figure 8.6); a progressive decreasing pattern occurs in untreated disease. There is a good correlation between LVLS assessment and delayed-enhanced cardiac MRI in different stages of the disease.^222^ In addition, a study compared Fabry disease patients without myocardial alterations to a healthy control group, finding significantly lower myocardial values for LV, RV, LA in the Fabry group than the control group (18.1 [SD, 4.0], 21.4 [SD, 4.9], and 29.7 [SD, 9.9] vs 21.6 [SD, 2.2], 25.2 [SD, 4.0], and 44.8 [SD, 11.1%], respectively, P < 0.001). Interestingly, in addition to these differences, strain changes correlated well with symptom severity.^226^

**Figure 8.6:** – Global longitudinal strain in a patient with Fabry disease: bull’s eye map shows the lowest values in the in the basal portions of the left ventricle inferolateral wall. AVC: aortic valve closure.

Treatment is currently available for Fabry disease, and morphological changes in the heart, such as reduced thickness, can appear after 1 year of treatment.^227,228^ Thus, improved LVLS is expected to occur earlier, even before ventricular mass reduction. However, therapeutic response and GLS patterns throughout treatment should still be investigated.

Myocardial strain assessment is an important tool for diagnosing ventricular hypertrophies of unknown etiology, mainly within a coherent clinical context and a good echocardiographic window, including follow-up of family members without access to genetic assessment or therapeutic response (Figure 8.6).,

## 9. Strain in Hypertension

### 9.1. Introduction

This section discusses the main advantages and disadvantages of strain assessment in hypertension, with and without criteria for hypertensive cardiomyopathy (ie, LV hypertrophy), and its current clinical value.

### 9.2. Hypertension without Left Ventricular Hypertrophy Criteria

During its clinical course, hypertension causes changes in myocardial contractility, which appear as reduced LS in response to afterload and high wall systolic stress. These changes have proven prognostic significance. Reduced GLS reflects subclinical myocardial dysfunction even before the onset of LV hypertrophy or EF reduction (detected through traditional measurement methods); strain is the only measurement to change in stage I hypertension.^229-239^GLS reduction initially occurs in the basal region of the interventricular septum, extending to the basal and middle regions of other walls, which is likely because of overload in the interventricular septum due to systolic wall stress in the early stages of hypertension (Figure 9.1).^240,241^ The longitudinal fibers of the subendocardial layer are affected early in this phase, along with the mesocardium, but not the epicardium, as suggested in some studies.^242^

**Figure 9.1:** – Examples of global longitudinal strain polar mapping with peak systolic strain curves obtained in 4, 2 and 3 chambers. (A) healthy non-hypertensive patient with preserved GLS; (B) hypertensive patient without LV hypertrophy whose GLS is at the lower limit of normality (note altered myocardial strain in the basal septum); (C) hypertensive patient with LV hypertrophy and reduced GLS (note greater change in myocardial strain in basal and middle segments than apical segments).

Another study found that LS change in the epicardial layer was the only variable that could predict cardiovascular events, indicating that it may correspond to the most severe and chronic type of damage.^243^ However, the majority of currently available devices cannot analyze myocardial layers separately. On the other hand, radial and circumferential strain, which are based on overall myocardial thickness, tend to remain unchanged or even increase in a probable attempt to mechanically compensate for lower GLS,^60,236^ with a change in circumferential component indicating more severe myocardial dysfunction.^244^

The main explanations for reduced GLS are an increase in the synthesis of collagen, culminating in fibrosis, which is a strong marker of myocardial dysfunction. Reduced GLS correlates not only with plasma markers of fibrosis, such as elevated tissue metalloproteinase inhibitor, but with fibrosis detected through delayed gadolinium-enhanced MRI in hypertensive patients.^231,234,238,239^ Decreased GLS has also been observed in patients with masked and white-coat hypertension,^245,246^ being correlated with conventional echocardiographic markers of DD,^240^ as well as greater long-term deterioration in individuals who discontinue antihypertensive treatment.^247^

### 9.3. Hypertension with Left Ventricular Hypertrophy Criteria

The myocardial consequences of chronic hypertension include myocyte hypertrophy, myocardial fibrosis, and medial thickening of the intramyocardial coronary arteries.^248^ Consequently, hypertension and changes in myocardial remodeling are risk factors for major cardiac events, such as HF and premature death. Thus, the purpose of strain analysis in these cases is to detect subtle changes in systolic function, even before conventionally determined EF is compromised, thus facilitating the selection of HFpEF cases for treatment.

LV remodeling may affect various types of strain. In concentric hypertrophy, for example, GLS values can progressively decrease according to geometric type, from concentric remodeling to eccentric hypertrophy with LV dilation.^249-251^ Although most studies find preserved global circumferential strain and global radial strain values,^252^ some series have found them to be reduced^249^ and that they tend to remain normal in the epicardial layers in individuals with hypertension and LV hypertrophy.^250^ Torsion and twisting behavior can also vary, with normal or reduced values according to the ventricular geometry.^249,253^ In 3D analysis of hypertensive patients, GLS tends to deteriorate according to the degree of hypertrophy and LV cavity diameter.^62,250^

In addition to the correlation between reduced GLS and different patterns of LV hypertrophy, strain assessment can clarify the cause of hypertrophy, since it is more often reduced in HCM than LV hypertrophy due to hypertension.^254^

### 9.4. Clinical Treatment

GLS decreases concomitantly with functional class^241^ and increases with long-term treatment, as found in 3-year follow-up after antihypertensive treatment^255^ and described in case reports of emergency antihypertensive treatment.^256^ Reduced GLS is also correlated with abnormal blood pressure in patients undergoing treatment, even after adjusting for other clinical variables, such as age, diabetes, and LV mass index.^257^

### 9.5. Conclusion

There is enough evidence to recommend strain assessment for patients with hypertension, regardless of LV hypertrophy, both to identify subclinical structural alterations and to identify the best treatment for HFpEF conditions. On the other hand, more robust studies are needed to guide systematic assessment in this population

## 10. Strain in Athletes

Regular intense physical activity results in a series of profound adaptive electrical, structural, and functional changes, usually referred to as athlete’s heart.^258^ Analysis of this condition is important for a better understanding of the mechanisms of cardiac adaptation and how to improve training to optimize performance. Analysis can also differentiate pathologies with similar morphological characteristics to those induced by training.

High-performance athletes with large LV volumes are considered to be on the healthy physiological spectrum of athlete’s heart. Some studies have reported slightly lower GLS in resting athletes than sedentary people, while others report the opposite.^259,260^ However, most studies have found no significant difference between these groups.^261^ This variation may be related to the effects of different factors such as preload and afterload, myocardial mass, and sinus bradycardia. Thus, reduced GLS values in athletes with normal or supranormal LV diastolic function may help distinguish between cardiac pathologies and secondary adaptations to exercise. Absolute values ≥ 18% are still considered within normal limits. Much greater reductions in these indexes are found in patients with HCM and systemic arterial hypertension.^262^However, global circumferential strain and radial strain were found to differ significantly between athletes and controls.^261^ Bull’s-eye mapping can help differentiate athlete’s heart from other diseases involving hypertrophy.^263^

When athletes are categorized according to exercise type and intensity (static vs dynamic), differences arise predominantly in mechanical aspects of the LV. A recent study showed that cardiac torsion was greater in low dynamic/high static (weightlifting, martial arts) athletes and in low static/high dynamic (marathon, soccer) athletes than controls. In contrast, torsion was lower in high dynamic/moderate static (swimming, water polo) athletes than controls, which could be explained by changes in apical, but not basal, rotation. Peak untwisting was higher in low dynamic/high static athletes and lower in high dynamic/high static athletes.^261^ Studies using speckle tacking to quantify myocardial strain have shown that competitive endurance athletes have normal or increased strain values.^264-268^

In the RV, tissue Doppler and speckle tracking myocardial strain indices may be slightly lower in the basal and middle segments of the RV free wall (notably in endurance athletes) than controls.^269^ It is still unclear whether this reduction is an adaptive response to exercise or a subclinical change due to myocardial injury.^270^ Some authors assume that it can be explained by curvature changes between the apex and base of the RV, resulting in a strain difference between segments.

Speckle tracking studies of atrial function in athletes are still in their infancy and show conflicting results. One study found that LA contraction assessed by GLS significantly decreased after training.^271^ Another study found no differences in atrial strain between athletes and sedentary controls.^272^

Strain measurement is also important in diastolic function assessment. Dynamic exercise leads to more effective ventricular relaxation and biventricular dilation, while static exercise may be related to increased myocardial thickness and LV concentric hypertrophy, which could lead to some degree of diastolic impairment.^273^ In addition, illicit performance-enhancing drugs can lead to deterioration of ventricular, systolic, and/or diastolic function, and speckle tracking echocardiography can detect these alterations early.^274^

Thus, when evaluating ventricular function in professional and/or amateur athletes, all available tools in the echocardiography arsenal are to be used. Strain analysis can detect incipient changes in systolic function, long before contractility changes or EF reductions can be detected in 2D assessment.

When evaluating athletes, speckle tracking echocardiography has shown great promise as a complement to routine 2D echocardiography. Unlike sedentary individuals, absolute GLS values > 16% in this population can be considered normal; lower values should raise suspicion of pathology, especially when combined with signs such as significant ventricular hypertrophy or dilation.^275^

## 11. Strain in Stress Echocardiography

Table 11.1 shows the main applications of strain in stress echocardiography.

**Table 11.1:** – Strain applications in stress echocardiography

Clinical scenario	Concept
Ischemia detection^189,276-280^	•Regional LS detects endocardial ischemia. •CS can help differentiate transmural and non-transmural infarctions. •Increased time to peak LS can help detect ischemia. •Post-systolic shortening (sensitive but not specific for detecting ischemia). •Systolic RLS (lower dependence on load and heart rate than strain). •Diastolic strain parameters.
Feasibility assessment^281-285^	•LS and RLS increase the accuracy of viability detection in MI patients treated with TCA and low doses of dobutamine. •LS is the best predictor of functional improvement after CABG. •Lack of response to dobutamine accurately predicts lack of improvement after CABG. •Differentiation between stunned and hibernating myocardium (lower LS and RLS and post-systolic shortening).
Aortic stenosis^286,287^	•GLS in the evaluation of low-flow/low-gradient aortic stenosis. •Patients with GLS > 10% after dobutamine use have longer survival. •Stress GLS is more accurate than resting GLS.
Hypertensive cardiomyopathy^288^	•LS and RS deficits are more evident during stress than rest in the initial phases, allowing prevention measures.
Diabetic cardiomyopathy^289,290^	•Lower RLS in middle and basal segments during DSE. •Early decreased myocardial velocities in asymptomatic patients with insulin resistance during DSE.
Cardiomyopathies^132,291-293^	•Lower systolic functional reserve detected in LS and RS after stress. •No improvement in diastolic function parameters. •Increased time-to-peak in HCM with important dyssynchrony. •Low RVLS values in arrhythmogenic dysplasia do not significantly improve with stress.
Athlete’s heart^294,295^	•Slightly lower LS values with preserved or supranormal myocardial reserve. •Stress GLS helps differentiate athlete’s heart from cardiomyopathy.

*CABG: coronary artery bypass grafting; CS: circumferential strain; DSE: dobutamine stress echocardiography; GLS: global longitudinal strain; HCM: hypertrophic cardiomyopathy; LS: longitudinal strain; MI: myocardial infarction; RLS: regional longitudinal strain; RS: radial strain; RV: right ventricle; TCA: transluminal coronary angioplasty.*

A more complete review article on stress echocardiography will soon be published in this journal.

## 12. Strain in Congenital Heart Disease

Some studies have demonstrated the high prognostic value of speckle tracking strain assessment, reinforcing its usefulness for both congenital and acquired pathologies.^9^However, myocardial strain is subject to physiological variations due to age, sex, heart rate, preload, blood pressure, and body surface area, as well as the analysis software.^296^ Continuous effort has been made to determine normal strain values for universal reference in pediatrics so that myocardial strain assessment of can be included in the clinical routine.^297-299^

Tables 12.1 [Table t9001]to 12.3 present the myocardial strain values found in the literature for normal children and those with congenital heart diseases, while Table 12.4 shows the recommended cut-off values. A more complete review article on the subject is forthcoming in this journal.

**Table 12.1:** – Normal left ventricular strain values according to age group298

Age in years	Peak systolic GLS	Peak strain in apical 4-chamber view	Peak circumferential systolic global strain	Peak circumferential systolic strain at the papillary muscle level	Peak radial global systolic strain	Peak radial systolic strain at the papillary muscle level
0-1	18.7% (20.8, 16.7)	19.4% (22.2, 16.6)	_____	18.2% (22.6, 13.7)	_____	44.4% (36.6, 52.1)
2-9	21.7% (23.0, 20.5)	21.0% (21.8, 20.2)	24.5% (27.2, 21.7)	20.3% (21.4, 19.1)	48.0% (33.3, 62.8)	50.8% (47.4, 54.1)
10-13	20% (20.8, 19.1)	20.5% (21.7, 19.2)	21.9% (26.5, 17.4)	21.5% (23.1, 19.8)	43.7% (33.0, 54.5)	52.1% (48.5, 55.8)
14-21	19.9 (20.6, 19.2)	19.9% (21.2, 18.6)	16.4% (23.3, 9.6)	16.4% (23.3, 9.6)	44.0% (41.6, 46.4)	46.4% (39.7, 53.1)
Overall	20.2% (20.8, 19.6)	20.4% (21.1, 19.8)	22.3% (19.9, 24.6)	20.4% (21.1, 19.8)	45.2% (38.8, 51.7)	49.4% (47.2, 51.6)

*GLS: global longitudinal strain. Values expressed as mean and 95% CI.*

**Table 12.2 t9001:** – Normal right ventricular strain values according to age group300

	31 days- 24 months	2-5 years	5-11 years	11-18 years
GLS RV %	25.4 (3.9)	25.9 (4.0)	25.8 (4.7)	25.4 (4.1)

*GLS: global longitudinal strain; RV: right ventricle. Values expressed as mean (SD)*

**Table 12.3:** – Valores normais de strain atrial direito e esquerdo, segundo as faixas etária**301**

Measure	31 days- 24 months	2-5 years	5-11 years	11-18 years
**LA strain (R)%**	52.8 (10.1)	55.7 (10.7)	58.1 (10)	57.6 (10.5)
**LA strain (C)%**	14.2 (6.6)	12.7 (6.1)	14.0 (6.7)	15.1 (7.0)
**RA strain (R)%**	47.1 (9.6)	49.6 (10.2)	51.6 (10.7)	52.0 (10.6)
**RA strain (C)%**	11.5 (6.0)	11.9 (5.9)	11.8 (6.3)	12.8 (5.8)

*C: atrial contraction phase; LA: left atrium; R: reservoir phase; RA atrium. Values expressed as mean (SD)*

**Table 12.4:** – A selection of studies evaluating the use of strain in idiopathic pulmonary hypertension and congenital heart disease

	Authors	Parameter	Cut-off	Findings	Sn/Sp
Idiopathic pulmonary hypertension	Muntean et al.^302^	mid-segment FW RV strain	18.5%	Predictor of clinical worsening	Sn: 91.7%, Sp: 30.8% AUC=0.88, SD 0.06
Ebstein's anomaly	Kühn et al.^303^	RVGLS (6 segments)	20.15%	Diagnosis of RV dysfunction (EF < 50% in MRI)	Sn: 77% Sp: 46%
Systemic RV (PO TGA Senning/Mustard)	Lipczyńska et al.^304^	RVGLS (6 segments)	14.2%	Diagnosis of RV Dysfunction (EF< 45% in MRI)	Sn: 83%, Sp: 90% AUC= 0.882
Systemic RV (CTGA)	Kowalik et al.^305^	RVGLS (6 segments)		Diagnosis of RV dysfunction (EF < 45% in MRI)	Sn: 77.3% Sp: 72.7%
PO AOLCA	Castaldi et al.^306^	LV peak segmental strain	14.8%	Predictor of fibrosis in MRI	Sn: 92.5% Sp: 93.7%
Univentricular physiology (PO Fontan)	Park et al.^307^	Circumferential strain rate	-1.0s^-1^	Predictor of hospital stay >14 days after TCC anastomosis	Sn: 72% Sp: 60%

*AOLCA: anomalous origin of the left coronary artery; AUC: area under the curve; TCC: total cavopulmonary connection; CTGA: corrected transposition of the great arteries; EF: ejection fraction; FW: free wall; GLS: global longitudinal strain; MRI: magnetic resonance; PO: postoperative; RV: right ventricle; Sn: sensitivity; Sp: specificity.*

## 13. Right Ventricle Strain

### 13.1. Introduction

The RV plays an important role in the pathophysiology of cardiopulmonary disease. A large amount of evidence has shown that RV dysfunction is an important independent marker of morbidity and mortality in clinical conditions such as HF, valvular heart disease, pulmonary hypertension, pulmonary embolism, and ischemic heart disease, as well as in congenital heart disease in adults.^308-313^

Cardiac MRI is the gold standard non-invasive test for RV volume, EF, and structural assessment. However, its main limitations are its high cost, longer image acquisition time, and poor availability in most centers.^314^ Two-dimensional echocardiography is the most common initial test for structural and functional evaluation of the RV due to its wide availability, low cost, non-invasiveness, and shorter image acquisition time. However, 2D RV assessment is challenging due to the complex structure of the cavity, its unfavorable position in the thoracic wall, and intense myocardial trabeculation, which prevents better visualization of the endocardium due to its thinner walls and high dependence on loading conditions in the most common systolic function indices.^315^

Several echocardiographic parameters are used to indicate RV systolic function in clinical practice. Recently, 2D speckle-tracking was introduced into clinical practice as an objective indicator of regional and global myocardial contractility, initially for LV and more recently for RV evaluation. Research has highlighted the advantages of this new methodology over conventional echocardiographic parameters.^316^

### 13.2. Anatomical and Functional Characteristics of the Right Ventricle

Table 13.1 shows the main characteristics that differentiate the ventricles.^317-319^ LV and RV function are closely related (a phenomenon called systolic ventricular interaction) since they share obliquely arranged muscle fibers in the interventricular septum. These have a mechanical advantage over the transverse fibers of the RV free wall.^36^ The continuity of these muscle fibers allows the RV free wall to be pulled during LV contraction, with an estimated 20%-40% of RV stroke volume and systolic pressure due to LV contraction.^318,319^

**Table 13.1:** – Anatomical and functional characteristics of the ventricles

	Right ventricle	Left ventricle
Structure	Inlet, trabecular region, infundibulum.	No infundibulum and limited trabeculation.
Form	Multiple muscle bands Triangular in the coronal plane Crescent in the transverse plane	Elliptical
Predominant myocardial fiber orientation	Subepicardium: circumferential Subendocardium: longitudinal	Subepicardium: oblique Mesocardium: circumferential Subendocardium: longitudinal
Arrangement of fibers in the free wall	Transverse predominance	Olique predominance
Arrangement of fibers in the IVS	Obliquely, extending to the outflow tract	Obliquely
Contribution of IVS thickening in the transverse axis and shortening in the longitudinal axis in systole	+++	+++
Contraction pattern	Mainly longitudinal from base to apex	Subepicardium and Subendocardium: longitudinal shortening in opposite directions. Mesocardium: circumferential direction
Mass (g/m^2^)	26 (SD, 5)	87 (SD,12)
Wall thickness (mm)	2-5	7-11

*IVS: Interventricular septum*

### 13.3. Right Ventricle and Echocardiographic Parameters in Systolic Function Assessment

Several indices are routinely used to assess RV systolic function, such as fractional area change, tricuspid annular plane systolic excursion, tricuspid annular peak systolic velocity, and myocardial performance index. Each has its advantages and limitations, varying feasibility and reproducibility, and debatable diagnostic and prognostic efficacy.^35,36^ At present, no single method is considered a good indicator of RV systolic function. However, due to the orientation of the predominant longitudinal muscle fibers from the tricuspid annulus to the apex, preference is given to indices that explore movement along the longitudinal axis when assessing regional or global RV longitudinal function.^92^

Two-dimensional speckle tracking echocardiography is an imaging modality that evaluates myocardial deformation, an intrinsic property of the myocardium, in the 3 directions (longitudinal, circumferential, and radial). The longitudinal direction is the most common due to its good reproducibility, relevant prognostic information, validation in an experimental study^75^ and in clinical cardiac MRI studies of various cardiovascular diseases.^320-323^ Thus, RV 2D speckle-tracking can be considered a good marker of systolic function, with prognostic value in several cardiovascular diseases.^75,324-330^

### 13.4. Acquisition and Limitations

Determining RVGLS through 2D speckle tracking requires a modified apical 4-chamber window focused on the RV, with the transducer displaced more laterally and towards the right shoulder, which allows better visualization of the free wall and more reproducible measurements (Figure 13.1). It is important to optimize the orientation, depth, and gain to maximize the size of the RV, and to view its apex throughout the entire cardiac cycle.^36^ During image acquisition, the transducer should not be positioned anteriorly or posteriorly (avoiding the aortic valve and coronary sinus, respectively), showing only the interatrial septum.^331^ Once adequate visualization is obtained, the device should be set to record 3 cardiac cycles and acquire images at 50-80 frames per second. This rate can be obtained through indirect adjustments, such as image depth, resolution, and the ultrasound beam size, as well as by direct adjustment of the echocardiography device. Some software still requires the beginning and end of the RV ejection time to be defined, using pulsed Doppler echocardiography in the RV outflow tract.

**Figure 13.1:** – Imaging protocol for right ventricular (RV) strain measurement through speckle tracking echocardiography in an apical 4-chamber view: A) apical 4-chamber window (inappropriate acquisition method); B) apical 4-chamber window focused on the RV (adequate acquisition method). RVFWS: RV free wall strain; RVGLS: RV global longitudinal strain.

The region of interest is defined by the endocardial border, which includes the RV free wall and interventricular septum. It should not include the pericardium and its width should not to be too narrow, since this could lead to erroneous results. The baseline reference points must be positioned correctly; suboptimal positioning, eg, on the atrial side of the tricuspid annulus, could result in reduced LS values.^332^ The region of interest can be manually plotted or generated automatically; if generated automatically, the user must have permission to check and edit it manually as needed.^92^ After a tracking quality check and final operator approval, the regional strain values are displayed. According to current recommendations, the highest value reached during systole (peak systolic strain) should be used, with Doppler tracking of the pulmonary valve used to determine the end-diastole and end-systole.^92^ Whenever possible, appropriate software should be used, since the automatic detection algorithm of RV segments reduces the need for operator intervention, thus contributing to reproducible results.

The RV free wall is divided into basal, middle, and apical segments. The interventricular septum is similarly segmented. RV free wall LS is the mean strain value of all 3 segments, while RVGLS is the mean strain value of the free wall segments and the interventricular septum. RV free wall LS is more common in practice and clinical research, since LV function can interfere with RVGLS through the interventricular septum, leading to lower absolute values.^333^ RV free wall LS must be reported as a standard parameter, with RVGLS being optional.^92^

As limitations, in addition to inadequate acoustic windows, experimental studies and mathematical models have shown that the magnitude of myocardial strain is influenced by heart rate, in addition to preload and afterload. In preserved systolic function, studies have confirmed that strain can increase with higher preload and heart rate and can decrease when they are lower.^16,334^

### 13.5. Indications/Normal Values

The association between RV systolic dysfunction and poor prognosis in several cardiovascular diseases has been well-established, and RVGLS is an independent prognostic marker in pulmonary hypertension, HF, ischemic heart disease, and other cardiomyopathies; according to cardiac MRI, it is better correlated with right ventricular ejection fraction in than traditional parameters.^35,320-23,334-336^

RVGLS is lower in patients with pulmonary hypertension, showing good correlation with invasive hemodynamic parameters of RV performance.^337^ Furthermore, RVGLS has been found to be an independent predictor of all-cause mortality and pulmonary hypertension-related events. Aiming to assess the prognostic value of RVGLS in these patients, a recent meta-analysis found that a 19% relative reduction in RVGLS is associated with an increased risk of pulmonary hypertension-related events, while a 22% relative reduction is associated with a higher risk of all-cause mortality.^324^Figure 13.2 shows an example of RV strain in a patient with primary pulmonary hypertension.

**Figure 13.2:** – Example of right ventricular (RV) strain in a patient with primary pulmonary hypertension. TomTec software. RVFWS: RV free wall strain; RVGLS: RV global longitudinal strain.

In patients with HF, RVGLS has high sensitivity and accuracy for diagnosing RV systolic dysfunction.^338^ A recent study found that absolute values < 14.8% are associated with adverse events, such as mortality, heart transplantation, and hospitalization, regardless of LVEF or LV DD.^19^ In addition, RVGLS and RV free wall LS could detect subtle RV systolic function abnormalities in patients with HF and reduced LVEF, and to a lesser extent in those with HF and preserved LVEF.^329^ In patients eligible for LV assist device implantation, RVGLS is useful for stratifying the risk of RV failure. With a sensitivity of 68% and a specificity of 76%, an absolute RVGLS value < 9.6% can identify patients with post-procedure RV failure (ie, requiring an RV assist device or inotrope therapy > 14 days).^339^ In heart transplant patients, a combination of LVGLS and RV free wall LS measurements can help exclude acute cellular rejection and reduce the number of routine biopsies.^340^Figures 13.3 and 13.4 show examples of VR strain in a patient with a ventricular assist device and a heart transplant patient, respectively.

**Figure 13.3:** – Example of right ventricular strain in a patient using a ventricular assist device. GE EchoPAC software. AVC: aortic valve closure; GLS: global longitudinal strain.

**Figure 13.4:** – Example of right ventricular strain in a heart transplant patient. TomTec Software. RVFWS: right ventricular free wall strain; RV4CS: right ventricular global longitudinal strain.

In acute myocardial infarction, cardiac MRI RVGLS has the best correlation with right ventricular ejection fraction.^341^ It has been shown to be an independent predictor of death, reinfarction, and hospitalization due to HF, which confirms its fundamental role in assessing this population.^342^ A separate section will discuss the assessment of patients with arrhythmogenic RV dysplasia.

The role of RV systolic dysfunction has recently been investigated in other cardiomyopathies. In one study lower RVGLS values were found in an HCM group than in healthy controls,^343^ while another reported that RVGLS can differentiate HCM and hypertrophy secondary to hypertension with high sensitivity and specificity.^344^

The valvular heart disease with the greatest effect on the RV is mitral stenosis, frequently changing its conventional assessment parameters. The pattern of change in RVGLS is segmental, with significantly lower values in the interventricular septum and RV basal free wall, and normal values in the middle and apical free wall.^345,346^ Among patients with significant functional tricuspid regurgitation, RV free wall LS identified more individuals with RV dysfunction (84.9%) than fractional area change (48.5%) and tricuspid annular plane systolic excursion (71.7%). Furthermore, RV free wall LS was independently associated with all-cause mortality and had higher prognostic value when combined with traditional RV assessment parameters.^328^

Due to the lack of studies, there is no consensus about normal RV strain values. According to the most recent American Society of Echocardiography/European Association of Cardiovascular Imaging recommendations for echocardiographic cardiac chamber quantification in adults, RVGLS and RV free wall LS values below 20% are considered abnormal.^35^ However, this requires caution since different equipment types use different software programs, particular reference values, and differences in mapping level (endocardial, epicardial, or the entire myocardial wall).

Table 13.2 summarizes the main recommendations for GLS in RV assessment.

**Table 13.2:** – IIndications for global longitudinal strain in RV assessment

•Pulmonary hypertension (including patients with acute or chronic pulmonary embolism)
•Heart failure with reduced or preserved ejection fraction
•Acute myocardial infarction
•Cardiomyopathies (ADRV, HCM, DCM)
•Valvular heart disease (mitral stenosis, functional tricuspid regurgitation)
•Candidates for ventricular assist device implantation
•Heart transplant

*ADRV: arrhythmogenic dysplasia of the right ventricle; DCM: dilated cardiomyopathy; HCM: hypertrophic cardiomyopathy.*

## 14. Left and Right Atrial Strain

### 14.1. Left Atrial Strain Assessment Techniques

Strain analysis involves the 3 components of LA function: reservoir, conduit, and contractile. Strain rate is also used, although less commonly, ie, the peak strain rate during the reservoir phase, the conduit phase, and atrial contraction.^92,347^

Four- and 2-chamber apical images are optimized for the LA and taken at a high frame rate, usually 40-80 frames per second. A specific cardiac cycle is selected and point-to-point tracking is performed manually from the endocardial border of the mitral annulus to the opposite mitral annulus, extrapolating the entrance of the pulmonary veins and the left atrial appendage. The software creates the region of interest, which is adjusted to 3 mm in width and must include the endocardial and epicardial borders. If the tracking quality fails in ≥ 2 segments after manual adjustment, this incidence must be excluded from the analysis. Finally, the software calculates the GLS for each of the above-mentioned apical windows.

LA strain analysis can involve 2 different reference points: the beginning of the P-wave in the electrocardiogram^2^ or the R-wave peak of the QRS complex.^348^ The first method allows easier recognition of LA strain components, requiring the sum of the absolute values of LA conduit strain and LA contractile strain to obtain LA reservoir strain. The second method directly provides the LA reservoir strain value, which has the best prognostic value, while the other components are obtained from the graph. The most recommended and common method is to use the R-wave as a reference, since it has the smallest volume in the LA, and LA reservoir strain is thus more easily obtained.^92^

### 14.2. Normality Values

There is great heterogeneity in the literature regarding normality values for LA strain. A meta-analysis by Pathan et al. is the best evidence at the moment, which found the following mean values for LA reservoir strain, LA conduit strain, and LA contractile strain, respectively: 39.4% (95% CI: 38%–40.8%); 23% (95% CI: 20.7%–25.2%) and 17.4% (95% CI: 16.0%–19.0%).^37^

### 14.3. Clinical Applicability of Left Atrial Strain

LA strain assessment has shown greater prognostic value than volumetric measurement alone in several clinical contexts^349^ (Figure 14.1).

**Figure 14.1:** – Strain assessment of mechanisms involved in left atrial dysfunction.

#### 14.3.1. Left Atrial Strain and Diastolic Function in Heart Failure

LA strain is decreased in HFrEF and has prognostic value for all-cause mortality or rehospitalization for HF,^350^ good correlation with functional capacity^351^ and LV filling pressures,^352^ and is a good predictor of response to myocardial resynchronization therapy.^353^ In HFpEF, LA strain plays an important role in diagnosis^354^ and prognosis,^73,355^ and can predict the risk of AF.^98^

In about 20% of HFpEF cases, LV diastolic function may have an indeterminate pattern.^93^ LA strain can recategorize these patients,^90^ since the 3 atrial function components have shown good accuracy in determining increased LA pressure.^89^

#### 14.3.2. Atrial Fibrillation

In AF, the LA reservoir and conduit functions decrease and contractile function is absent. LA strain can predict new AF in several pathologies, such as HFrEF,^356^ mitral stenosis,^357^ and Chagas disease ^358^, as well as after pacemaker implantation,^359^ in addition to predicting AF recurrence after cardioversion^360^ or ablation.^361-363^ AT function assessment through strain could become part of the decision-making process for AF ablation. LA reservoir strain is also associated with ischemic stroke independent of CHA_2_DS_2_-VASc score, age, or anticoagulant use.^364^

#### 14.3.3. Valvular Heart Disease

LA strain may signal greater severity and an unfavorable course in mitral and aortic valve disease.^365,366^ In severe primary mitral regurgitation, LA reservoir strain can predict hospitalization for HF or all-cause mortality, regardless of whether surgical intervention is recommended.^367,368^

#### 14.3.4. Coronary Artery Disease

Coronary artery disease is associated with atrial dysfunction through 2 main mechanisms: LV DD and direct LA ischemia.^369^ LA strain may have important prognostic value in acute coronary syndrome, correlating with greater severity ^370^ and unfavorable outcomes.^371^

## 14.4. Right Atrial Strain

Although data on RA strain is lacking, a recent study of 101 healthy volunteers found the following values using the QRS complex as a reference: reservoir 37.6% (SD 6.9), conduit 26.0% (SD 7.1), and contraction 11.6% (SD 4.4).^37^ Assessing RA function is a target of interest in congenital heart diseases,^301,373^ tricuspid valve disease, and pulmonary hypertension.^374^

## 15. Assessing Left Ventricular Torsion

### 15.1. Introduction

LV function is determined through complex interactions between tissue anatomy, myocardial contractility, and hemodynamics. The muscle fibers of the LV myocardial wall are oriented in different directions. In the subendocardial region, they are almost parallel to the wall and move in a right-handed rotation (right-handed helix), which gradually changes in subepicardial fibers to 60–70º, leading to a left-handed rotation (left-handed helix).^375,376^

Subepicardial fiber contraction causes the apex of the LV to rotate counterclockwise and its base to rotate clockwise. Conversely, subendocardial fiber contraction causes the apex and base of the LV to rotate in opposite directions. Given the greater rotation radius of the epicardial layer, the direction of the subepicardial fibers prevails in the general direction of rotation when both layers contract simultaneously. This results in global counterclockwise rotation near the apex and clockwise rotation near the base during ventricular ejection,^377^ as shown in Figure 15.1.

**Figure 15.1:** – Rotation direction of subendocardial fibers: (A) subepicardial fibers (B) overall LV rotation with (C) simultaneous contraction of the fibers. Adapted from Stöhr et al.384

This twisting motion in the LV contributes to an even distribution of fiber shortening and stress along all walls, thus producing a relatively high EF (~60%) despite limited shortening (~20%).^378^ The twisting and shearing of the subendocardial fibers during ventricular ejection results in the storage of potential energy, which is subsequently used to unwind the coiled fibers during diastole, thus untwisting the helices, which together produce diastolic suction.^379,380^ Preload and afterload conditions and contractility alter the extent of ventricular torsion.^381^ Increased preload or contractility increases LV torsion, while increased afterload has the opposite effect.

Several imaging techniques can be used to quantify the mechanics of ventricular torsion: echocardiography (tissue Doppler, 2D and 3D speckle tracking, velocity vector imaging, cardiac MRI (tissue tagging), and sonomicrometry. Currently, there is no gold standard for assessing LV torsion mechanics, and the above-mentioned imaging modalities have good agreement.^382^ Due to its safety, availability, and cost-effectiveness, echocardiography is the most commonly used imaging modality.

### 15.2. Definitions and Nomenclature

Twist, twist rate, untwist, and untwist rate are the common terms for describing the systolic rotation and reverse diastolic rotation of the base and apex of the LV as seen from the apex. These terms are defined in Tables 15.1 and 15.2.

**Table 15.1:** – Definitions and parameters used to assess left ventricular twist during systole

Parameter	Definition
Apical rotation (º)	Peak counterclockwise systolic rotation of the apical LV region (measured in degrees)
Apical rotation rate (º/s)	Peak counterclockwise apical rotation speed (measured in degrees/second)
Basal rotation (º)	Peak clockwise systolic rotation of the basal region of the LV (measured in degrees)
Basal rotation rate (º/s)	Peak basal clockwise rotation velocity (measured in degrees/second)
LV twist (º)	Peak difference of LV apex and base systolic rotation (measured in degrees)
LV torsion (º/cm)	Normalized twist: ratio of the twist angle and the distance between the base and apex in systole (measured in degrees/centimeter)
Twist rate (º/s)	Peak LV twist speed (measured in degrees/second)

*(o): degrees; (o/s): degrees per second; (º/cm): degrees per centimeter. LV = left ventricle.*

**Table 15.2:** – Definitions and parameters used to assess left ventricular twist mechanism in diastole

Parameter	Definition
Apical reverse rotation (º)	Peak clockwise diastolic rotation of the apical LV region (measured in degrees)
Apical reverse rotation rate (º/s)	Peak velocity of clockwise apical reverse rotation (measured in degrees/second)
Basal reverse rotation (º)	Peak diastolic counterclockwise rotation of the basal region of the LV (measured in degrees)
Basal reverse rotation rate (º/s)	Peak basal rotation speed (measured in degrees/second)
Untwist (º)	Difference in peak reverse diastolic rotation of the apex and base of the LV (measured in degrees)
Untwist Rate (º/s)	Peak LV untwist speed (measured in degrees/second)

*(o): degrees; (o/s): degrees per second; LV = left ventricle.*

### 15.3. Step-by-step Assessment of Ventricular Torsion by Speckle Tracking Echocardiography

To evaluate the rotation mechanism, parasternal short-axis images of the LV are obtained at the basal (mitral valve) and apical levels (below the papillary muscles) (Figure 15.2). It is important to obtain apical images in which RV does not appear or only part of the LV appears, typically 1 or 2 intercostal spaces below the usual position. Most evaluation errors occur due to inappropriate selection of basal and apical planes and region of interest adjustment.

**Figure 15.2:** – Image acquisition plan for basal and apical rotation measurement (Images courtesy of Dr. Marcio Lima).

By convention, when rotation is clockwise, tracking begins below the baseline, and when rotation is counterclockwise, tracking begins above the baseline. (Figure 15.3). The normal global twist value is 9.7° (SD, 4.1°), although there are few reference values in the literature for torsion, which is estimated at 1.35°/cm (SD 0.54°/cm).^383^

**Figure 15.3:** – Clockwise (below baseline) and counterclockwise (above baseline) rotation (Images courtesy of Dr. Marcio Lima).

### 15.4. Clinical Applications

LV torsion parameters have generally been used to assess changes in ventricular mechanics in pathologies with reduced LVEF (ischemic and dilated cardiomyopathy) or preserved LVEF (HFpEF, hypertension, HCM, aortic stenosis, aortic insufficiency, and mitral insufficiency), as well as to assess subclinical myocardial dysfunction due to chemotherapy.

Twist and torsion measurements, although good parameters for global systolic function analysis, are limited in terms of reproducibility, which is mainly due to the lack of anatomical parameters for the apical section. The findings on ventricular twist and torsion changes are not specific, but they could contribute to a better understanding of the pathophysiology of different cardiomyopathies, helping differentiate them (Table 15.3).

**Table 15.3:** – Left ventriclar twist in different cardiovascular diseases

Cardiovascular disease	LV twist	Findings
Ischemic cardiomyopathy	Decreased	Twist decreases depending on the location and extent of ischemia.
Dilated cardiomyopathy	Decreased	Twist decreases proportionally to decreased ejection fraction
Hypertrophic cardiomyopathy	Increased	Twist increases especially if the LV outflow tract is obstructed
Aortic stenosis	Increased	Increased twist in cases of increased LV afterload.

## 16. Strain in Ventricular Dyssynchrony Analysis

### 16.1. Introduction

Due to its expressive reduction of morbidity and mortality, cardiac resynchronization therapy (CRT) is indicated in national and international guidelines. CRT is a class I recommendation for symptomatic dilated cardiomyopathy patients who are undergoing optimized clinical treatment and have a left bundle branch block (LBBB) pattern in electrocardiography, QRS duration ≥ 150 ms, and LVEF < 35% (evidence level A).^385^

Due to a lack of data about echocardiographic synchrony assessment, these guidelines consider LBBB ≥ 150 ms as a marker of dyssynchrony. However, echocardiography is also limited as a dyssynchrony marker. Thus, at present, echocardiographic dyssynchrony assessment for CRT selection must be performed in an individualized and judicious manner by an adequately trained examiner and interpreted together with the patient’s clinical data. It is also important to remember that not only can echocardiography assess cardiac synchrony but it can help select the best site for LV electrode implantation, as well as determine response and reverse remodeling. More recent findings indicate that it can identify ventricular arrhythmia risk.^385^

### 16.2. Dyssynchrony Assessment when Selecting Patients for Cardiac Resynchronization Therapy

Assessing mechanical dyssynchrony and efficiency through strain alone is insufficient to indicate CRT. However, even with a precise recommendation, the chance of success (ie, improvement in clinical, functional, and/or imaging variables) is approximately 60%–70%. The CRT response rate can be estimated and even improved through echocardiography. In this context, myocardial strain measurement stands out.

Initial analysis of dyssynchrony through radial strain describes the time difference between maximum radial strain of the middle anteroseptal and inferolateral segments. Values > 130 ms indicate patients with a higher response rate,^386^ as shown in Figure 16.1.

**Figure 16.1:** – Radial strain image showing curves for the middle segments of the anteroseptal and inferolateral walls. The interval > 130 ms correlates with a higher CRT response rate, in this case 340 ms.

In addition to radial dyssynchrony, independent predictors of long-term prognosis include an inhomogeneous pattern of septal rebound stretch in speckle-tracking and LV reverse remodeling with a higher value in LBBB and apical rocking detected by visual analysis. This pattern reflects incoordinated contraction, which results in reduced myocardial performance. Recent studies have indicated that septal rebound stretch assessment can improve patient selection for CRT, especially patients without defined LBBB.^387^ The pattern consists of 3 three aspects of LS in the inferoseptal and anterolateral (frequently basal) segments: 1) peak opposition of the septal curves (negative) and lateral curves (positive); 2) peak negative septal strain in up to 70% of the ejection time; 3) peak negative strain in the lateral wall after aortic valve closure,^388^ as shown in Figure 16.2.

**Figure 16.2:** – Longitudinal strain image with inferoseptal (yellow) and anterolateral (red) basal segment curves with typical left bundle branch block pattern: 1) initial opposition of the peak of the septal (negative) and lateral (positive) curves; 2) negative septal strain peak in ≤ 70% of the ejection time, with shortening interrupted during systole, ie, prior to aortic valve closure (AVC) resulting in systolic stretching; 3) negative strain peak in the lateral wall after AVC.

Global LVMW efficiency analysis has recently shown promise in CRT. Global LVMW efficiency can be quantified non-invasively through myocardial strain curves and blood pressure measurements. Lower global LVMW efficiency values have been independently associated with better long-term prognosis.^389^Figure 16.3 shows changes in strain, MW, and myocardial efficiency in a patient who underwent successful CRT.

**Figure 16.3:** – Pre- and post-procedure polar maps of (A) longitudinal strain, (B) myocardial work, and (C) myocardial efficiency in a patient who underwent successful resynchronization therapy.

### 16.3. Myocardial Viability Assessment

Another application of myocardial strain in the CRT context is the correlation between myocardial fibrosis and reduced strain values. Reduced global radial strain values correlate with a greater degree of fibrosis (detected in cardiac MRI), thus identifying patients with a lower chance of ventricular function recovery. Reduced LS in patients with ischemic heart disease can also be used for this purpose.

### 16.4. Electrode Implantation Site

Equally important as total LV fibrosis, compromised values at the LV electrode implantation site have been associated with lower CRT response. Studies have shown that positioning the LV electrode in the segment with the greatest delay in mechanical contraction results in higher CRT success rates. Speckle tracking can identify the segment that should be stimulated. In the TARGET study, LV electrode placement guided by 2D speckle-tracking resulted in better clinical response and lower rates of mortality and hospitalization for HF.^390,391^

### 16.5. Prognostic Assessment after CRT

Post-CRT dyssynchrony analysis through LS is a strong predictor of ventricular arrhythmias. Speckle-tracking findings of persistent or increased mechanical scattering 6 months after CRT are associated with worse prognosis. Furthermore, CRT response (reverse remodeling) depends on improvement in both longitudinal and circumferential function after CRT.^392^

### 16.6. Adjustment of Resynchronization Parameters

About 30% of patients who undergo CRT are considered non-responders due to a lack of clinical and/or functional improvement, including a lack of reverse remodeling (evidenced by lower ventricular dimensions and higher EF).^393^ In these patients, adjustments to the atrioventricular, interventricular, and left intraventricular intervals may improve CRT response. Some studies have shown that speckle-tracking can guide adjustment of CRT parameters, leading to significant improvement in functional class and EF in non-responders.^394^

## 17. Myocardial Work

### 17.1. Introduction

Having recently emerged as an echocardiographic tool to increase information about ventricular function, MW adds the effects of LV afterload to LS measurement. After the experimental work of Suga et al. in 1979,^395^ which demonstrated that the area under the pressure-volume curve, invasively acquired with an intraventricular conductance catheter, reflected regional MW and oxygen consumption per beat, interest has grown in non-invasive imaging methods to make such analysis feasible.^396,397^

In 2012, Russell et al.^398^ validated a non-invasive method of analyzing the LV pressure-strain loop, integrating systolic blood pressure at the time of speckle tracking LS assessment, which after interpretation by the software, calculates pressure-strain loops globally and per segment (Figure 17.1). The AUC represents MW, which is highly correlated with direct intraventricular measurements. MW also reflects regional myocardial oxygen metabolism comparably to positron emission tomography with 18F- fluorodeoxyglucose.^399-401^

**Figure 17.1:** – Left ventricular pressure-strain loop. Red: global pressure-strain loop. Green: pressure-strain loop for the basal segment of the inferolateral wall. AVC: aortic valve closure; AVO: aortic valve opening; IVCT: isovolumetric contraction time; IVRT: isovolumetric relaxation time LVP: left ventricular pressure estimated by systolic blood pressure; MVC: mitral valve closure; MVO: mitral valve opening.

Modest increases in blood pressure can reduce GLS by up to 9%, which could be misinterpreted as reduced contractility, when in fact MW remains preserved, reflecting only increased afterload. Thus, MW is considered an advance in the understanding of ventricular mechanics.^398,402^ The main differences between MW and the LV strain are shown in Table 17.1.

**Table 17.1:** – Main differences between myocardial work and left ventricular strain

	Strain	Myocardial work
AFI use	yes	yes
Measure	AVO-AVC	IVCT + ET + IVRT
Integrated with blood pressure	no	yes
Value	%	mmHg%
Incorporates postload	no	yes
Measures efficiency	no	yes
Estimates myocardial oxygen consumption	no	yes

*AFI: automated functional imaging AVO: Aortic valve opening; AVC: AV closure; IVCT: isovolumetric contraction time; ET: ejection time; IVRT: isovolumetric relaxation time.*

### 17.2. Calculating Myocardial Work

To obtain accurate and reproducible results, appropriate MW calculation techniques must be used for image acquisition, as well as post-processing and parameter analysis. This technology is currently only available in workstations or devices embedded with GE software (GE Healthcare, Horten, Norway). Analyses can be performed directly on the device or post-processed in workstations using previously acquired images.

The image acquisition protocol for calculating MW follows the same technical prerequisites for GLS analysis, which will be discussed in a subsequent chapter. After 2D strain is assessed using images acquired in the 3 apical projections through automated functional imaging, MW can be evaluated by the software (Figure 17.2). Blood pressure values measured non-invasively at the time of the examination must be manually entered into the patient identification form during the examination or later into the MW calculation form itself (Figure 17.3). These non-invasive blood pressure measurements will be automatically input into the pressure-strain loop.

**Figure 17.2:** – After assessing 2D LV longitudinal strain with the automated functional imaging method and processing the 3 apical windows, the software offers to calculate myocardial work (yellow arrow).

**Figure 17.3:** – Initial screen of myocardial work calculation. In this step, non-invasively measured blood pressure values can be inserted (bottom arrow), and cardiac events, such as the opening and closing of the mitral and aortic valves can be reassessed or marked (top arrow).

For temporal indexing of the values, the events of the cardiac cycle must be marked, identifying the opening and closing of the mitral and aortic valves. This can be performed through spectral Doppler or 2D analysis of mitral and aortic flow through an apical 3-chamber projection, which shows the opening and closing of both valves (Figure 17.4). These points can also be marked on the MW calculation screen, modifying apical 3-chamber image frame-by-frame, selecting the exact moment of each event (Figure 17.5). After checking the images and markings, the software performs the calculations and displays a polar (bulls-eye) map beside GLS and peak strain values for each segment. A polar map with the MW index for each segment appears on the right side, with global MW index and MW efficiency values below it. When the “work efficiency” key is selected, the software shows the global MW efficiency values for each segment on the polar map (Figure 17.6). Selecting the “advanced” key generates curves and graphs of the LV pressure-strain loops throughout the cardiac cycle, in addition to a bar graph showing the proportions of constructive and wasted MW (Figure 17.7).

**Figure 17.4:** – Marking cardiac events through spectral Doppler: A) marking mitral valve opening (MVO) and mitral valve closing (MVC) according to mitral inflow; B) marking aortic valve opening (AVO) and aortic valve closing (AVC) through LV outflow.

**Figure 17.5:** – Cardiac events can also be marked on the myocardial work calculation screen, modifying apical 3-chamber images frame by frame and selecting the exact moment of each event in the right sidebar menu (arrows).

**Figure 17.6:** – After approval of the obtained data, appropriately marking events and pressure filling, the software displays the global and segmental longitudinal strain values on the left (white arrows) and polar maps with myocardial work index values (A: yellow arrow) and myocardial work efficiency (B: blue arrow).

**Figure 17.7:** – Selecting the “advanced” key shows analyses by curves and graphs, showing the LV pressure-strain loops throughout the cardiac cycle (yellow arrow), in addition to a bar chart showing the proportions of constructive and wasted MW (blue arrow).

The following parameters are provided by the software:

1. Global MW index: total work in the area under the pressure-strain curve, calculated from the closing to the opening of the mitral valve. A bull’s eye map with segmental and global MW values is provided (Figure 17.6).
2. Constructive MW: work that contributes to LV ejection during systole, obtained by shortening during systole plus myocyte elongation during isovolumetric relaxation (Figure 17.7).
3. Wasted MW: work that does not contribute to LV ejection, obtained by myocyte elongation (rather than shortening) during systole plus shortening during the isovolumetric relaxation phase (post-systolic shortening) (Figure 17.7).
4. MW efficiency (MW efficiency/global MW efficiency): the result of the following formula:  constructive MW/  (constructive MW + wasted MW)  ; efficiency is rated as 0-100% (Figure 17.6).

### 17.3. Normality Values

Due to the recent validation of MW and its variables for clinical use, multicenter trials with an adequate number of patients have not yet been conducted to produce definitive normality values.

Manganaro et al. recently analyzed data from the NORRE study to establish normal reference limits for MW. This prospective European multicenter study included 226 patients from 22 echocardiography laboratories, providing reference values for most 2D and 3D echocardiographic data.^403^ The mean or median (SD) and/or CI of MW variables were, respectively, 1896 (SD, 308) mm Hg% (CI: 1292-2505) for global MW index, 2232 (SD, 331) mm Hg% (CI: 1582-2881) for constructive MW, 79 mm Hg% (CI: 53-122) for wasted MW, and 96% (CI: 94-97), for global MW efficiency.^403^

Another study found higher global MW index and constructive MW values in women > 40 years of age, with a strong correlation between a higher global MW index and constructive MW values and higher systolic blood pressure.^404^

### 17.4. Potential Clinical Use

A major limitation to more widespread use of MW is that the software is only produced by GE Healthcare. In addition, MW is calculated using manual systolic pressure measurements in afterload. Care should be taken in clinical situations involving additional afterload or increased systolic blood pressure, such as aortic stenosis, obstructive HCM, and some congenital heart diseases. Despite its promise as new tool, current scientific knowledge about MW assessment is still in the research phase.

Certain publications on MW in clinical practice are gaining notoriety, one of which involves patient selection for myocardial resynchronization. Although LBBB analysis can sometimes be difficult to interpret (Figure 17.8), visual and quantitative analysis of MW has made it easier to recognize cases of QRS widening without an associated mechanical dyssynchrony (Figure 17.9). In addition to visual analysis, the ability of constructive MW to identify CRT responders has been recognized as equivalent to contractile reserve.^405^ Another interesting way to identify patients who benefit from CRT is through wasted septal work.^406^ Thus, MW assessment in patients with LBBB can improve stratification through visual analysis, as well as quantify constructive and wasted work.

**Figure 17.8:** – Two cases with similar left bundle branch block morphologies.

**Figure 17.9:** – Case 1 has no changes in myocardial mechanics: myocardial work in the pressure-strain loop (green line) is similar to the global value (red line). In case 2, “true” left bundle branch block; in the same septal portion, the green line assumes a figure-8 shape.

Another interesting field for MW assessment is obstructive coronary disease. MW can detect obstructive coronary disease at rest better than LVGLS, even in patients with preserved EF and no segmental contractility changes.^407^ MW can also identify AMI patients at greater risk of complications and predict recovery,^408^ as well as predict long-term complications in patients with ST-elevation MI.^409^

In addition to these clinical situations, MW has shown promise for dilated cardiomyopathy, HCM, amyloidosis, and other conditions. Certainly, with greater software availability and more evidence in the literature, MW will quickly be incorporated into clinical practice.

## 18. Three-dimensional Strain Assessment: What can be Added

### 18.1. Introduction

Three-dimensional myocardial strain assessment through speckle tracking has numerous advantages over 2D assessment. Considering that the LV myocardium consists of 3 layers of fibers arranged in different directions (longitudinal, circumferential, and transverse), the speckles have a non-linear trajectory, escaping the image’s 2D plane during part of the cardiac cycle. Although multiple longitudinal and transverse images of the LV myocardium may be acquired in 2D assessment, the speckles are interpolated. Thus the method is not as accurate as 3D, which allows monitoring throughout the cardiac cycle in multiple dimensions, being unaffected by extraplanar myocardial torsion or apical shortening.^410^

In the RV, global analysis of myocardial chambers is only possible through 3D methodology, whereas only the septum and/or free wall can be assessed in 2D. Furthermore, 3D strain assessment is more reliable and physiological, since different components of myocardial strain are analyzed simultaneously through a single dataset or cardiac cycle. Thus, 3D strain is a quantitative, objective, comprehensive, and reproducible assessment of myocardial mechanical function. However, it strongly depends on a good acoustic window and a regular heart rhythm, which limit the routine and systematic incorporation of 3D strain.^411^ Clinical application is also limited by differences in the algorithms for monitoring the speckles and cut-off points for myocardial strain, which are not standardized across software platforms.^412,413^

Since 3D strain measurements obtained through different equipment and software manufacturers are not interchangeable, the baseline and follow-up imaging, as well as the analyses, must be performed using the same equipment, and the results must be interpreted in light of the equipment’s specific normality values.^410,412^ Normality reference values also vary between 2D and 3D methodologies, with only a modest correlation between LS values. Finally, further clinical research is needed to determine the accuracy and prognostic value of 3D strain assessment.

### 18.2. Left Ventricular Strain

Three-dimensional speckle-tracking is superior to 2D speckle tracking in that it is not limited to a single slice and that it allows analysis of vectorized data in 3 orthogonal planes. Two-dimensional strain assessment requires a very high temporal resolution (34-50 volumes per second) due to the short duration of the speckles (ie, a few milliseconds) in the slice, which does not occur in 3D. Additionally, the ideal in 3D speckle tracking is to acquire 6 beats at the highest line density and 44 volumes per second at a frequency of 2-4 MHz (which is more accurate than MRI). Acquisition of single volumes is not recommended, since higher temporal resolution decreases image and tracking quality by reducing line density.^414^

The general feasibility of 3D speckle-tracking is approximately 85% due to the following limitations: unfavorable acoustic windows, cardiac arrhythmias (preventing acquisition of multiple beats), incomplete visualization of the apical segments of the LV and RV, speckle-tracking problems in follow-up (distance from the transducer), and determining normal values and clinical prognosis.

### 18.3. Right Ventricular Strain

The analysis of RV contraction is especially important to understand the mechanism of this chamber in the face of congenital and acquired diseases. However, unlike the LV, estimating the RV is more difficult due to the complex shape that the RV presents and due to its thin wall. Despite this, MRI images and speckle tracking by echocardiography have been promising in right ventricular analysis. However, the values obtained by ST3D for the RV are still not well established.^415^ Technical problems persist for the 3D strain analysis when the objective is to analyze the right chambers, since the software was created for the analysis of the LV and still is adapted for the RV on most machines. Although the 3D strain allows global analysis of the entire right myocardium, the three-dimensional technology for this chamber is still in progress, with no well-defined cutoff values for the 3D strain of the RV so far.

#### 18.3.1. Full-volume 3D Acquisition and Analysis

Harmonic imaging ideally provides 4 triggered beats per capture. The depth must be adequate so that only the RV, its walls, and the tricuspid annulus fill the volume; the systolic peak strain is generally used for analysis.

## 18.4. Left Atrial Strain

In 3D strain assessment of the LA (as well as the ventricles), ultrasound can acquire volumetric data in real time and can measure all strain components. However, 3D tracking is a great challenge, and the temporal and spatial resolution of 3D is lower than 2D, which makes 3D analysis more complex and time consuming, since high image quality is required to determine 3D strain.

Another point under discussion is inter- and intraobserver variability in cardiac mechanics.^414^ The reservoir, conduit, and pumping phases can be well analyzed in 2D strain, and the mean cut-off values for 2D strain in each of these phases have been relatively well defined. However, reference values for 3D strain have not yet been determined. When strain analysis becomes relevant, the main applications of this method in the LV are: HFpEF,^7^ intracavitary filling pressures,^95^ atrial function among elite athletes,^416^ and cardiomyopathies.^417^ Nevertheless, 2D strain data have been better corroborated than 3D strain data; the incorporation of 3D strain in LA assessment will depend on future research.

## 19. The Role of Cardiac Resonance and Tomography in Strain Assessment

### 19.1. Introduction

Due to its high spatial and temporal resolution and non-invasiveness, cardiac MRI has become an important method of assessing global and segmental function in both ventricles. Strain assessment, an established and reliable method for measuring and quantifying regional and global contractile dysfunction, can detect subclinical cardiac dysfunction and, thus, is useful for assessing myocardial function. Echocardiography is currently the most available and least expensive method for assessing strain, but analysis may be impaired in patients with a limited acoustic window.

### 19.2. Strain Acquisition Methods by Cardiac Magnetic Resonance Imaging

Myocardial tagging, consisting of a preparation phase in which magnetic tags (black lines) are orthogonally superimposed on the myocardium at the beginning of a cine sequence, is the most validated technique for assessing strain in cardiac MRI.^12,418^ Alternatives to tagging that provide direct analysis of myocardial strain in cardiac MRI are cine strain encoding and cine displacement encoding with stimulated echoes*.*^419^ Feature tracking (FT), a recently developed method, can quantify myocardial strain in traditional cine cardiac MRI images without additional acquisitions or lengthy analysis.^420,421^

In all strain analysis techniques, the global circumferential and LS parameters have proven more reproducible and consistent than regional ones.^422^ Further details about strain acquisition through cardiac MRI can be found in the references.^12,418-422^

### 19.3. Determining Right Ventricle Strain Through Cardiac Magnetic Resonance Imaging

Myocardial strain measurement is an accurate and practical method for assessing RV function, since it is an earlier and more sensitive marker of contractile dysfunction than other methods, such as EF. Studies have demonstrated the potential of RV cardiac MRI strain to provide additional information and independent prognosis.^423-428^ A number of studies have analyzed RV strain in healthy individuals and control groups without heart disease.^423,426,429,430^

The pathologies that most affect RV, such as congenital heart disease, pulmonary hypertension, and arrhythmogenic dysplasia, have the greatest applicability in RV strain analysis. One study used cardiac MRI FT in patients with corrected tetralogy of Fallot, finding lower strain values in these patients than controls, which were related to systolic function parameters (biventricular EF), as well as functional capacity in cardiopulmonary testing.^425^

Assessing global and segmental RV function is fundamental for multi-parametric diagnosis of arrhythmogenic dysplasia, and RV strain has proven an extremely useful tool in this regard.^428,431^ Global and segmental RV strain are significantly lower in patients with arrhythmogenic dysplasia, regardless of RV dimensions and function, and impaired RV strain may represent an early marker of the disease.^428^Figure 19.1 presents examples of RV strain in a normal patient and a patient with pulmonary hypertension.

**Figure 19.1:** – Global longitudinal strain analysis through feature tracking in patients with (above) and without pulmonary hypertension (below). GLS was 4.26% and 26.46% in the patients with and without pulmonary hypertension, respectively.

### 19.4. Determining Left Ventricular Strain Through Cardiac Magnetic Resonance Imaging

In healthy individuals, mean values for LV strain types (global circumferential strain, global radial strain, and GLS) have been determined over the past decade with cardiac MRI FT,^419-423^ including a major meta-analysis.^424^ The largest and most recent studies on global circumferential strain and global radial strain by cardiac MRI FT analyzed the mean of 3 short-axis slices. In most cases, LVGLS was calculated using a 4-chamber slice, while more recent publications assessed the mean of 3 longitudinal slices. GLS and global circumferential strain values varied within a narrow range while global radial strain values had wider confidence intervals; it is speculated that planar movement and large personal variability could partially explain this phenomenon, although the real cause still remains uncertain.^424^

There is a strong relationship between myocardial strain and delayed myocardial enhancement, especially global circumferential strain, in addition to GLS derived from cardiac MRI FT. There is also good correlation between echocardiography-derived techniques and cardiac MRI.^425^

In dilated cardiomyopathy, markedly lower GLS is strongly associated with worse survival, even in patients with very low EF, regardless of functional class and other cardiac MRI findings.^426^ Cardiac MRI FT can identify HFpEF and DD subgroups through altered GLS.^427^

The diagnostic value of cardiac MRI GLS and echocardiography GLS are similar when differentiating between constrictive pericarditis and restrictive cardiomyopathy, having a high discriminatory value. GLS values are significantly lower in restrictive cardiomyopathy, while they are close to normal in pericarditis.^428^ Longitudinal and circumferential strain are also altered in myocarditis.^429^

Studies on MRI strain and myocardial tagging have found a high capacity to determine amyloidosis carriers; this method could even be more sensitive than the post-contrast sequence itself.^430^ The lost base-apex gradient of circumferential strain seems to be an early finding of Fabry disease, since longitudinal and circumferential strain did not vary significantly from healthy controls.^431^

According to cardiac MRI FT, patients with HCM have lower GLS, global radial strain, and global circumferential strain than healthy controls, with GLS and global radial strain being predictors of adverse events,^432^ just as significantly higher GLS has been found in hypertension patients than HCM patients.^433^

Ischemic coronary disease diagnosis by cardiac MRI can be improved by adding FT analysis, allowing detection of small changes in circumferential strain after dobutamine stress; GLS may be useful for detecting infarctions and assessing viability.^434^ The 3 types of strain are reduced in patients who have suffered acute myocardial infarction with ST-segment elevation, being independent predictors of adverse cardiovascular events.^435^

Patients with severe aortic stenosis have lower GLS and global circumferential strain than healthy controls, despite the symptoms.^436^ In patients with bicuspid aortic valve and preserved EF, signs of DD have been observed through circumferential strain changes.^437^ Chemotherapy-induced cardiotoxicity results in GLS and global circumferential strain abnormalities long before LVEF is reduced.^438^

A recent study reported that cardiac MRI FT-derived GLS has a stronger association with mortality than a combination of LVEF and myocardial delayed enhancement. This has been the largest study to date to assess prognosis through cardiac MRI FT-derived GLS. After adjusting for classic risk factors, including LVEF and myocardial delayed enhancement, a 1% worsening in GLS was associated with an 89% higher mortality risk in both ischemic and non-ischemic patients.^439^

### 19.5. Determining Left Atrial Strain Through Cardiac Magnetic Resonance Imaging

LA function assessment has been increasingly recognized as a factor in a variety of cardiac pathologies. Change in LA function is normally associated with worse prognosis and precedes HF diagnosis. The LA functions as a reservoir for pulmonary vein drainage, serving as a conduit for flow to the LV due to a pressure difference due to mitral leaflets opening and contractile function, with atrial systole occurring at the end of LV diastole.^440^

Cardiac MRI FT-derived atrial strain analysis reliably quantifies LA longitudinal strain and strain rate. Using standard cine MRI images, it can differentiate between patients with altered LV relaxation and healthy patients, as shown in Table 19.1.^441^ In a MESA substudy, global peak longitudinal atrial strain and LA volume indexes were independent predictors of HF onset, even after adjusting for LV mass and N-terminal pro–B-type natriuretic peptide.^442^ LA phasic function has been found to be an independent risk predictor for mortality or hospitalization for HF, even after adjusting for LA volume and ventricular remodeling.^443^

**Table 19.1:** – Left Atrial Strain

Type	HFpEF	HCM	Normal
Reservoir	16.3 ± 5.8	22.1 ± 5.5	29.1 ± 5.3
Conduit	11.9 ± 4.0	10.4 ± 3.9	21.3 ± 5.1
Pump	4.5 ± 2.9	11.7 ± 4.0	7.8 ± 2.5

HCM: Hypertrophic cardiomyopathy; HFpEF: Heart failure with preserved ejection fraction.

### 19.6. Determining Strain Through Cardiac Tomography

Strain can be assessed through cardiac tomography FT using contrast and triggered acquisitions, functionally reconstructing the cardiac cycle. Although data are still scarce, this method has been tested in patients with significant aortic stenosis undergoing transcutaneous aortic prosthesis implantation. Similar GLS values were found between cardiac tomography FT and echocardiography,^444,445^ including high intraobserver and intraclass reproducibility for cardiac tomography FT LVGLS, despite apparently underestimating the values.^444^

Another study explored the relationship between cardiac tomography FT-derived strain and ischemic heart disease in patients with significant lesions in the left anterior descending artery. Lower LS was observed in segments related to the anterior descending artery, despite normal diastolic and systolic volumes and EF.^446^

Currently, strain assessment through cardiac MRI and cardiac tomography is limited due to availability issues and the high cost of post-processing software. However, like strain assessed through echocardiography, its application has been well established, facilitating early diagnosis of dysfunction in various cardiomyopathies.
